# Genetic Variability of Hepatitis C Virus before and after Combined Therapy of Interferon plus Ribavirin

**DOI:** 10.1371/journal.pone.0003058

**Published:** 2008-08-26

**Authors:** José Manuel Cuevas, Manuela Torres-Puente, Nuria Jiménez-Hernández, María Alma Bracho, Inmaculada García-Robles, Boris Wrobel, Fernando Carnicer, Juan del Olmo, Enrique Ortega, Andrés Moya, Fernando González-Candelas

**Affiliations:** 1 Instituto Cavanilles de Biodiversidad y Biología Evolutiva and Departamento de Genética, Universidad de Valencia, Valencia, Spain; 2 CIBER en Epidemiología y Salud Pública (CIBERESP), Barcelona, Spain; 3 Unidad de Hepatología, Hospital General de Alicante, Alicante, Spain; 4 Servicio de Hepatología. Hospital Clínico de Valencia, Valencia, Spain; 5 Unidad de Enfermedades Infecciosas, Hospital General de Valencia, Valencia, Spain; University of Oxford, United Kingdom

## Abstract

We present an analysis of the selective forces acting on two hepatitis C virus genome regions previously postulated to be involved in the viral response to combined antiviral therapy. One includes the three hypervariable regions in the envelope E2 glycoprotein, and the other encompasses the PKR binding domain and the V3 domain in the NS5A region. We used a cohort of 22 non-responder patients to combined therapy (interferon alpha-2a plus ribavirin) for which samples were obtained before initiation of therapy and after 6 or/and 12 months of treatment. A range of 25–100 clones per patient, genome region and time sample were sequenced. These were used to detect general patterns of adaptation, to identify particular adaptation mechanisms and to analyze the patterns of evolutionary change in both genome regions. These analyses failed to detect a common adaptive mechanism for the lack of response to antiviral treatment in these patients. On the contrary, a wide range of situations were observed, from patients showing no positively selected sites to others with many, and with completely different topologies in the reconstructed phylogenetic trees. Altogether, these results suggest that viral strategies to evade selection pressure from the immune system and antiviral therapies do not result from a single mechanism and they are likely based on a range of different alternatives, in which several different changes, or their combination, along the HCV genome confer viruses the ability to overcome strong selective pressures.

## Introduction

The hepatitis C virus (HCV) infects approximately 170 million people worldwide and it establishes persistent infection in more than two-thirds of those who contract it [1], [2]. The current standard treatment for patients infected with HCV consists of a combined therapy of interferon plus ribavirin, which is successful in approximately 40% of the patients [3], [4]. Recently, randomised controlled clinical trials have shown that pegylated interferon and ribavirin yield a higher rate of sustained virological response [5], [6]. Moreover, response to anti-HCV therapy varies depending on the viral genotype. Patients infected with HCV genotypes 1 or 4 show significantly lower sustained response rates than those infected with genotypes 2 or 3 [6]–[9].

Studies trying to find differential patterns in the HCV genome in response to pressure from the immune system or resistance to antiviral treatment have mainly focused on those regions seemingly involved in evasion mechanisms and, in consequence, supposed to be subjected to strong selective pressures. The highest levels of sequence variation, i.e. the highest genetic plasticity, are concentrated in the four hypervariable regions of HCV, three of which are located in the envelope E2 glycoprotein. Hypervariable region 1 (HVR1) consists of a 27 amino acid sequence located at the N-terminus of the protein [10], [11] and seems to be involved in target-cell recognition and virus attachment [12]. Hypervariable region 2 (HVR2) consists of 9 amino acids located downstream of HVR1 [11] and it has been hypothesized to be involved in cell surface receptor binding [13]. Recently, a third hypervariable region, denoted HVR3 [14], [15], has been described in the E2 protein, and it is located between the two others. This region could play a role in the process of binding to host cell receptors and virus entry into host cells [14]. Finally, a fourth highly variable domain (V3), spanning 24 amino acids, is located at the C-terminus of the NS5A protein and appears to be involved in responsiveness to interferon [16], [17]. Close to this domain there is another important region in the NS5A protein, the protein kinase-R binding domain (PKR-BD), which consists of 66 amino acids and includes a 40 amino acid domain, the putative interferon sensitivity determining region (ISDR). These domains interact with PKR, which is involved in the cellular antiviral response induced by interferon, thus blocking its antiviral activity [18], [19].

HCV infections show two main features: high persistence and low susceptibility to antiviral treatments. The high levels of genetic variability of HCV are seemingly involved in viral escape from the host immune response, usually leading to chronic disease [20], [21]. The selective pressures exerted by the immune system [22], [23] correlate with the degree of genetic variability in the target regions [24]. This is the case for HVR1, which has been studied extensively [21], [25]–[28]. Moreover, the genetic variability of other regions such as the ISDR [29], [30] or the V3 domains [16], [17] has also been studied. However, no conclusive results have been attained in any case, probably due to the establishment of complex interactions between the genetic diversity of the virus and the host immune response [31]–[34].

In this study we have employed several analytical procedures to find differential selection patterns in a cohort of non-responder patients during their treatment with interferon alpha-2a plus ribavirin. For this, we employed 22 patients infected with HCV genotype 1 (7 with subtype 1a and 15 with subtype 1b), for which a sample immediately previous to initiation of antiviral treatment was available (T0), and additional samples after 6 or/and 12 months of treatment (T1 and T2, respectively), when their lack of response was established, were also available. Two viral genome regions were analyzed. About 100 clone sequences per patient were obtained from the E1-E2 region (4690 sequences in total), which included all hypervariable regions; additionally, between 25 and 96 sequences were obtained for the NS5A (2486 sequences in total), including the ISDR and V3 domains.

## Results

### Changes in nucleotide diversity during treatment

**Figure 1:**
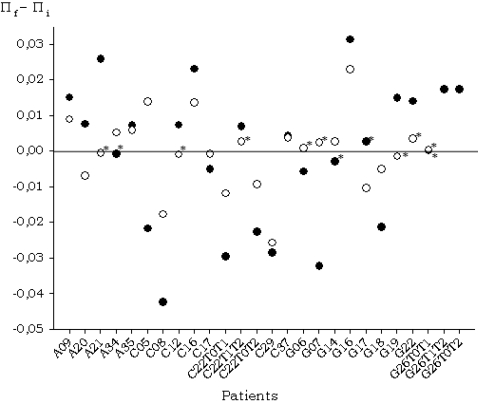
Graphic representation of the differences in nucleotide diversity between final versus initial time samples for the E1-E2 and NS5A regions (denoted as black and white dots, respectively). Three time samples were obtained for patients C22 (for E1-E2 and NS5A regions) and G26 (for E1-E2 region) and, consequently, differences between these three time samples are represented. Non-significant differences are indicated with an asterisk.

Nucleotide diversity was very high in the E1-E2 region (details of individual samples and different genetic variability estimates can be found in Table S1), although we found a few highly homogeneous samples (Table 1). A small positive correlation between nucleotide diversity estimates at the two time points (*r* = 0.218, P<0.01) was observed and the average values were almost identical (π = 0.0255±0.0031 for T0 samples and π = 0.0238±0.0033 for T1/T2 samples, Table 1). Differences in nucleotide diversity between T1/T2 and T0 samples were computed to analyze the change in diversity during treatment (Figure 1). Significant differences were detected in most cases (20/24) using t-tests. A significant increase in nucleotide diversity was detected in 13 cases, a significant decrease in 9 cases and no significant differences were found in 4 cases. Consequently, a slight tendency for nucleotide diversity to increase during treatment was detected for the E1-E2 region.

**Table 1:** Evolution of genetic variation within each patient during time for the E1-E2 and NS5A regions.

Patient	E1-E2 Region			NS5A Region		
	π (T0)	π (T1/T2)	Da (JC)	π (T0)	π (T1/T2)	Da (JC)
A09	0.0123	0.0274	0.0622	0.0123	0.0212	0.0070
A20	0.0240	0.0315	0.0014	0.0097	0.0028	0.0009
A21	0.0386	0.0644	0.0128	0.0029	0.0024	0.0009
A34	0.0292	0.0284	0.0001	0.0133	0.0184	0.0005
A35	0.0117	0.0189	0.0019	0.0061	0.0121	0.0001
C05	0.0468	0.0252	0.0212	0.0102	0.0240	0.0110
C08	0.0460	0.0037	0.0444	0.0187	0.0010	0.0127
C12	0.0248	0.0321	0.0304	0.0147	0.0138	0.0221
C16	0.0251	0.0482	0.0290	0.0116	0.0252	0.0083
C17	0.0079	0.0029	0.0185	0.0007	0.0000	0.0027
C22/T1	0.0431	0.0134	0.0226	0.0186	0.0067	0.0074
C22/T2	0.0431	0.0204	0.0197	0.0186	0.0093	0.0067
C29	0.0328	0.0043	0.0634	0.0297	0.0040	0.0356
C37	0.0084	0.0128	0.0008	0.0084	0.0122	0.0012
G06	0.0286	0.0230	0.0010	0.0262	0.0271	0.0011
G07	0.0398	0.0077	0.0369	0.0362	0.0386	0.0020
G14	0.0255	0.0226	0.0038	0.0108	0.0135	0.0008
G16	0.0000	0.0313	0.0737	0.0000	0.0230	0.0209
G17	0.0507	0.0532	0.0013	0.0154	0.0051	0.0087
G18	0.0314	0.0101	0.0318	0.0152	0.0102	0.0123
G19	0.0276	0.0425	0.0082	0.0106	0.0092	0.0050
G22	0.0146	0.0287	0.0086	0.0124	0.0159	0.0027
G26/T1	0.0003	0.0005	0.0085	0.0001	0.0005	0.0149
G26/T2	0.0003	0.0177	0.0032			

The nucleotide diversities (π) at the initial and final sampling times and the net nucleotide differentiation (Da, with Jukes-Cantor correction) are reported. For two patients, C22 and G26, the values of an intermediate sample (at T1) are also reported. (SD: standard deviation). Values from three different time samples are reported for two patients (C22 and G26) at the E1-E2 region and for one patient (C22) at the NS5A region.

In most patients we detected a significant divergence between sequences sampled before and after treatment. The corresponding nucleotide divergence statistics (Da), using Jukes-Cantor correction, are shown in Table 1. The range of Da values spanned from 0.0737 to 0.0002, with an average value of 0.0211 (0.0171 and 0.0231 for subtypes 1a and 1b, respectively). This is an indication that there are very different patterns of differentiation between sequences sampled at different times from the same patient, with a slightly larger effect for patients infected with HCV subtype 1b than subtype 1a (see below and supplementary Figures S1 and S2).

As expected, genetic variability was lower for the NS5A region than for the E1-E2 region (Table 1). A marginally significant correlation was found for nucleotide diversity between T0 and T1/T2 samples from each patient (*r* = 0.396, *P* = 0.056). Again, the average nucleotide diversities for the two sets of samples were almost identical (*π* = 0.0128±0.0089 for T0 samples and *π* = 0.0127±0.0098 for T1/T2 samples, Table 1). Variation in nucleotide diversity during treatment was evaluated by computing the difference between T1/T2 and T0 samples (Figure 1) for each patient and using t-tests. Significant increases in nucleotide diversity were detected in 8 cases, significant decreases in other 8 cases, and no significant differences in the remaining 8 cases. Therefore, the NS5A region did not present any trend in nucleotide diversity variation during treatment.

Similarly to the E1-E2 region, we detected a significant divergence between sequences sampled before and after treatment in most patients (Table 1, Figure 1). The range of Da values spanned from 0.0356 to 0.0001, with an average value of 0.0081 (0.0039 and 0.0103 for subtypes 1a and 1b, respectively). As for the E1-E2 region, these estimates indicate that there are very different patterns of differentiation between sequences sampled at different times from the same patient, with a wider range of variation for patients infected with HCV subtype 1b than for those infected with subtype 1a (see below and supplementary Figures S1 and S2).

For T0 samples, a significant correlation was detected between the nucleotide diversity levels of E1-E2 and NS5A regions (*r* = 0.604, *P*<0.002). However, no significant correlation was observed for T1/T2 samples (*r* = 0.092, *P* = 0.668). These results indicate that the levels of genetic variability between both regions were closely related before initiation of treatment, but the correlation had disappeared after it was discontinued.

We observed a significant correlation (*r* = 0.521, *P*<0.01) between the differences in nucleotide diversity at T0 and T1/T2 in the two genome regions analyzed in each patient. Only two patients (A20 and A35) presented significant changes of opposite sign in the two estimates, with diversity increasing during treatment in one region and decreasing in the other (Figure 1). Hence, there is evidence for nucleotide diversity being affected similarly by antiviral treatment in both genome regions, with only a few cases in which the two regions changed differently, at least in levels of nucleotide diversity, to treatment.

### Patterns and rates of evolution in the E1-E2 and NS5A regions before and after antiviral treatment

**Figure 2:**
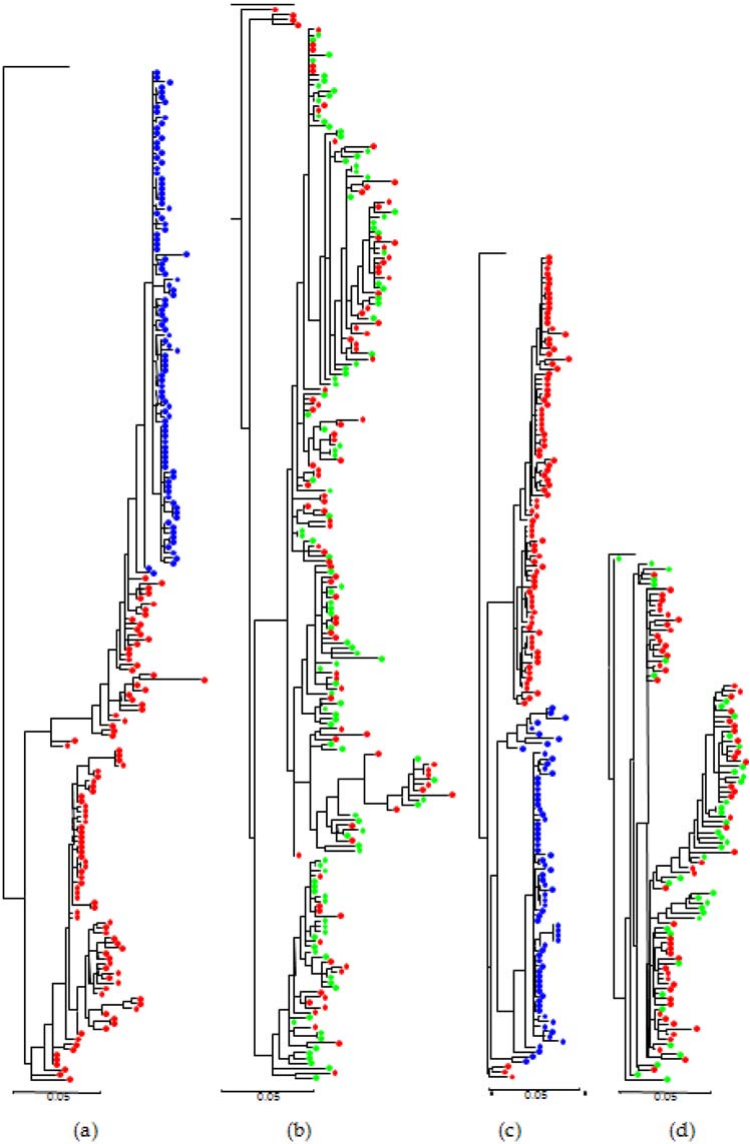
Examples of phylogenetic trees from the E1-E2 and NS5A regions. Trees from patients G07 (a) and A34 (b) are shown for the E1-E2 region, whereas trees from patients C12 and G06 are presented for the NS5A region. Red, green, and blue dots denote sequences from T0, T1, and T2, respectively.

We obtained maximum likelihood phylogenetic trees for all the sequence clones of each genome region from each patient using a common outgroup sequence, HCV-H77 (accession number NC_004102) for sequences of subtype 1a and HCV-J (accession D10749) for those of subtype 1b. The most remarkable feature from the analysis of these 44 trees was the absence of a common pattern for the relationship between clones derived before and after antiviral treatment (all trees for E1-E2 and NS5A regions are shown in the supplementary material, Figures S1 and S2). In the E1-E2 region, for instance, T0 and T2 samples from patient G07 were grouped in separate clusters, with all T2 samples clustering in a monophyletic group derived from one unidentified variant already present at time T0 (Figure 2a). On the contrary, for this same region clones from the two time samples from patient A34 were completely mixed, with no differentiation between T0 and T1 sequences (Figure 2b). Intermediate patterns were present in other patients for this region (Figure S1). The same observation applied to the NS5A region where, for instance, patient C12 showed a perfect separation in different clusters of sequences obtained at each time sample whereas patient G06 presented a very heterogeneous structure (Figure 2c, d). Again, a range of intermediate and similarly extreme patterns were found in other patients (Figure S2).

Inspection of the phylogenetic trees from both genome regions confirmed the previous results of nucleotide diversity analyses. Despite being characterized by a common phenotype, lack of response to interferon plus ribavirin treatment, hepatitis C virus from these patients presented a wide range of evolutionary patterns and levels of genetic diversity, before and after antiviral treatment. For instance, some patients showed low levels of genetic variability at the T0 sample and higher levels at T1/T2 samples, and vice versa (Figures S1 and S2). Globally, these results suggest that HCV populations use different strategies to adapt to and overcome the antiviral effects of interferon and ribavirin used in the treatment of these different patients.

To further characterize in a more quantitative manner the different adaptive strategies to antiviral treatment used by hepatitis C virus, we analyzed the amount of viral evolution within each infected patient in the two genome regions considered. For this, we analyzed the ML trees described above using common, epidemiologically unrelated outgroup sequences, H77 for subtype 1a sequences and HCV-J for subtype 1b.

**Figure 3:**
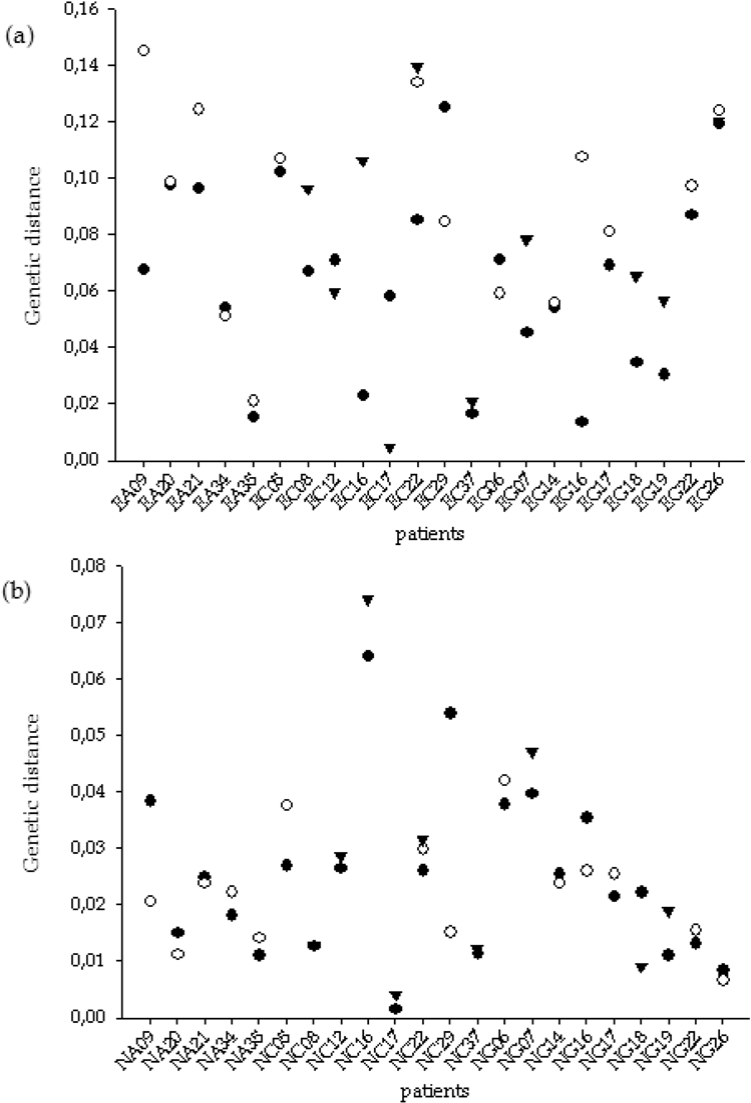
Mean genetic distances from the common ancestor of the sequences from the E1-E2 (a) and NS5A (b) regions for the different time samples for each of the 22 analyzed patients. Black dots, white dots and black triangles correspond to T0, T1 and T2 estimates, respectively.

As a proxy for the amount of evolution before and after antiviral treatment we computed the average length from the common ancestor to each tip for the different clones of each patient at both genome regions. In line with previous analyses, an enormous heterogeneity was observed for the cohort of patients in both regions (Figure 3). For the E1-E2 region, 17 out of 22 patients presented an increase of the genetic distance to the common ancestor during treatment, whereas the same happened for 14 out of 22 patients for the NS5A region. Despite this trend of increasing genetic distance from the origin after treatment, only 10 of the 22 patients showed a simultaneous increase in both regions, whereas the evolutionary distance decreased in both regions for only one patient. Contrary to the previous observation of lack of correlation between divergence before and after infection of each patient, we observed significant correlations between genetic distances from the common origin before and after antiviral treatment within each patient (*r* = 0.487, *P* = 0.011 for the E1-E2 region; *r* = 0.754, *P*<0.001 for the NS5A region). However, the relationship between genetic distance from origin before treatment and its relative change after it was the opposite one. Correlations between genetic distances at T0 and (T1/T2−T0) were negative and marginally significant for both genome regions (*r* = −0.407, *P* = 0.030, for E1-E2, and *r* = −0.303, *P* = 0.087 for NS5A). Finally, relative changes in both regions within patients were not correlated to each other (*r* = 0.080, *P* = 0.639) nor to the number of sites detected to evolve under positive selection (*r* = 0.328, *P*>0.10, for E1E2; *r* = 0.117, *P*>0.10, for NS5A).

These results provide further evidence for evolution taking place not only at different paces in the two genome regions considered – most values for the E1-E2 region were located in the range 0.04–0.10, whereas for the NS5A region most values were contained in the range 0.01–0.04 – but also more or less independently in each of them.

### Evolutionary changes during antiviral treatment in the E1-E2 region

The nature of the differences at the nucleotide level between viral populations sampled at different times during antiviral treatment within each patient was further investigated at the amino acid level. Firstly, we analyzed the differences in amino acid composition in each position between different samples for each patient. A summary of the significant differences found is shown in Supplementary Table S3. Almost half (71/154) of the positions were detected to vary significantly in composition in at least one patient. The tallying of positions with significant changes per patient also revealed a very uneven distribution, with a maximum of 29 positions (in patient G16) to a minimum of none found in two subtype 1a (A20 and A34) and two subtype 1b-infected patients (C37 and G17). There were no significant differences in the number of such positions detected between the two viral subtypes (average numbers were 12.5 for subtype 1a, 13.5 for 1b and 13.2 for the whole data set).

**Figure 4:**
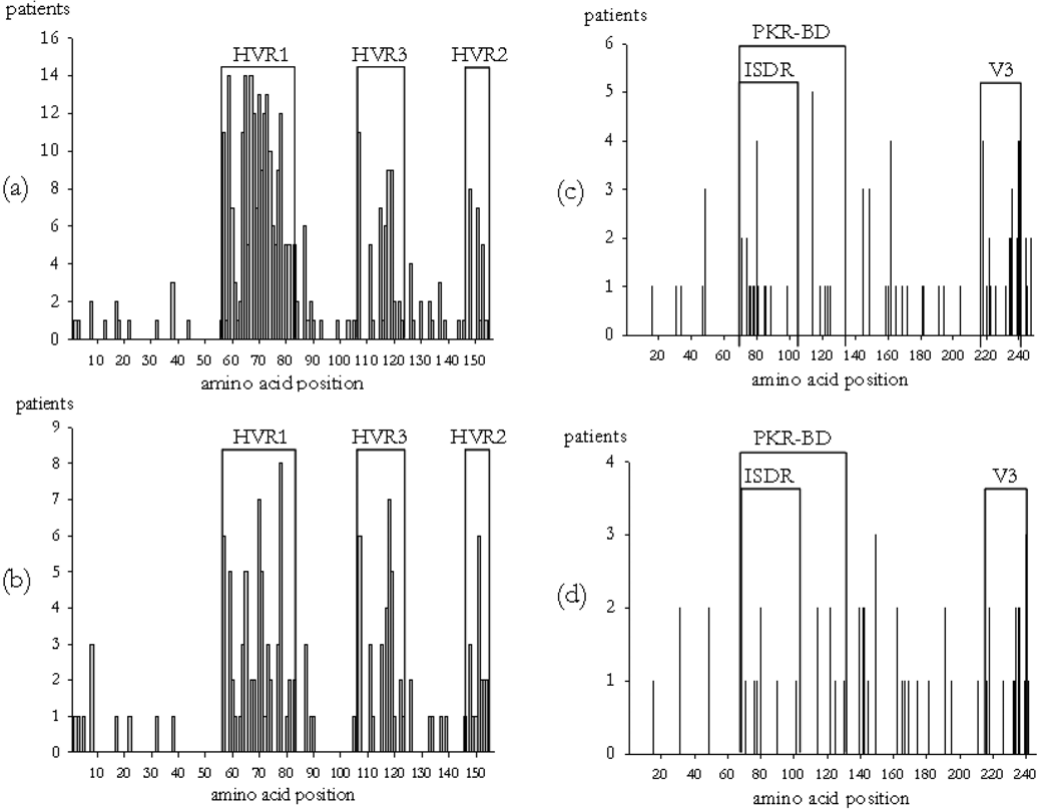
Positions with significant amino acid change and under positive selection for the E1-E2 (a and b, respectively) and for the NS5A region (c and d, respectively). Hypervariable regions for the E1-E2 region and ISDR, PKR-BD and V3 domain for the NS5A region are indicated.

As with genetic diversity at the nucleotide level, the distribution of significantly changing positions was not homogenous along the analyzed genome fragment (Figure 4a). For the E1-E2 region six sub-regions were defined, the three hypervariable regions described in this portion of the HCV genome (HVR1, HVR2 and HVR3) and the three intervening regions, the first one corresponding to the C-terminus of the E1 envelope protein coding gene and the two others already described in the E2 protein coding gene. In the 56 aa fragment corresponding to the E1 glycoprotein, significant changes were identified in only 11 positions, or 19.6% of the region (Table 2). Most of these positions were found to vary in a single patient (Figure 4a). In contrast, for the region corresponding to the E2 glycoprotein a high proportion of the positions were identified as variable in this analysis (59 of 101, 58.4%). These positions were mainly localized in the HVR1 region (92.6% of its positions differed significantly between the two time samples in at least one patient), the HVR2 (55.6%) and the HVR3 (64.7%). Furthermore, most positions identified as variable in these three regions were found in several patients simultaneously (Figure 4a).

**Table 2:** Summary of positions whose amino acid composition changes significantly during the treatment or detected to evolve under positive selection in the E1-E2 and NS5A regions analyzed in 22 patients.

					Positions identified to change in at least one patient	
Region	Sub-region		Positions	Na	Composition	Positive Selection
E1-E2	E1		1–56	56	11 (19.6%)	8 (14.3%)
	HVR1		57–83	27	25 (92.6%)	18 (66.7%)
	HVR3		107–123	17	11 (64.7%)	10 (58.8%)
	HVR2		147–155	9	5 (55.6%)	7 (77.8%)
	Remaining positions E2 protein			48	19 (39.6%)	8 (16.7%)
NS5A	NS5A_1		1–70	70	5 (7.1%)	4 (5.7%)
	PKR-BD	ISDR	71–110	40	11 (27.5%)	6 (15.0%)
		Rest	111–136	26	5 (19.2%)	5 (19.2%)
	V3 domain	218–241		24	12 (50.0%)	10 (41.7%)
	Remaining positions NS5A protein			87	16 (17.2%)	14 (16.1%)

(Na: number of amino acid positions in the corresponding region).

We next analyzed whether these changes in amino acid composition resulted from positive selection acting at the codon level or whether they could be explained by other factors. In 20 of the 22 patients the most likely evolutionary model for sequences sampled at two different time points (before and at the end of the viral treatment) incorporated a class of sites for which ω = Ka/Ks>1 and, in consequence, it was possible to apply the Bayes Empirical Bayes (BEB) procedure to identify those amino acid positions most likely under positive selection. The two exceptions corresponded to subtype 1b-infected patients, C29 and G22. Interestingly, whereas patient C29 showed significant change in 25 positions in the composition analysis, the four patients in which no such positions were found (patients A20, A34, C37 and G17) were all identified to evolve under the model incorporating positive selection.

Among the 20 patients for which the virus was estimated to evolve under a model incorporating positive selection, the BEB procedure identified 17 patients in which at least one codon had evolved under positive selection with a posterior probability >0.95. The number of such positions ranged between 1 (patient A21) and 26 (C08) (Table 3). Most positions evolving under positive selection in at least one patient were concentrated in a few quite well delimited regions (Tables 2 and 3 and Figure 4b). Many positively selected sites were identified as such in one single patient (22 sites), and only a few sites evolved under positive selection in 5 (4 sites), 6 (2 sites), 7 (1 site) and 8 (1 site) patients. Only three sites identified in more than one patient (positions 8 and 87, 3 patients; position 126, 2 patients) fell outside the three regions described above.

**Table 3:** Summary of positive selection analyses.

Patient	E1-E2 Model	Positions for E1-E2 region	pos. E1E2	ω	NS5A Model	Positions for NS5A region	pos. NS5A	ω
A09	M8	57, 137, 146	3	0.209	M8	240	1	0.112
A20	M8	70, 71, 78, 107, 111, 115, 123, 150, 151	9	0.326	M1*	N.C.	-	0.133
A21	M8	118	1	0.140	M8	49, 218	2	0.293
A34	M8	8, 38, 77, 78, 81, 118	6	0.178	M8	1, 76, 90, 233, 234	5	0.111
A35	M2	None	0	0.102	M1*	N.C.	-	0.128
C05	M3	3, 57, 59, 61, 65, 70, 77, 87, 105, 117, 118, 119, 151	13	0.242	M8	31, 71, 78, 80, 145, 169, 236, 241	8	0.147
C08	M3	17, 57, 59, 60, 64, 65, 67, 68, 70, 71, 74, 77, 78, 80, 81, 87, 107, 112, 115, 118, 119, 122, 134, 148, 151, 154	26	0.411	M8	122, 240	2	0.228
C12	M3	57, 59, 71, 83, 107, 153	6	0.136	M8	149, 216, 236	3	0.121
C16	M8	2, 64, 148, 151	4	0.202	M2	None	0	0.070
C17	M3	59, 65, 70, 73, 74, 78, 126	7	0.896	M0*	N.C.	-	0.883
C22	M3	8, 57, 70, 78, 107, 111, 115, 117, 119, 122, 133, 154	12	0.174	M3	114, 142, 162	3	0.150
C29	M1*	N.C.	-	0.242	M8	232, 240, 244	3	0.426
C37	M3	71, 87	2	0.185	M2	None	0	0.124
G06	M3	5, 139, 152	3	0.154	M8	15, 139	2	0.169
G07	M3	111, 118, 151	3	0.117	M8	130, 167, 195, 211	4	0.100
G14	M8	8, 32, 67, 70, 119	5	0.101	M2	None	0	0.076
G16	M3	60, 78, 153	3	0.271	M8	31, 49, 122, 125, 162, 165, 218, 234, 235	9	0.195
G17	M2	None	0	0.096	M8	132, 142	2	0.092
G18	M3	22, 73, 78, 107, 118	5	0.192	M3	80, 149, 191	3	0.161
G19	M2	None	0	0.123	M3	101, 114, 139, 149, 174, 191, 226, 239	8	0.133
G22	M7*	N.C.	-	0.111	M8	181, 235	2	0.197
G26	M3	57, 59, 63, 64, 65, 68, 70, 71, 73, 78, 83, 117, 120, 126, 148, 149, 151, 152	18	0.524	M0*	N.C.	-	0.156

For all patients, the best evolutionary model, the positions under positive selection, the number of positions (pos.) and ω = Ka/Ks estimates are indicated for both E1-E2 and NS5A regions. N.C.: non computable.

* Evolutionary models where positive selection is not computable.

Most, but not all, positions identified as evolving under positive selection in at least one patient corresponded to sites whose amino acid composition was found to be significantly different between the two different sample points in each patient (Table 3 and Table S3). The general distribution of these two kinds of sites was very similar (Figures 4a and 4b), with a lower number of sites identified to evolve under positive selection than changing in amino acid composition during the antiviral treatment.

For each of the six regions considered, levels of synonymous (*K_s_*) and non-synonymous (*K_a_*) substitutions were estimated for each patient (data shown in Table S4). A wide range of *K_s_* values, from 0 to 0.32 substitutions/site, was observed. Moreover, there were no clear differences among the different regions considered. On the contrary, *K_a_* values also presented a wide distribution, ranging between 0 and 0.27 subst./site, but in this case clear differences were observed among the six regions analyzed. Three different groups could be distinguished: firstly, the HVR1 region (with values ranging between 0 and 0.26 subst./site) showed the highest values for *K_a_*; secondly, regions HVR2 and HVR3, with *K_a_* values ranging from 0 to 0.1 subst./site, were characterized by similar and intermediate values of *K_a_*; and finally, a third group, comprising the three intervening regions and with *K_a_* values ranging from 0 to 0.05 subst./site, showed the lowest values of *K_a_*, very close to 0 in most cases.

The analysis of the changes in synonymous and non-synonymous substitutions between different samples from the same patient in the six sub-regions considered in the E1-E2 region allowed a better appreciation of the evolutionary processes involved in the virus response during treatment (Table S5). Globally, a general increase in the amount of synonymous substitutions was observed for the six sub-regions considered, ranging from 8% to 117%. This might be attributed to the mutagenic effect of ribavirin [59]. A more detailed examination of these results and those of changes in the Ka/Ks ratios revealed substantial variability in this response. Some patients showed increased levels of Ks after antiviral treatment in all, or most, sub-regions considered, but in others the pattern was the opposite one, with fewer synonymous mutations after treatment than before it (Table S5). Furthermore, in all but one case (patient G26) in which a significant number of positively selected sites were detected (Table 3) the pattern of change in Ks and Ka/Ks corresponded to a reduction in both parameters. The corresponding phylogenetic trees revealed highly monomorphic viral populations after treatment while those before this was initiated were very variable (see trees corresponding to patients A20, C08 and C17 in Supplementary Figure S1). Interestingly, a general trend towards negative correlations was found between Δ*K_s_* and the number of positively selected sites for each sub-region, but there were too few data points to test its significance reliably. This trend became marginally significant for some sub-regions when considering the total number of codons positively selected at the E1-E2 region (data not shown).

### Evolutionary changes during antiviral treatment in the NS5A region

We applied the same analyses previously described for the E1-E2 region to the NS5A region, although considering its specific features. Fewer positions were detected to vary significantly in composition in at least one patient in the NS5A region than in the E1-E2 region. Changes were observed in 49 of the 247 positions considered (19.8%), with an average of 4.5 patients per position. Again, the distribution of positions per patient was extremely uneven (Table S6), with a maximum of 22 positions (in patient C29) to a minimum of none, found in two subtype 1a patients (A34 and C17) and four of subtype 1b (A35, G06, G07 and G22). There were no significant differences in the number of such positions detected between the two viral subtypes (average numbers were 1.25 for subtype 1a, 4.44 for subtype 1b and 3.38 for the whole set).

The distribution of significantly changing positions was not homogeneous along the analyzed NS5A region, although to a lower extent than for the E1-E2 region (Figure 4c, Table 2). For the NS5A region we considered five different sub-regions: NS5A_1, ISDR, Rest of PKR-BD, NS5A_2, and the V3 domain. In the 70 amino acid fragment corresponding to the NS5A_1 region significant changes were identified in only 5 positions (7.1%), and all but one were detected in a single patient. For the PKR-BD, most variable positions were detected in the ISDR (11 of 40, 27.5%), when compared with the remaining positions within the PKR-BD (5 of 26, 19.2%). Remarkably, the highest proportion of variable positions was detected in the V3 domain (12 of 24, 50%), where most positions identified as variable were found in several patients simultaneously.

The best evolutionary model for sequences derived from each patient included a class allowing for positively selected codons (ω>1) in 18 of the 22 analyzed patients (Table 3). The four exceptions corresponded to subtype 1a-infected patients A20 and C17 and subtype 1b-infected patients A35 and G26. Among the 18 patients in which the virus was estimated to evolve under a model incorporating positive selection, in 15 of them we identified at least one amino acid with a posterior probability >0.95 of having evolved under positive selection, and the number of such positions ranged between 1 (patient A09) and 9 (patient G16).

In contrast to the E1-E2 region, the distribution of sites evolving under positive selection was relatively homogeneous (Tables 2 and 3 and Figure 4d). There were only two remarkable regions: the one denoted as low variability region, which showed a very low number of positively selected sites (4 of 70, 5.7%), and the V3 domain, which showed the highest proportion of positively selected sites (10 of 24, 41.7%). The frequency of sites evolving under positive selection was very similar between the PKR-BD and the remaining positions of the NS5A region. In any case, it is also important to note that most positively selected sites were detected in only one patient (24 of 39, 61.5%), 13 sites were detected in two patients (33.3%), and only two sites were detected in three patients (5.1%). In analogy with the E1-E2 region, most positions identified as evolving under positive selection in at least one patient corresponded to sites whose amino acid composition was found to be significantly different during antiviral treatment (Figure 4c, d).

We observed a wide range of *K_s_* values, ranging between 0 and 0.2 subst./site, but without clear differences among the different regions considered. The distribution of *K_a_* values was not as wide as for the E1-E2 region, ranging between 0 and 0.05 subst./site, but again in this case significant differences were observed among the five regions analyzed. Three different groups could be distinguished (Supplementary Table 7): firstly, the V3 domain (with *K_a_* = 0 to 0.05 subst./site) showed the highest values for *K_a_*; secondly, the regions termed Rest of PKR-BD and NS5A_2 (with *K_a_* = 0 to 0.02 subst./site) were characterized by similar and intermediate values of *K_a_*; and finally, a third group, comprising the NS5A_1 and the ISDR, with *K_a_* = 0 to 0.007 subst./site, showed the lowest values of *K_a_*, except for four cases at the ISDR showing intermediate or even high *K_a_* values.

A detailed analysis of the pattern of evolutionary changes in each of the five sub-regions considered within NS5A (Supplementary Table S8) revealed a similar pattern to that observed in the E1-E2 region, but with a general decrease in values for all parameters. Overall, there was a small increase in the levels of synonymous substitutions and a slight decrease in the change of Ka/Ks before and after treatment. However, there was no clear relationship between the direction of change in the levels of synonymous substitutions and the detection of positively selected codons (Table 3, Table S8), not even for the three patients with a significantly larger number of such positions detected (C05, G16 and G19, with 8, 9, and 8 positions, respectively). Once again, the corresponding phylogenetic trees showed markedly different patterns with one patient, C05, presenting similarly variable, non monophyletic groupings for sequences obtained before and after treatment, another patient, G17, with a relatively more monomorphic viral population after treatment, and yet another, G06, with an almost monomorphic population at T0 replaced by a highly variable one after antiviral therapy (Figure S2).

## Discussion

Lack of response to antiviral treatment is presumably associated with the ability of viral populations to escape from the deleterious effects of the effective agent(s). For such apparently simple organisms as viruses, the escape response depends on the existence of resistance mutations at different genome locations. Tremendous efforts have been devoted to identify which particular mutations are responsible for HCV resistance to interferon and ribavirin, which has resulted in the identification of several genome regions presumably associated to viral escape but no specific variant(s) have been found to consistently produce this phenotype. Nevertheless, several reports, including our own, have described that the levels of genetic diversity in different HCV genome regions correlate with treatment failure, with higher variability levels before treatment in isolates from non-responders than from responders [35]–[38].

In this work, we have observed a wide range of genetic variation in non-responder patients in viral populations sampled before initiation of antiviral therapy in the two genome regions analyzed, E1-E2 and NS5A. Hence, genetic diversity in these regions does not seem to be a good predictor of sustained viral response to antiviral therapy, since all these patients were non-responders. Furthermore, we have shown that there is not a single, common pattern in the variation of the nucleotide diversity during the antiviral treatment, especially in the NS5A region, although the E1-E2 region presents a slight trend to increase genetic diversity (Figure 1 and Tables 1 and 2). Therefore, our results show that neither the genetic diversity level nor its rate or pattern of change during treatment can be taken as predictors for the response to antiviral treatment because they are different for different patients despite these showing the same outcome.

The absence of a common response to antiviral treatment in these viral populations extends not only to genetic variability but also to more general patterns of evolution. This is reflected in a wide diversity of patterns in the phylogenetic trees derived for the two genome regions from the viral sequences obtained before and after treatment. Within-patient phylogenetic trees of the infecting viruses presented from very homogeneous, highly differentiated populations at the two sampling points, to cases in which both constituted a single, highly variable population with no signs of differentiation between the two samples. All intermediate possibilities were also found, including cases with an almost monomorphic initial population which was transformed into a highly variable one, to the reverse case.

This lack of a common pattern is also revealed by the detection of positive selection in these two regions. Although most patients presented positively selected sites at one or the other genome region, no such sites were detected in patient A35. On average, the number of such sites was higher in the E1-E2 than in the NS5A region, but there was no correlation within patients (*r* = −0.0567, P>0.10, Table 3). Patients with a large number of positively selected sites in the E1-E2 region showed none (A20, G26) or few (C08) such sites in the NS5A but patient C05, third in the ranking of sites in E1-E2, was second in NS5A. The reverse is also true for patients with most selected sites in the NS5A region.

These differences are likely reflecting the different role of the two fragments analyzed. If we consider the distribution of changes along the E1-E2 region, it is remarkable the high degree of conservation of the first 56 amino acids, which correspond to the C-terminus of the envelope 1 protein (Table 3 and Figure 4). This fragment presents a hydrophobic region apparently involved in multiple functions, such as the maintenance of E1 and E2 proteins in the endoplasmic reticulum or the formation of heterodimers between E1 and E2 proteins [39]–[41], suggesting its involvement in the viral replication cycle and accounting for the high degree of conservation detected therein. In contrast, the sequenced portion of this region that encodes the E2 protein presents a higher level of variability, mainly concentrated in the three hypervariable regions. The highest number of changes has been found in the HVR1, where most HCV antigenic sites have been reported [42], [43]. Hypervariable regions HVR2 and HVR3, in which several antigenic sites have been reported [13], [44] also show high levels of variability, although to a lower extent than HVR1. The recently described HVR3 [14], [15] shows a slightly lower variability than HVR1 and HVR2, which correlates with a lower exposition of its antigenic sites as inferred from structural models [13]. This pattern is also reflected in the analysis of genetic divergences, which showed the highest *K_a_* for HVR1, intermediate levels for HVR2 and HVR3, and very low levels for the remaining sub-regions included in this E1-E2 fragment (Table S4).

Levels of genetic variability in the NS5A region were lower than in the E1-E2 fragment analyzed. The first 70 amino acids of the NS5A region showed a high degree of conservation, whereas the highest variability was found in the V3 domain, which has been postulated to be involved in responsiveness to interferon [16], [17], [45] and where a certain degree of variability has been described [46]. The remaining sub-regions in this fragment showed an intermediate degree of variability. The analyses of genetic divergences further corroborated these observations, showing the highest *K_a_* for the V3 domain, the lowest values for the first 70 amino acids of the fragment and the ISDR (with some exceptions), and intermediate *K_a_* values for the rest of the fragment (Table S7). On the whole, these and other similar results [47] indicate that the NS5A protein is subject to strong evolutionary restrictions, probably because of its role in replication mechanisms [48], [49]. Moreover, the low levels of variability present in the PKR-BD, and more specifically in the ISDR, are probably due to the existence of a specific sequence involved in response to interferon [18], [29], [50], [51].

A correlation in the number of amino acid changes between both regions was observed for composition analysis but not for positive selection analysis. This could reflect the presence of different selective pressures acting on each region, and consequently of hitchhiking phenomena. The absence of recombination in HCV along with the enhanced selective pressures during antiviral treatments would facilitate the presence of hitchhiking selection [52], [53], in which the regions under the strongest selective pressures would drive the course of evolution in the rest of the genome. In this situation, the level of linkage between regions would depend on the time elapsed between the hitchhiking events and the subsequent readjustment phenomena in the affected regions. Although the high mutation rates in the HCV genome will certainly complicate these analyses, the role of hitchhiking selection in the evolution of HCV deserves a closer scrutiny.

Genetic variability, amino acid composition and positive selection analyses reflect the enormous heterogeneity of adaptive solutions shown by viral populations infecting each patient. These results are further corroborated by the phylogenetic analyses, where the diversity of tree structures in the pool of patients for both analyzed regions is remarkable, thus precluding to discern general patterns of viral adaptation. Additionally, the analysis of divergence is consistent with the previous results, providing evidence for the particular adaptation routes exhibited by each patient.

In agreement with our results, it has been previously shown that the adaptive solutions adopted by RNA virus populations are convergent to a certain extent [54]. However, although positions detected to evolve under positive selection are mainly concentrated in the hypervariable regions, there are too many of these so to establish clear patterns of adaptation to the strong selective pressures exerted by the immune system and antiviral drugs. At this point, it is important to remark the difficulty in distinguishing between changes due to selective pressures imposed by the immune system from those specific to antiviral therapy.

The addition of ribavirin is likely to mask adaptive events even further. The precise mechanism of action of ribavirin is not completely understood [55] and different mechanisms have been recently shown. It has been suggested that the anti-HCV effect of ribavirin is partly mediated via the up-regulation of PKR activity [56]. Alternatively, it has been proposed that ribavirin acts as an RNA mutagen [57], in which case a possible mechanism for resistance could depend on increasing replication fidelity by means of the accumulation of mutations in the polymerase [58]. In fact, the mutagenic effect of ribavirin has been confirmed very recently [59], although this is still a controversial issue [60]. We have detected a global increase in the levels of synonymous substitutions after failed treatment, which could be due to the mutagenic effect of ribavirin. However, as indicated above, there are cases in which the change is in the opposite direction. But we have also found that the detection of large numbers of positively selected sites in the E1-E2 region is usually associated to a reduction in the level of synonymous substitutions and to a less polymorphic viral population after treatment. The most plausible interpretation for this is that the stronger the selective pressures on viral population (imposed by antiviral treatment and host immune response), the higher the initial reduction in genetic variability. Alternatively, for those populations with more positively selected sites, an increased fidelity of the corresponding HCV polymerase could also account for the observed reduction in the levels of synonymous substitutions. In this respect, mutations in the NS5B protein, which is the RNA-dependent RNA polymerase in HCV, could be under strong selective pressure and, consequently, variation in other genome regions, such as the hypervariable regions, could eventually become a surrogate marker of these selection events. From this perspective, future studies should also focus on the genetic analysis of the NS5B protein and its potential correlation to sensitivity to ribavirin [61].

## Materials and Methods

### Patients and samples

Serum samples were obtained from 22 patients infected with HCV genotype 1, seven of which were infected with subtype 1a and 15 with subtype 1b. These patients were included in a prospective study in which serum samples (T0 samples) were taken immediately before they were subjected to a combined treatment of pegylated interferon-2a plus ribavirin. After 6 or 12 months, treatment was discontinued since viral load did not decrease more than 2 logs and a second serum sample was obtained for analysis (T1 and T2 samples for 6 and 12 months, respectively). In a few cases, samples were available for both 6- and 12-months time points. Samples were obtained in different hospitals from the Comunidad Valenciana, Spain (Table 4). All patients provided written consent to be included in the study which was approved by the corresponding ethics committees of the institutions involved (Hospital General de Valencia, Hospital Clínico Universitario de Valencia and Hospital General de Alicante).

**Table 4:** Main features of HCV samples included in this study.

Hospital	Patient identifier	Available sera	# of sequences (E1-E2)	# of sequences (NS5A)	HCV subtype
H1	A09	T0,T1	100, 114	32, 34	1a
	A20	T0,T1	112, 98	67, 33	1a
	A21	T0,T1	100, 108	49, 64	1b
	A34	T0,T1	100, 112	29, 39	1a
	A35	T0,T1	109, 113	27, 25	1b
H2	C05	T0,T1	100, 100	86, 43	1b
	C08	T0,T2	100, 100	77, 76	1b
	C12	T0,T2	100, 100	92, 71	1b
	C16	T0,T2	101, 107	74, 74	1b
	C17	T0,T2	106, 100	25, 85	1a
	C22	T0,T1,T2	100, 100, 101	44, 25, 55	1a
	C29	T0,T1	100, 100	87, 60	1b
	C37	T0,T2	101, 100	42, 49	1b
H3	G06	T0,T1	100, 100	61, 43	1b
	G07	T0,T2	100, 100	48, 49	1b
	G14	T0,T1	100, 100	84, 86	1a
	G16	T0,T1	103, 100	57, 47	1b
	G17	T0,T1	101, 100	68, 29	1b
	G18	T0,T2	100, 100	60, 71	1b
	G19	T0,T2	100, 100	52, 56	1a
	G22	T0,T1	100, 102	36, 49	1b
	G26	T0,T1,T2*	100, 100, 102	84, 42	1b

Abbreviations: H1, Hospital General de Alicante; H2, Hospital Clínico de Valencia; H3, Hospital General de Valencia; T0, serum obtained before starting combined therapy with interferon alpha plus ribavirin; T1, serum obtained six months after starting combined therapy with interferon alpha plus ribavirin; T2, serum obtained twelve months after starting combined therapy with interferon alpha plus ribavirin.

* T2 sequences were not available for the NS5A region.

Two HCV genome regions were studied: one corresponded to a 472 nt fragment encompassing genes encoding proteins E1 and E2 (from nucleotide 1322 to 1793 in the HCV-J reference genome sequence, accession number AF009606 [62], including the three hypervariable regions HVR1, HVR2 and HVR3), and referred to as E1-E2 region, and the other corresponding to a 743 nt fragment from gene NS5A (nucleotides 6742 to 7484), including the interferon sensitivity determining region (ISDR) and the V3 domain and referred to as NS5A region.

### Experimental procedures

RNA extraction, reverse transcription, amplification, cloning and sequencing, are described in detail in [63]. Briefly, after viral RNA extraction (High Pure Viral RNA Kit; Roche), reverse transcription reactions were performed with random hexadeoxynucleotides in order to prevent any bias during reactions due to unspecific oligonucleotides. Primers used for subsequent PCR are detailed in [64]. Amplified DNA products for each region were purified with High Pure PCR product Purification Kit (Roche) and directly cloned into EcoRV-digested pBluescript II SK (+) phagemid (Stratagene). Plasmid DNA was purified with High Pure Plasmid Isolation Kit (Roche). Cloned products for E1-E2 and NS5A regions were sequenced using vector-based primers KS and SK (Stratagene). For the E1-E2 region, we obtained about 100 clones from each patient, yielding a total of 4690 sequences, 2232 from T0 samples, 1447 from T1 samples and 1011 from T2 samples. For the NS5A region, we obtained between 25 and 96 clones per sample and 2486 sequences in total were determined (see Table S1 in Supplementary data). HCV sequences obtained in this study have been deposited in GenBank with accession numbers given in Tables S1 and S2.

### Genetic variability analysis

Sequence alignments were obtained using CLUSTALX v1.81 [65]. DnaSP 3.51 [66] was used to estimate, for both E1-E2 and NS5A regions, the following measures of genetic variability in the viral samples of each patient: number of polymorphic sites (S), total number of mutations (η), number of haplotypes (nHap) and nucleotide diversity (π).

### Synonymous (K_s_) and nonsynonymous (K_a_) substitutions

Synonymous (*K_s_*) and nonsynonymous (*K_a_*) substitution per synonymous and nonsynonymous site, respectively, were estimated for each patient from data derived from the corresponding T0 sample using the Nei-Gojobori method implemented in the program MEGA [67]. Standard errors of *K_s_* and *K_a_* were obtained by bootstrap resampling with 500 pseudoreplicates. According to structural and functional properties, the 472-nt fragment of the E1-E2 region was divided into six different sub-regions for *K_s_* and *K_a_* estimation: the E1 sub-region, corresponding to E1 protein (nucleotide positions 2 to 169, amino acid positions 1 to 56 [positions 328–383 in the HCV-J reference sequence]); the HVR1 (nucleotide positions 170 to 250, amino acid positions 57 to 83 [384–410]), the E2_1 sub-region, comprising nucleotide positions 251 to 319 (amino acid positions 84 to 106 [411–433]); the HVR3, defined between nucleotide positions 320 to 370 (amino acid positions 107 to 123 [434–450]); the E2_2 sub-region, comprising nucleotide positions 371 to 439 (amino acid positions 124 to 146 [451–473]); and the HVR2 (positions 440 to 466 nt, 147 to 155 aa, [474–482]). Similarly, the NS5A region was subdivided into five different sub-regions for *K_s_* and *K_a_* estimation: the NS5A_1 sub-region (nucleotide positions 3 to 212, amino acid positions 1 to 70 [2139–2208]); the ISDR (nucleotide positions 213 to 332, amino acid positions 71 to 110 [2209–2248]); the rest of the PKR-BD (nucleotide positions 333 to 410, amino acid positions 111 to 136 [2249–2274]); the NS5A_2 sub-region (nucleotide positions 411 to 653, amino acid positions 137 to 217 [2275–2355]); and the V3 domain (nucleotide positions 654 to 725, amino acid positions 218 to 241 [2356–2379]). For both regions, *K_s_* and *K_a_* estimates were obtained for each of the delimited sub-regions.

### Changes in amino acid composition during treatment

For both regions, amino acid composition was determined for each sample and the different sets of sequences corresponding to each patient (T0 sample *versus* T1 or T2 sample) were compared with program VESPA [68]. Tests for differences in the composition at each amino acid position between the two time-points were carried out by means of a *G*-test. Significance levels for multiple comparisons were corrected by Bonferroni's method.

### Positively selected amino acid positions during the treatment

For each patient, a maximum likelihood approach [69] implemented in the PAML package 3.15 [70] was used to investigate the presence of positively selected codons in the E1-E2 and NS5A regions. Two criteria were employed to assign the best evolutionary model to each patient (independently for each region): a likelihood ratio test (LRT), which compares the fit of two nested models to the data [69]; and the Akaike information criterion (AIC), which allows to perform comparisons between non nested models [71]. For all patients and genome regions, six models were compared with the PAML package: M0, M1, M2, M3, M7 and M8. For models M2, M3 and M8, the existence of positively selected codons is allowed as they incorporate a class of codons for which ω = *K_a_*/*K_s_* (ratio of non-synonymous and synonymous substitution rates) can be >1. Therefore, whenever one of these models explained the observed data significantly better than the other corresponding alternative in which such a class is not allowed, then the existence of positively selected codons was inferred. Next, a Bayes empirical Bayes (BEB) procedure [72] was applied to detect codons with a posterior probability of belonging to the ω>1 class larger than 0.95.

### Phylogenetic trees and rates of molecular evolution

Maximum likelihood trees were constructed with PHYML [73] using a common evolutionary model (GTR+I+G) and common outgroup sequences, H77 (accession number NC_004102) for subtype 1a sequences and HCV-J (accession number D90208) for subtype 1b. These two outgroup isolates represent epidemiologically unrelated strains to those included in our study. HCV-H77 was isolated from an American patient in 1979 [74] and HCV-J was derived from a Japanese patient in the late 1980's [75]. Rates of evolution for the different time samples of each patient were estimated by removing the outgroup from each phylogenetic tree and then computing the average length of the arms for all the sequences from each time sample.

## Supporting Information

Figure S1 Phylogenetic trees for the E1-E2 region from all 22 analyzed patients. Different symbols are used to denote sequences sampled at T0 (red dots), T1 (green dots) and T2 (blue dots).
(0.36 MB PPT)

Figure S2 Phylogenetic trees for the NS5A region Phylogenetic trees for the NS5A region from all 22 analyzed patients. Different symbols are used to denote sequences sampled at T0 (red dots), T1 (green dots) and T2 (blue dots).
(0.37 MB PPT)

Table S1 Genetic variability measures in the E1-E2 region of the HCV genome.
(0.09 MB DOC)

Table S2 Genetic variability measures in the NS5A region of the HCV genome.
(0.09 MB DOC)

Table S3 Positions detected to change significantly in amino acid composition between samples at T0 and T1/T2 for each patient included in the study for the E1-E2 region.
(0.26 MB DOC)

Table S4 Synonymous and non-synonymous substitutions levels in the six sub-regions of the E1-E2 region.
(0.09 MB DOC)

Table S5 Relative change in the levels of synonymous and non-synonymous to synonymous substitutions in the six sub-regions of the E1-E2 region.
(0.09 MB DOC)

Table S6 Positions detected to change significantly in amino acid composition between samples at T0 and T1/T2 for each patient included in the study for the NS5A region.
(0.18 MB DOC)

Table S7 Synonymous and non-synonymous substitutions levels in the five sub-regions of the NS5A region.
(0.08 MB DOC)

Table S8 Relative change in the levels of synonymous and non-synonymous to synonymous substitutions in the six sub-regions of the E1-E2 region.
(0.08 MB DOC)
