# Supplementary material for: Genetic Variability of Hepatitis C Virus before and after Combined Therapy of Interferon plus Ribavirin
Source: PLoS One. 2008 Aug 26;3(8):e3058. doi: 10.1371/journal.pone.0003058 (PMC2518109; doi:10.1371/journal.pone.0003058)
Supplement: Figure S1 — Phylogenetic trees for the E1-E2 region from all 22 analyzed patients. Different symbols are used to denote sequences sampled at T0 (red dots), T1 (green dots) and T2 (blue dots). (0.36 MB PPT) [file pone.0003058.s001.ppt]

## Slide 1
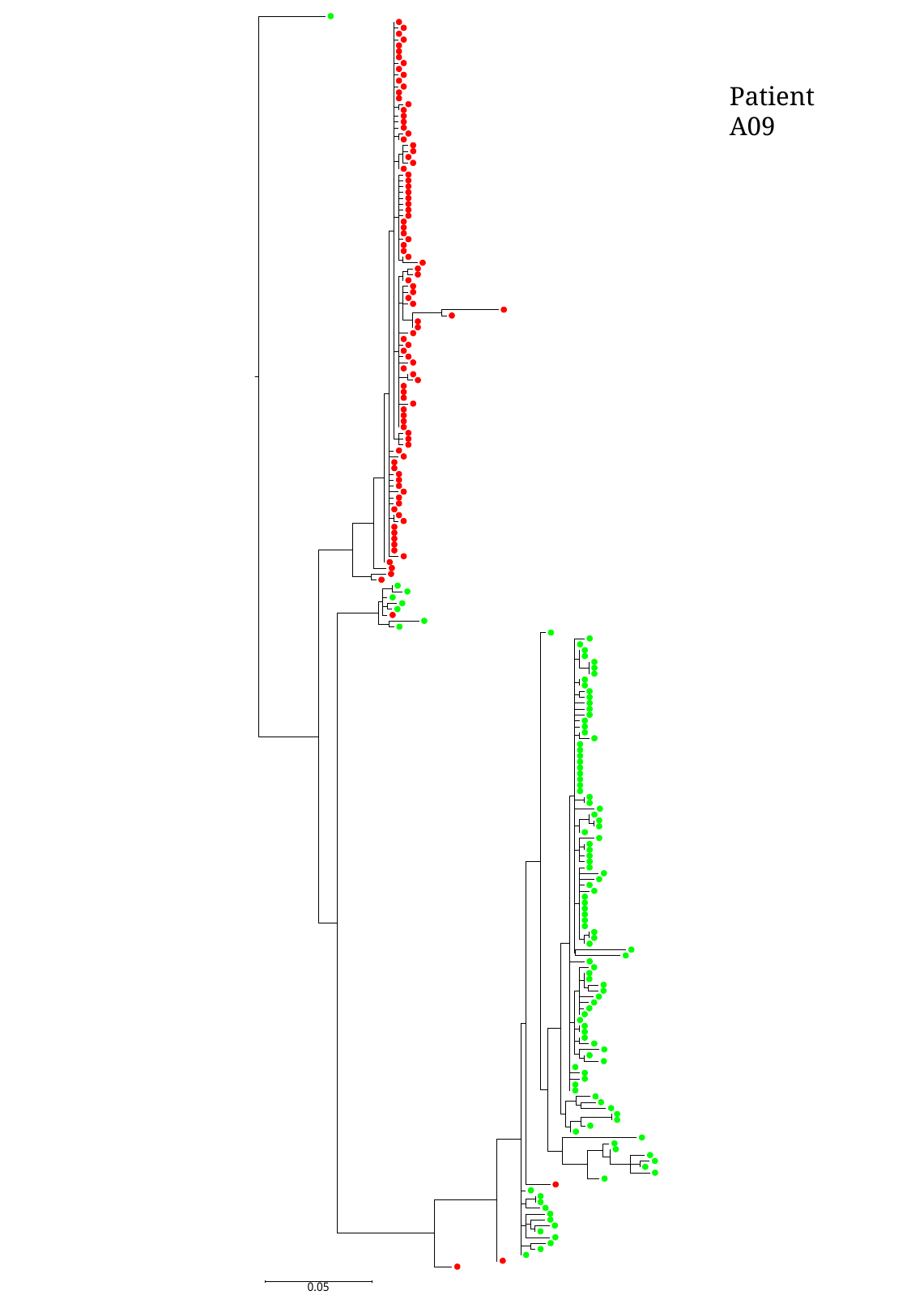

Patient A09

## Slide 2
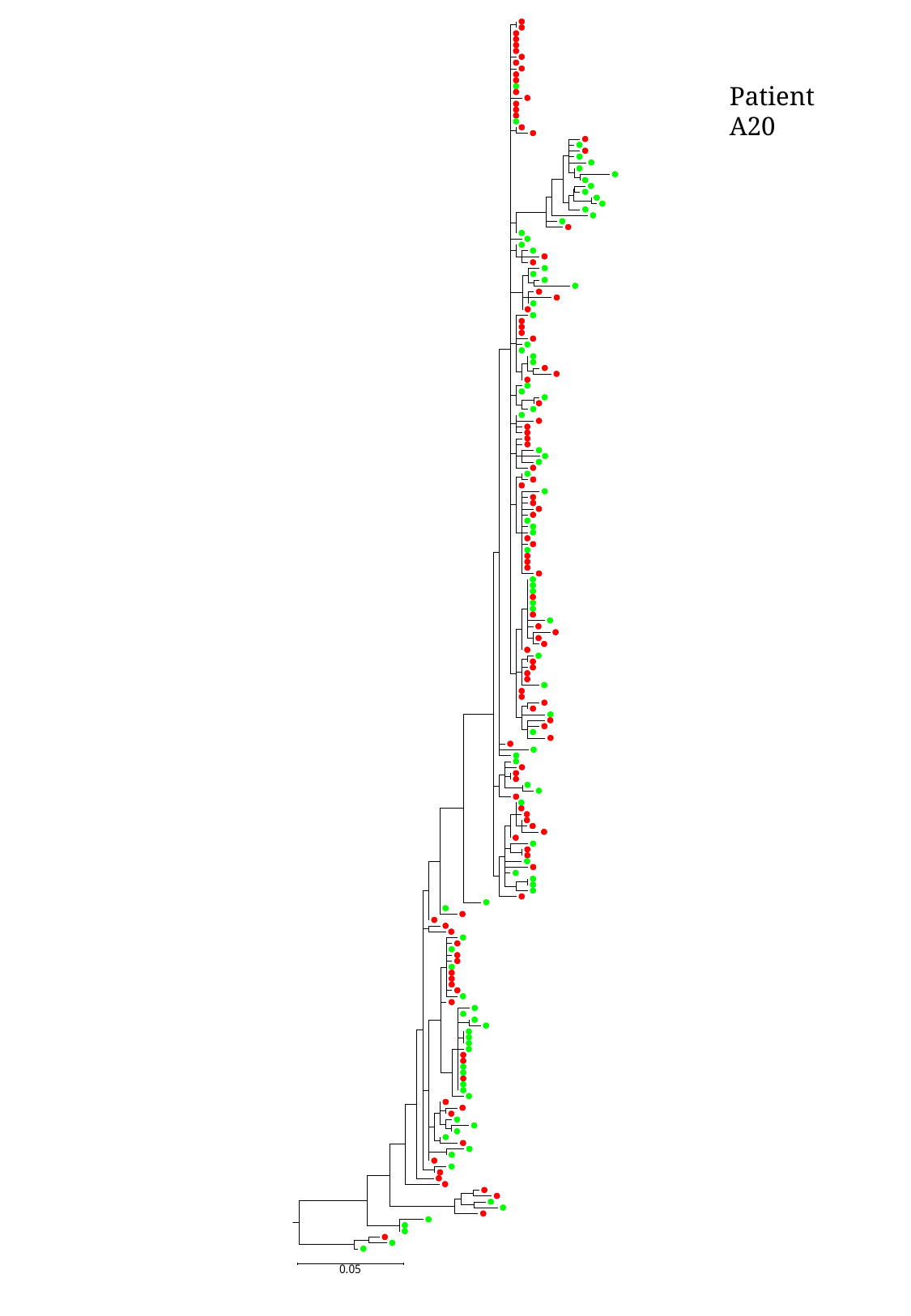

Patient A20

## Slide 3
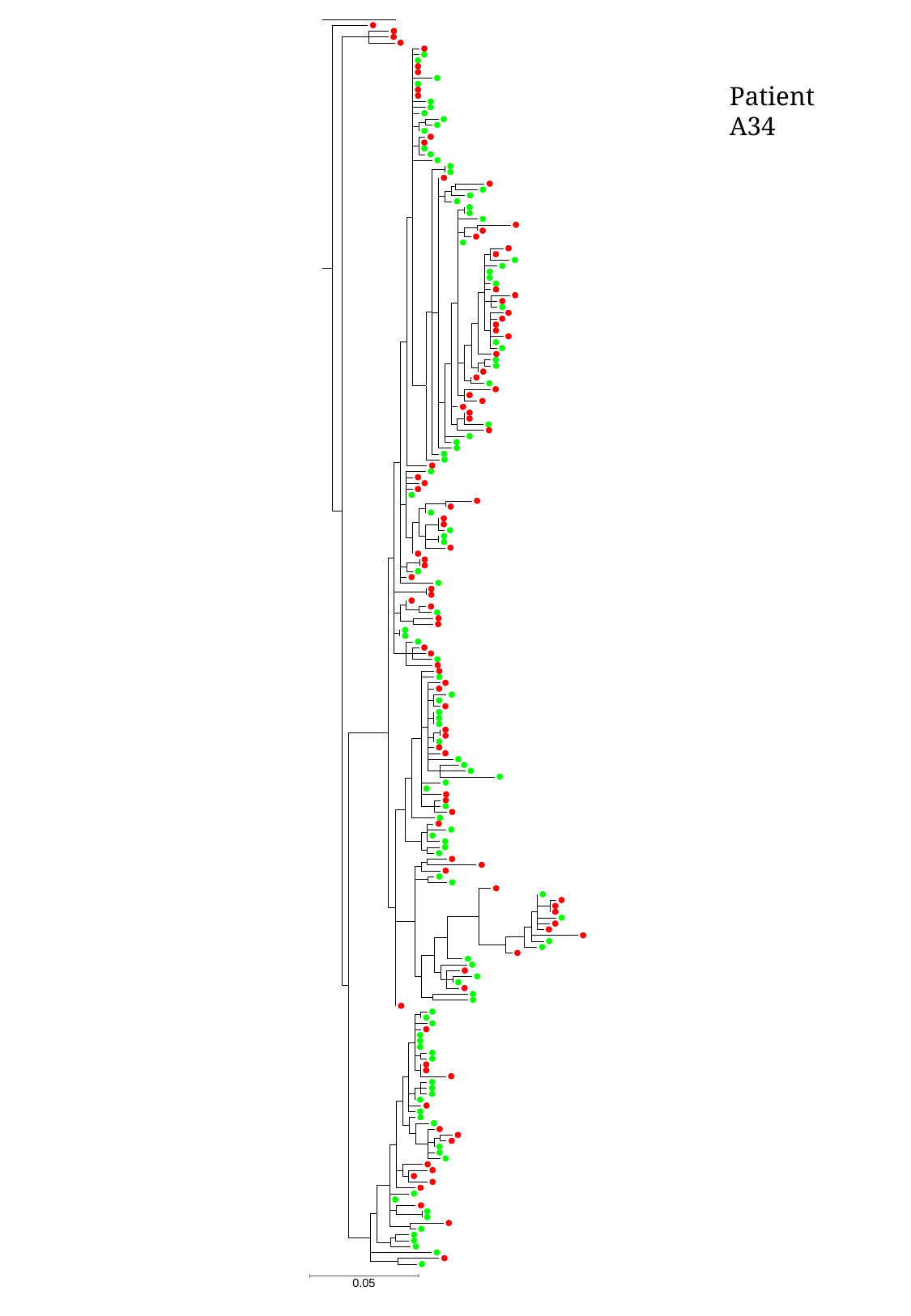

Patient A34
0.05

## Slide 4
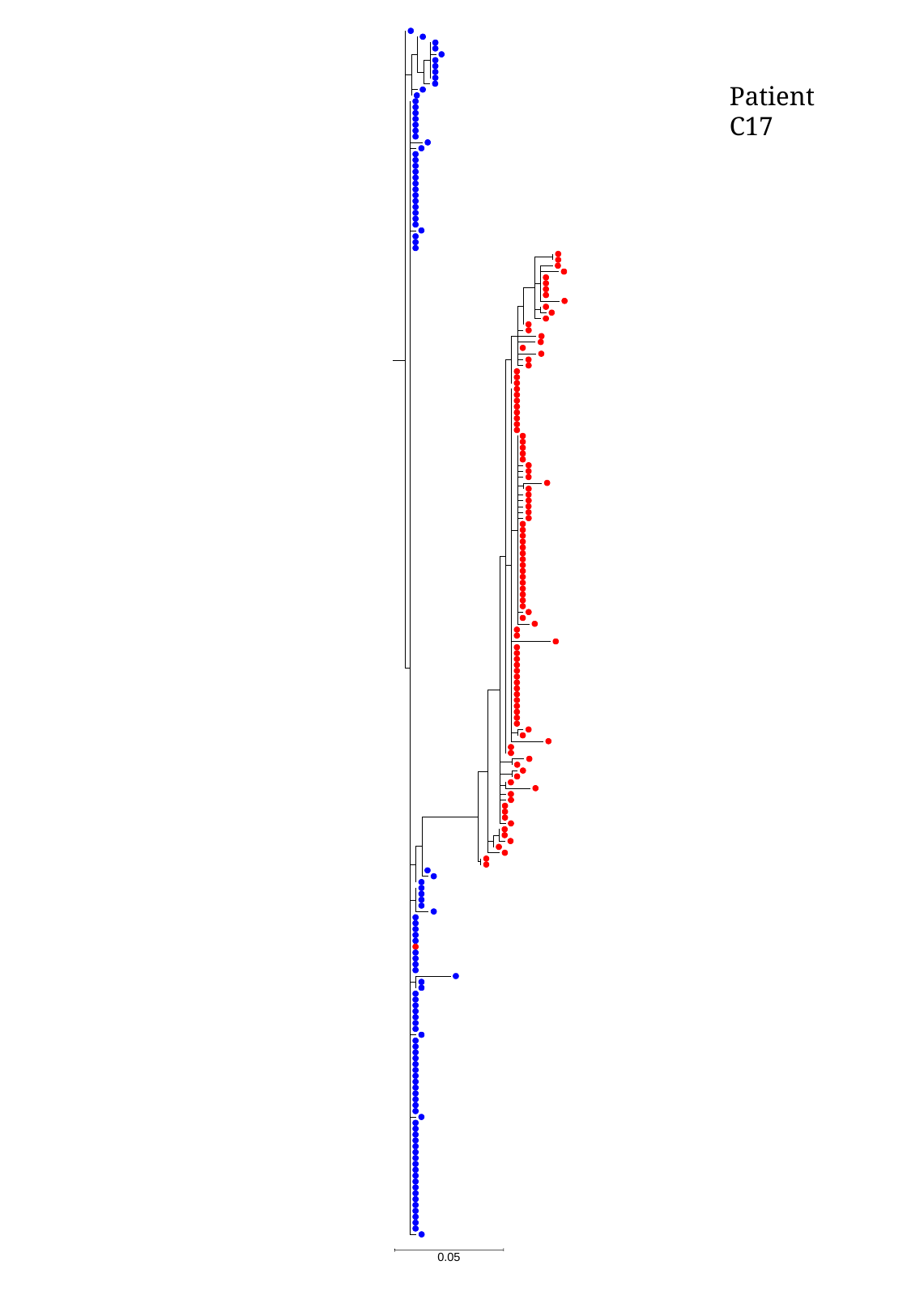

Patient C17
0.05

## Slide 5
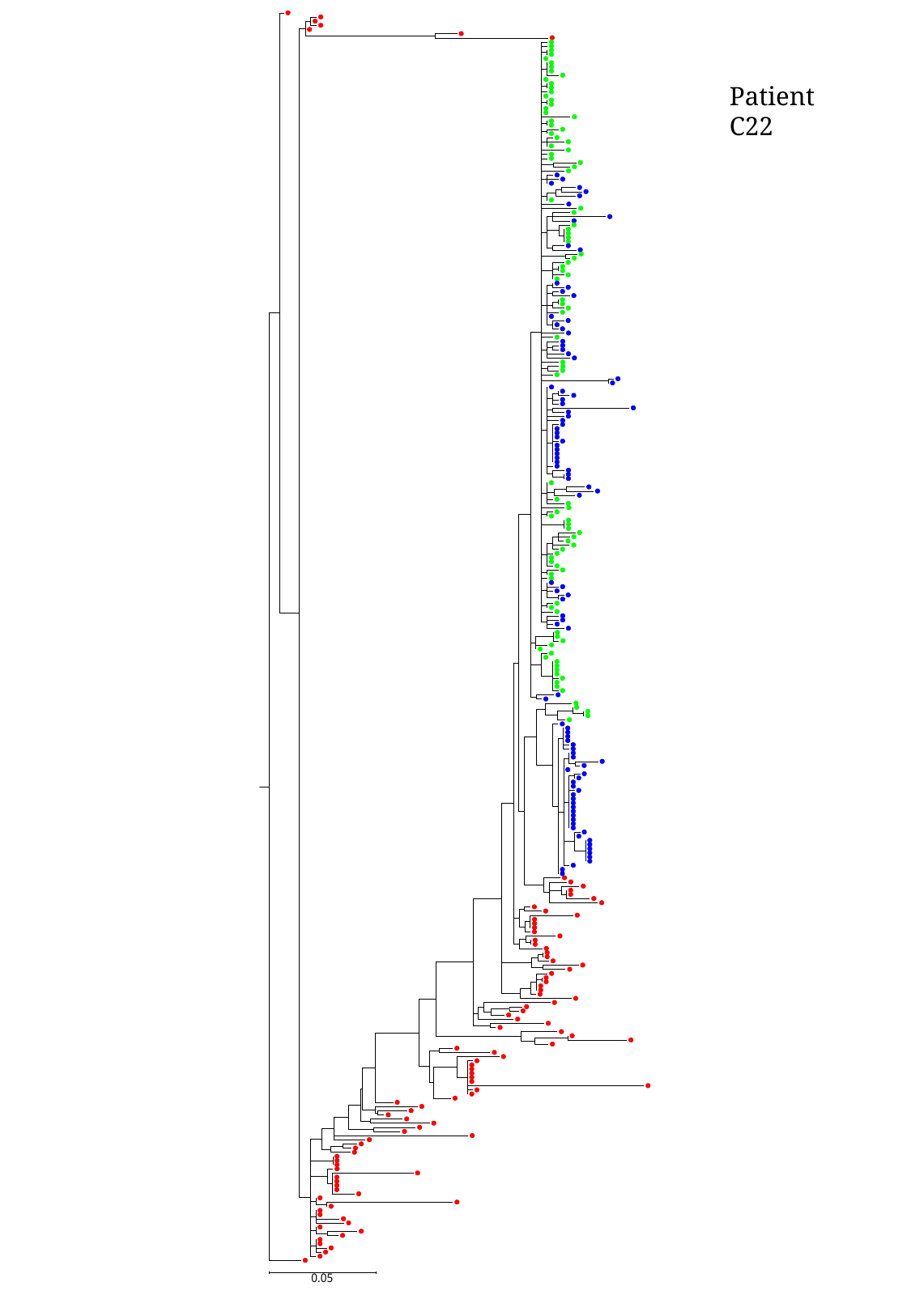

Patient C22

## Slide 6
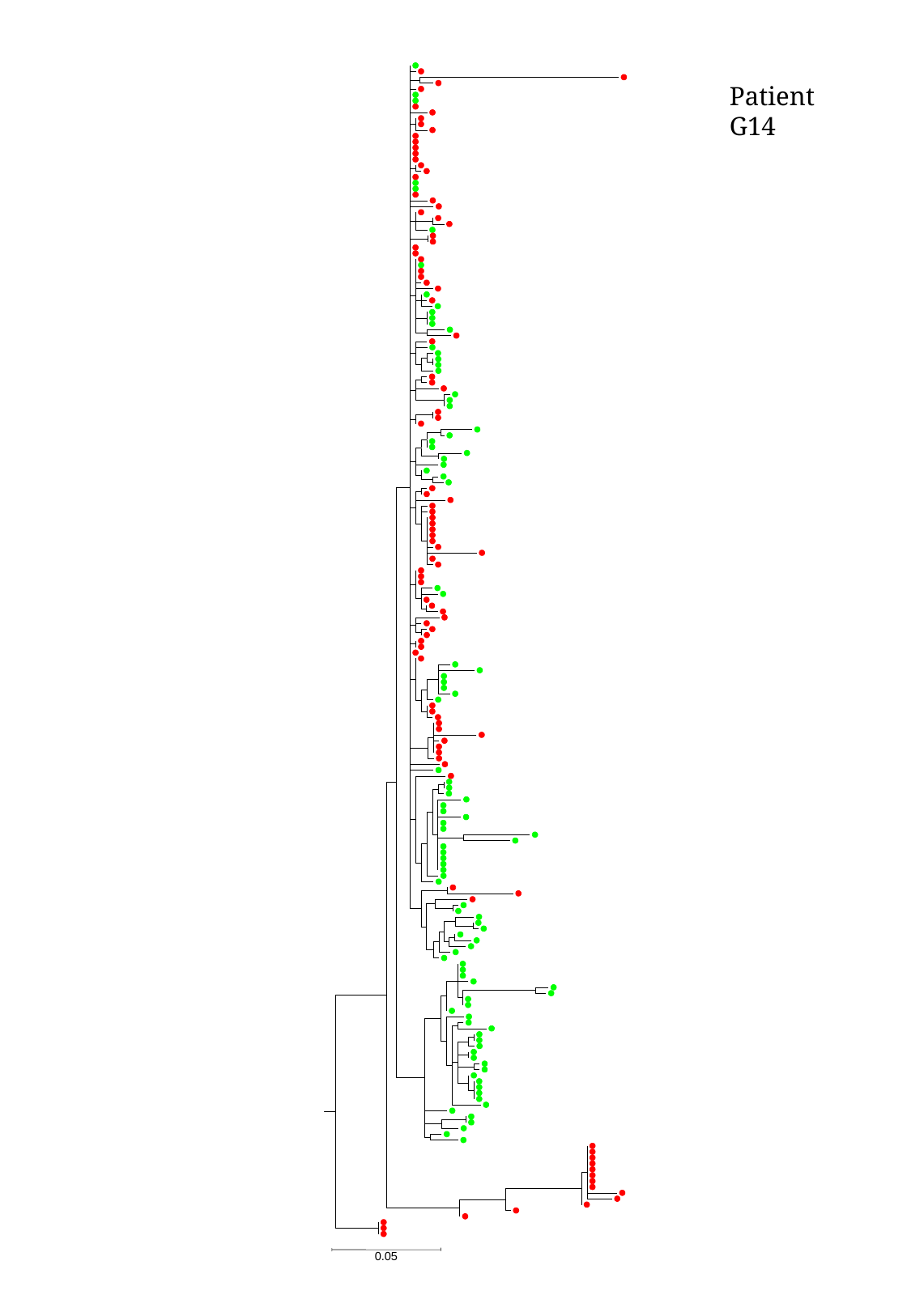

Patient G14
0.05

## Slide 7
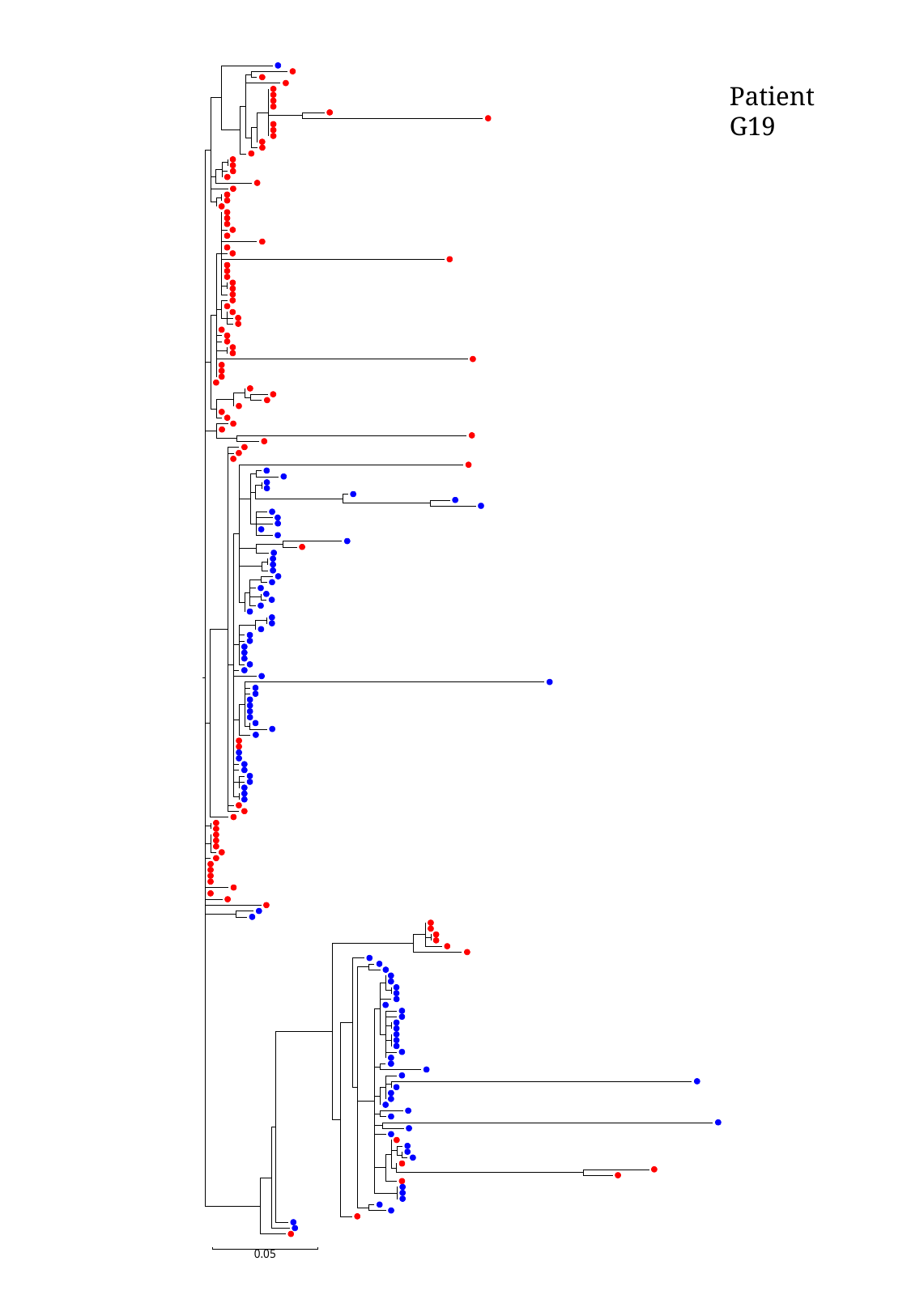

Patient G19

## Slide 8
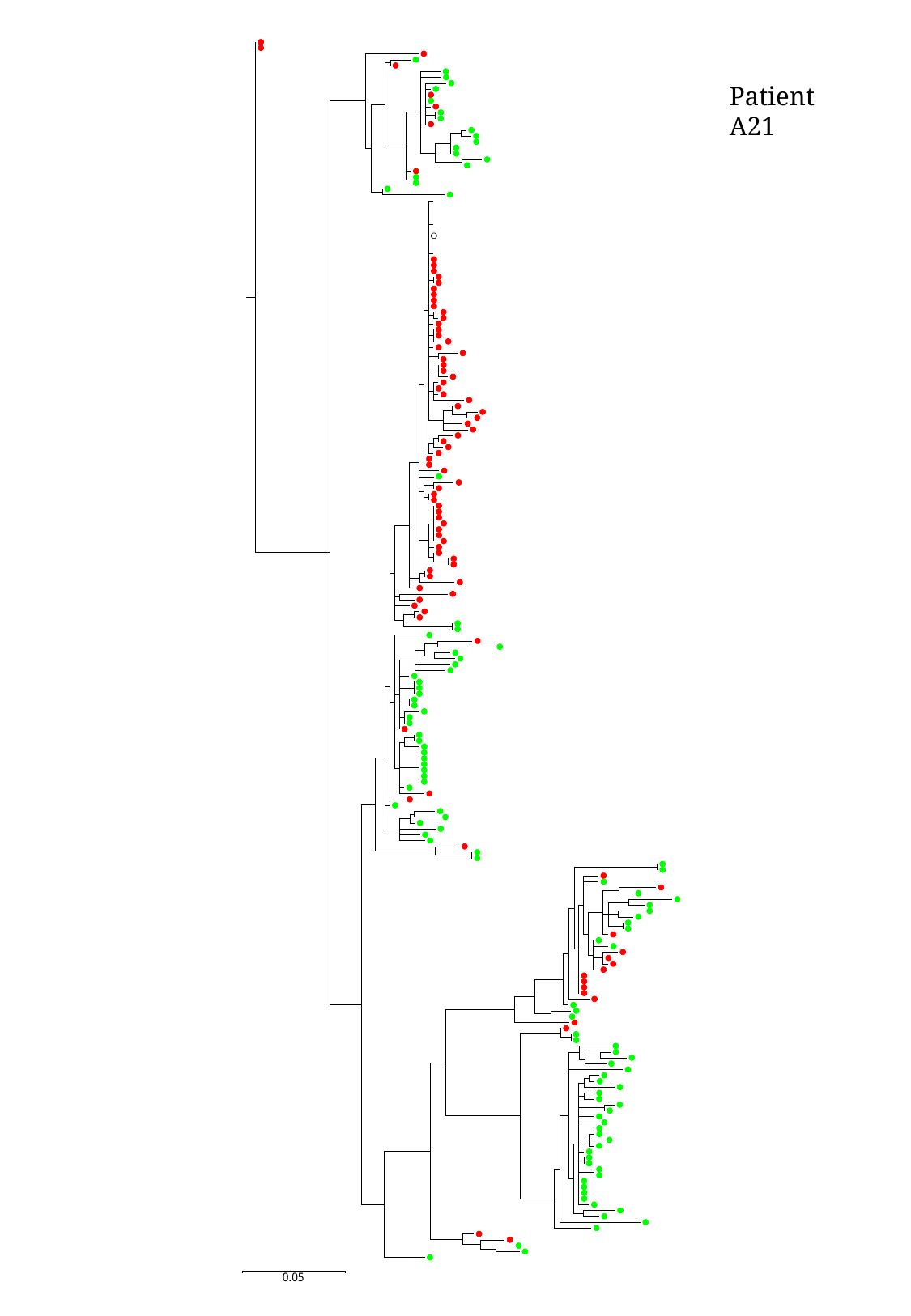

Patient A21

## Slide 9
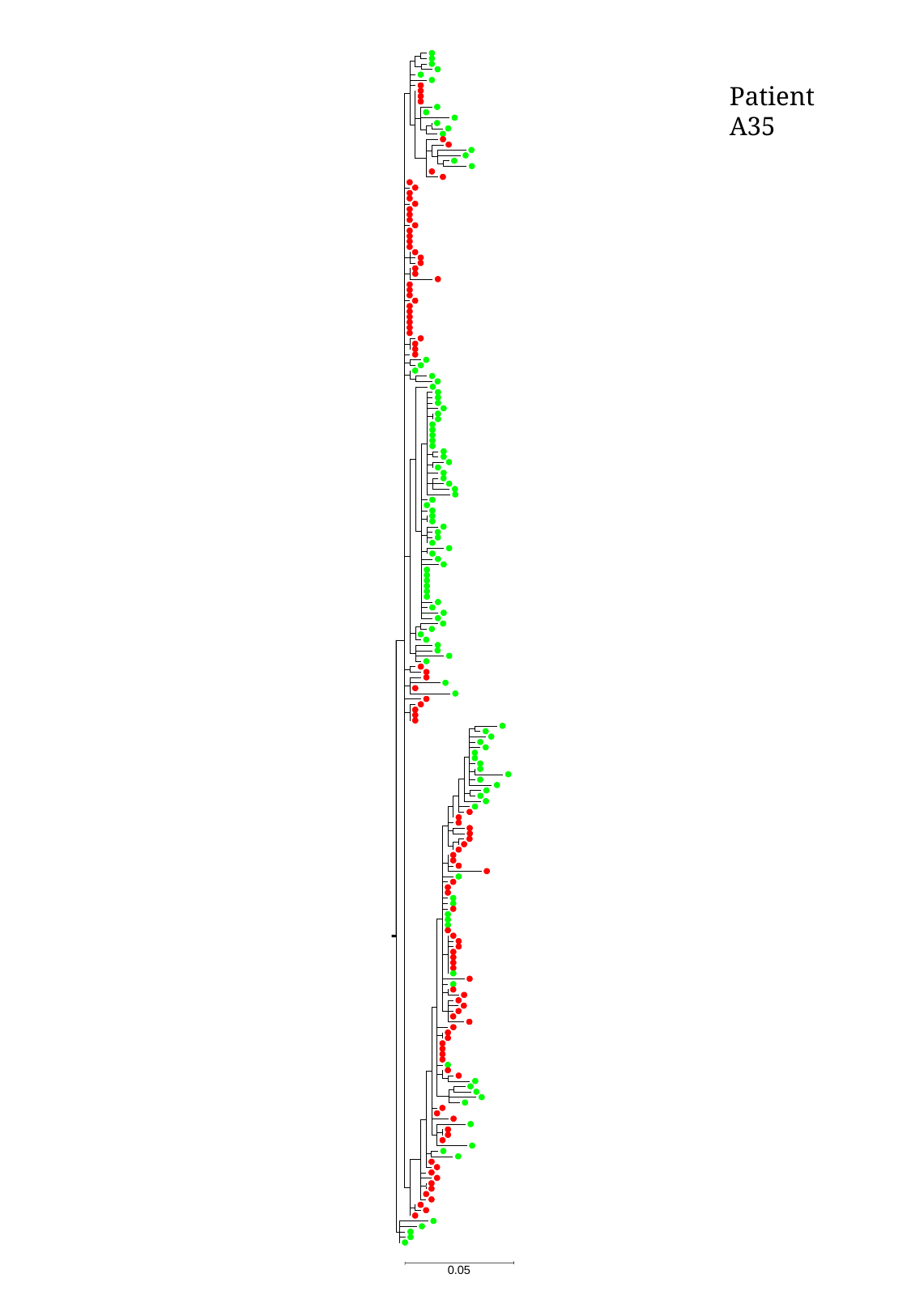

Patient A35
0.05

## Slide 10
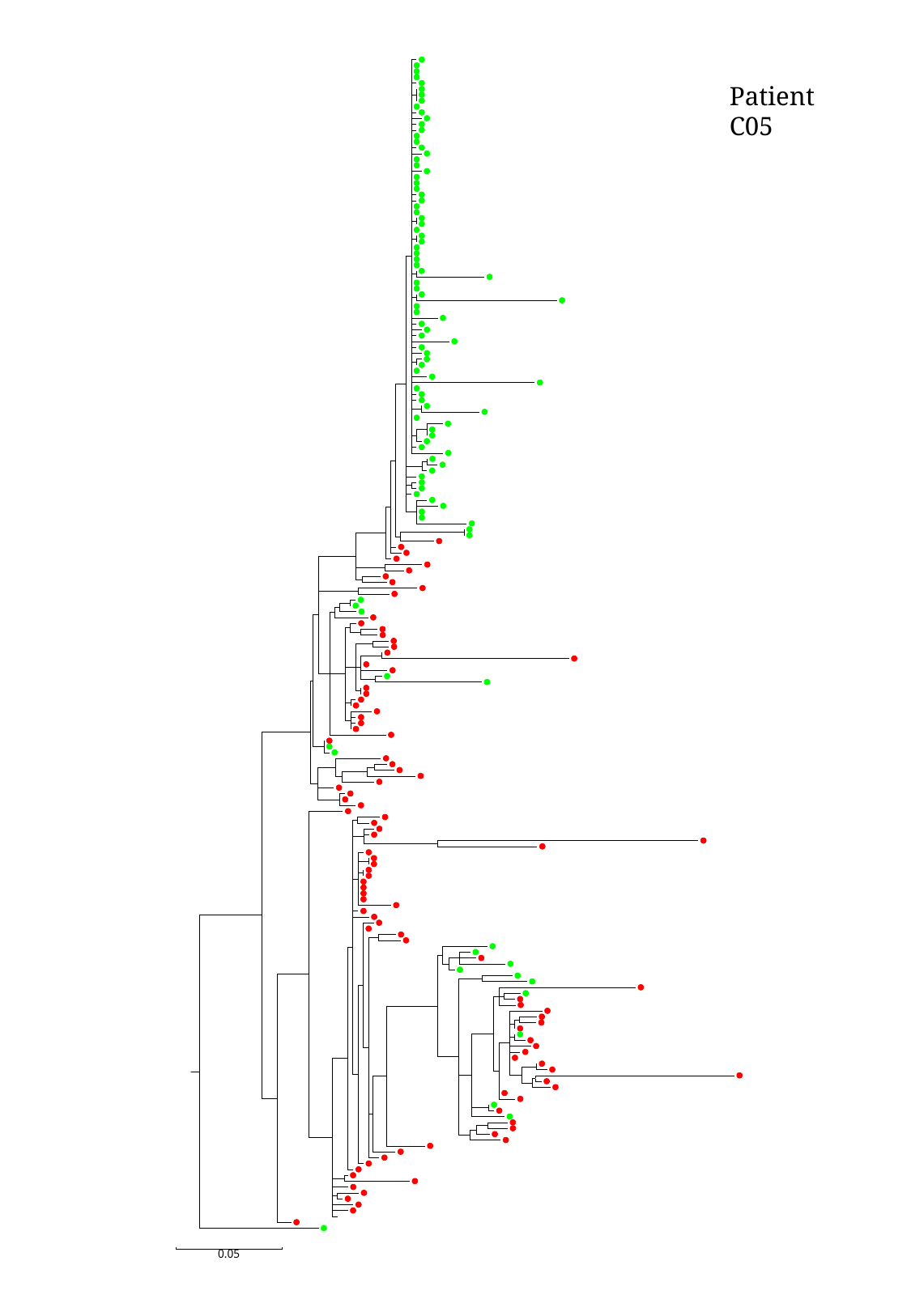

Patient C05

## Slide 11
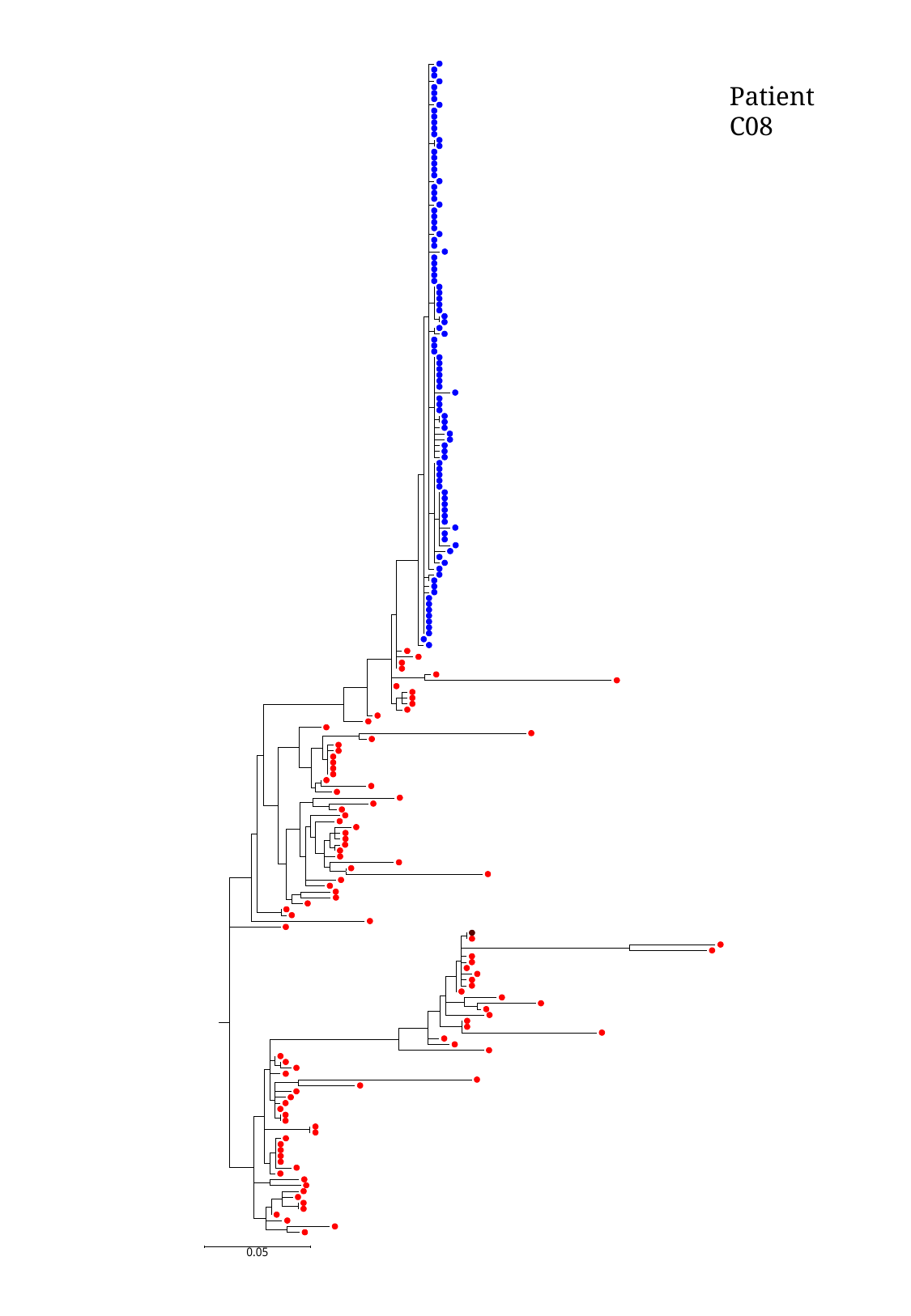

Patient C08

## Slide 12
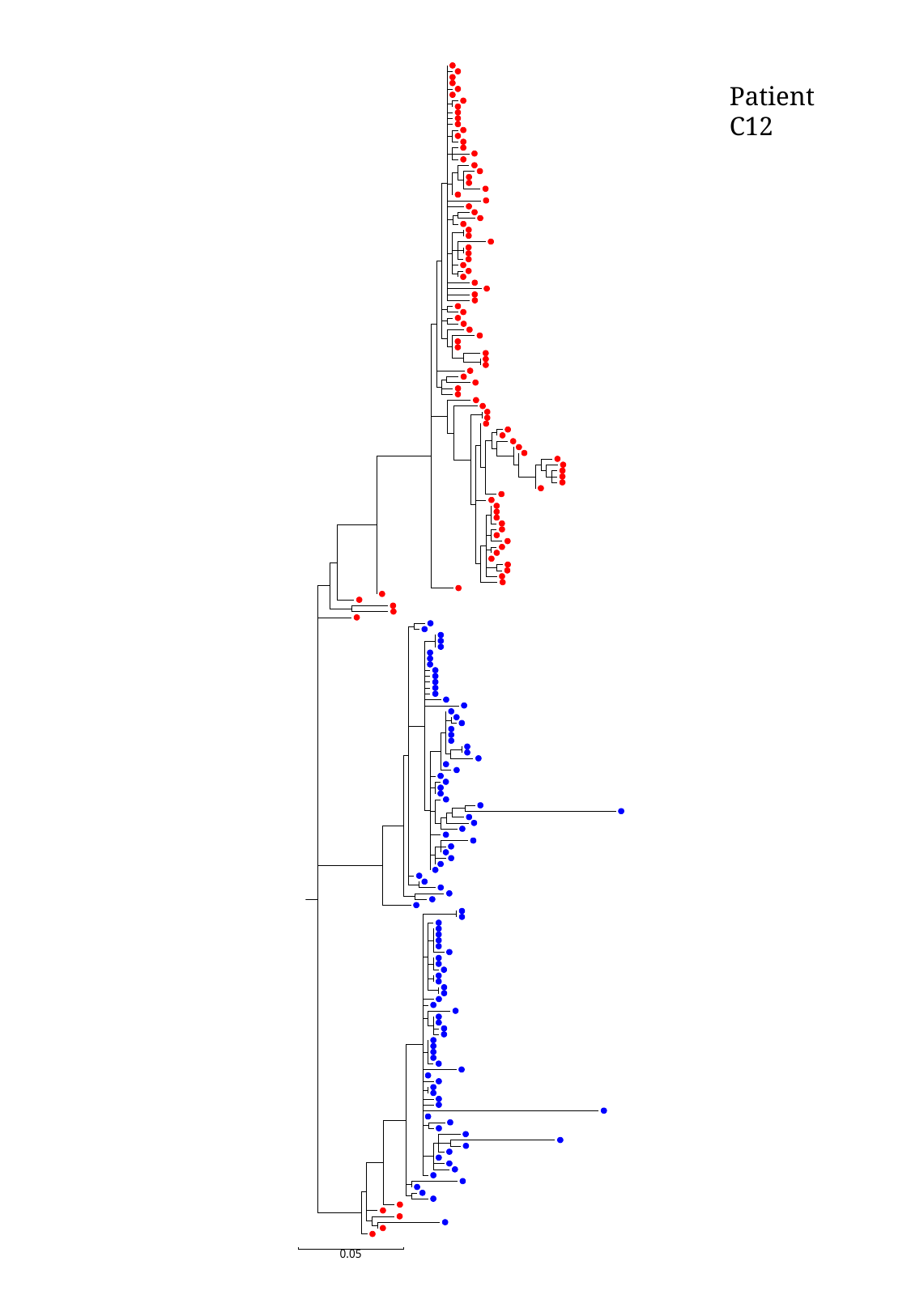

Patient C12

## Slide 13
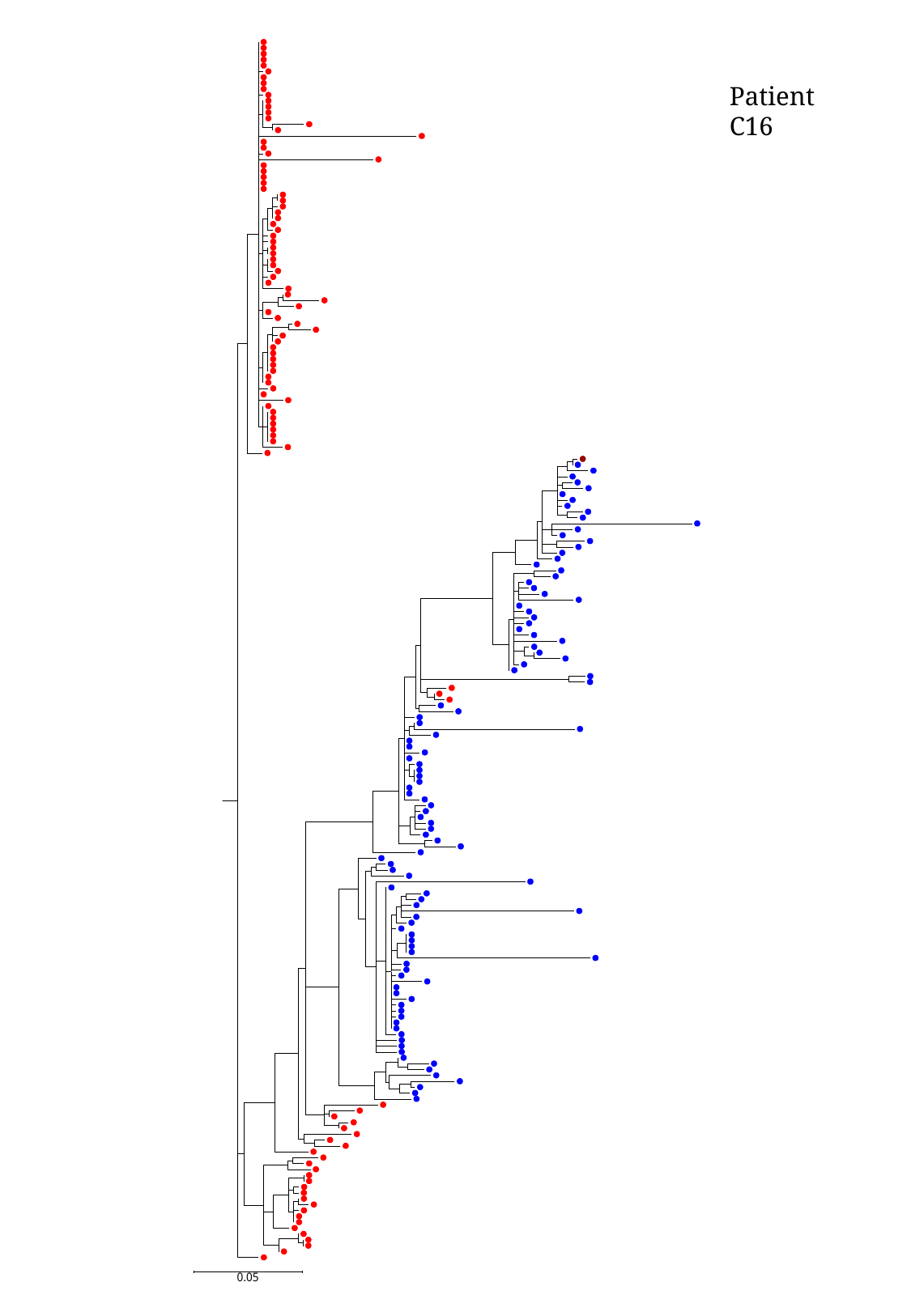

Patient C16

## Slide 14
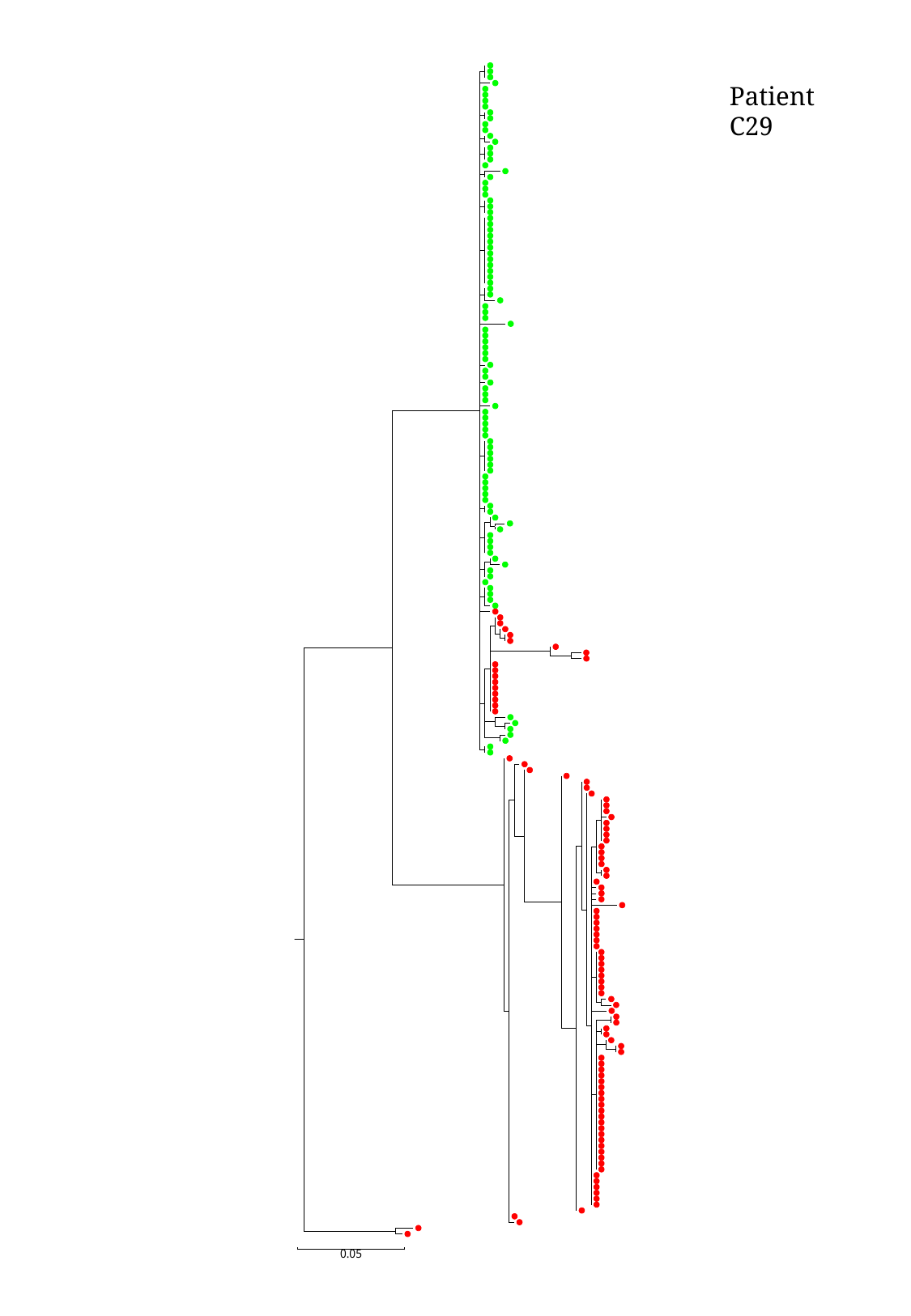

Patient C29

## Slide 15
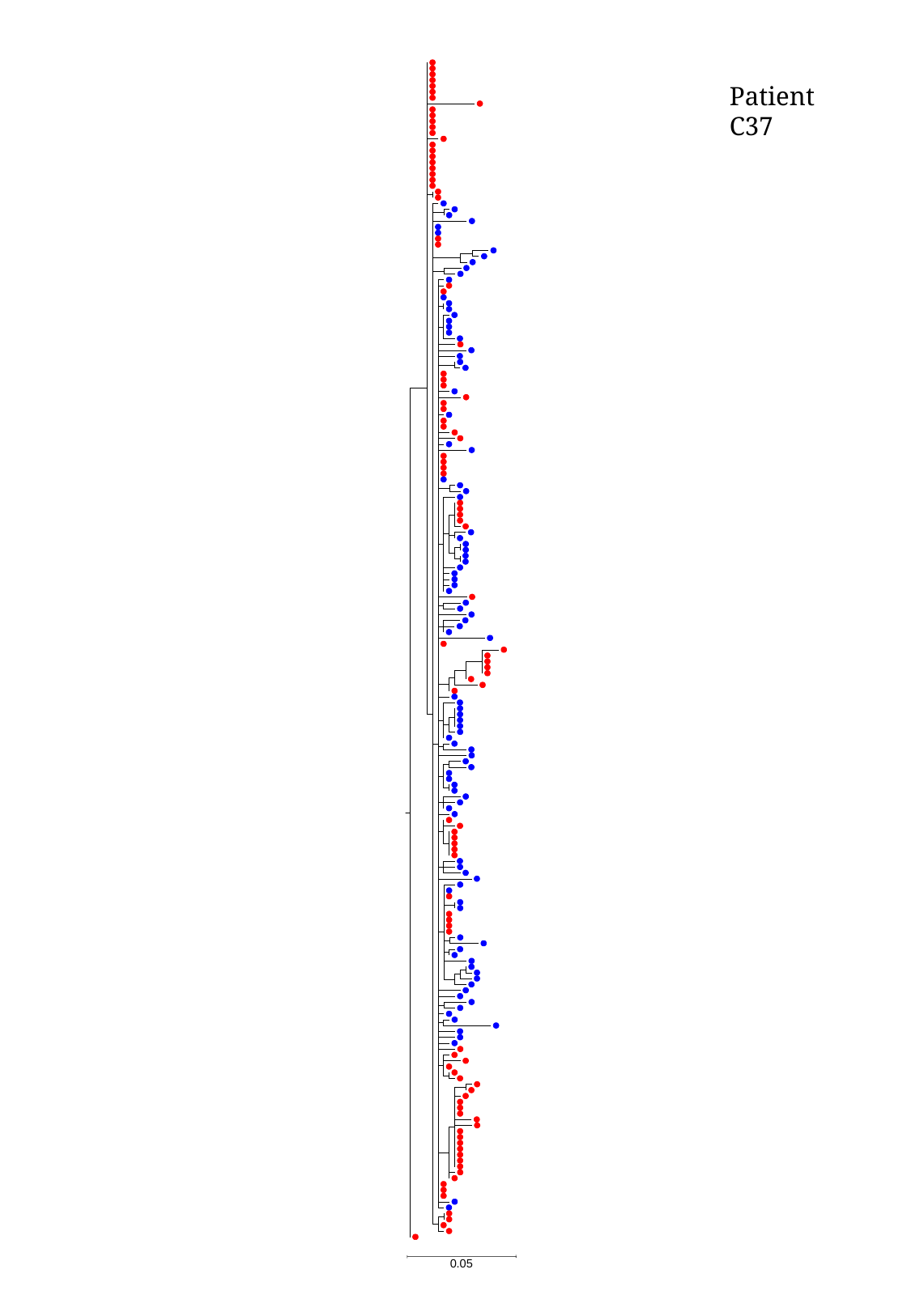

Patient C37
0.05

## Slide 16
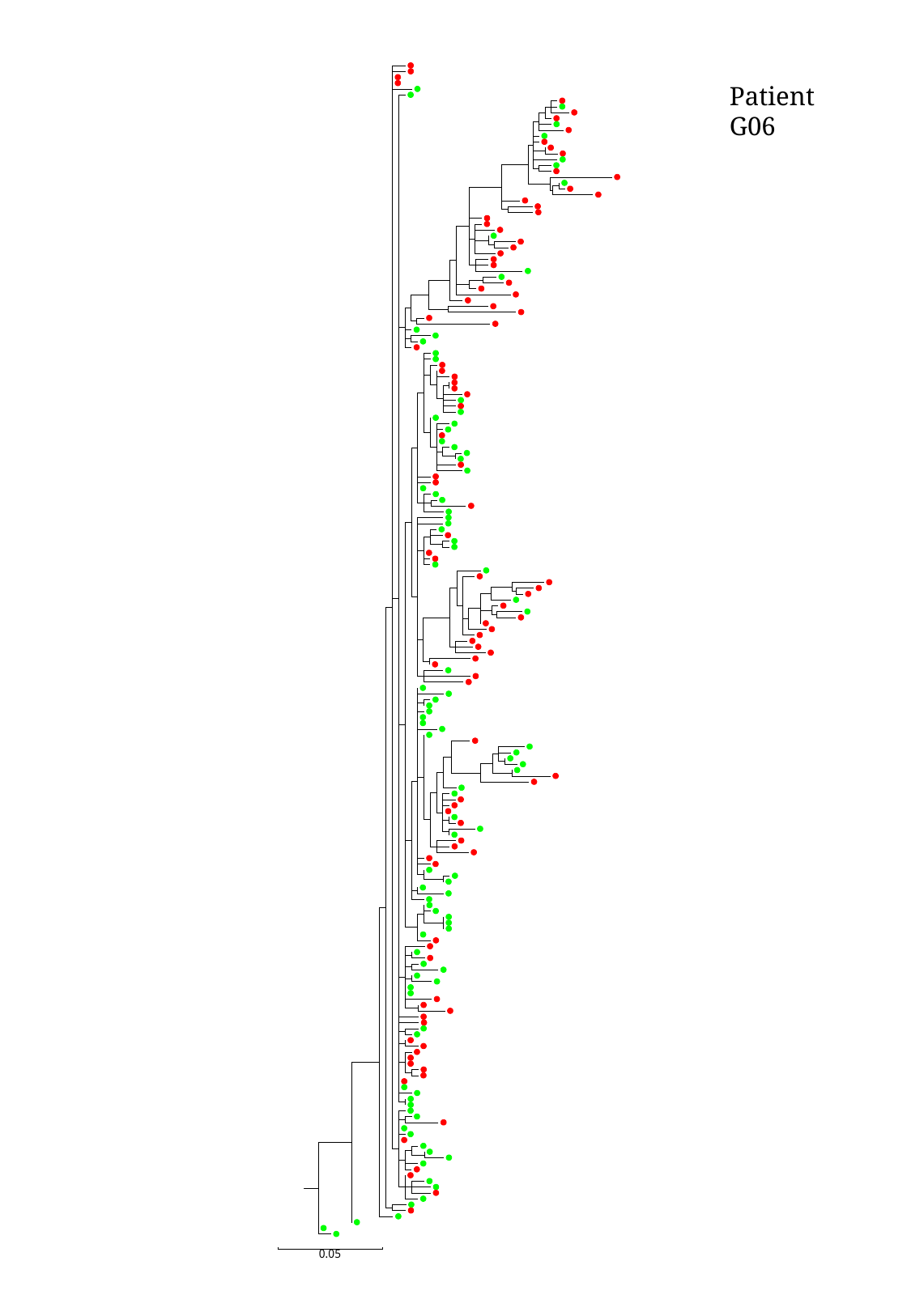

Patient G06

## Slide 17
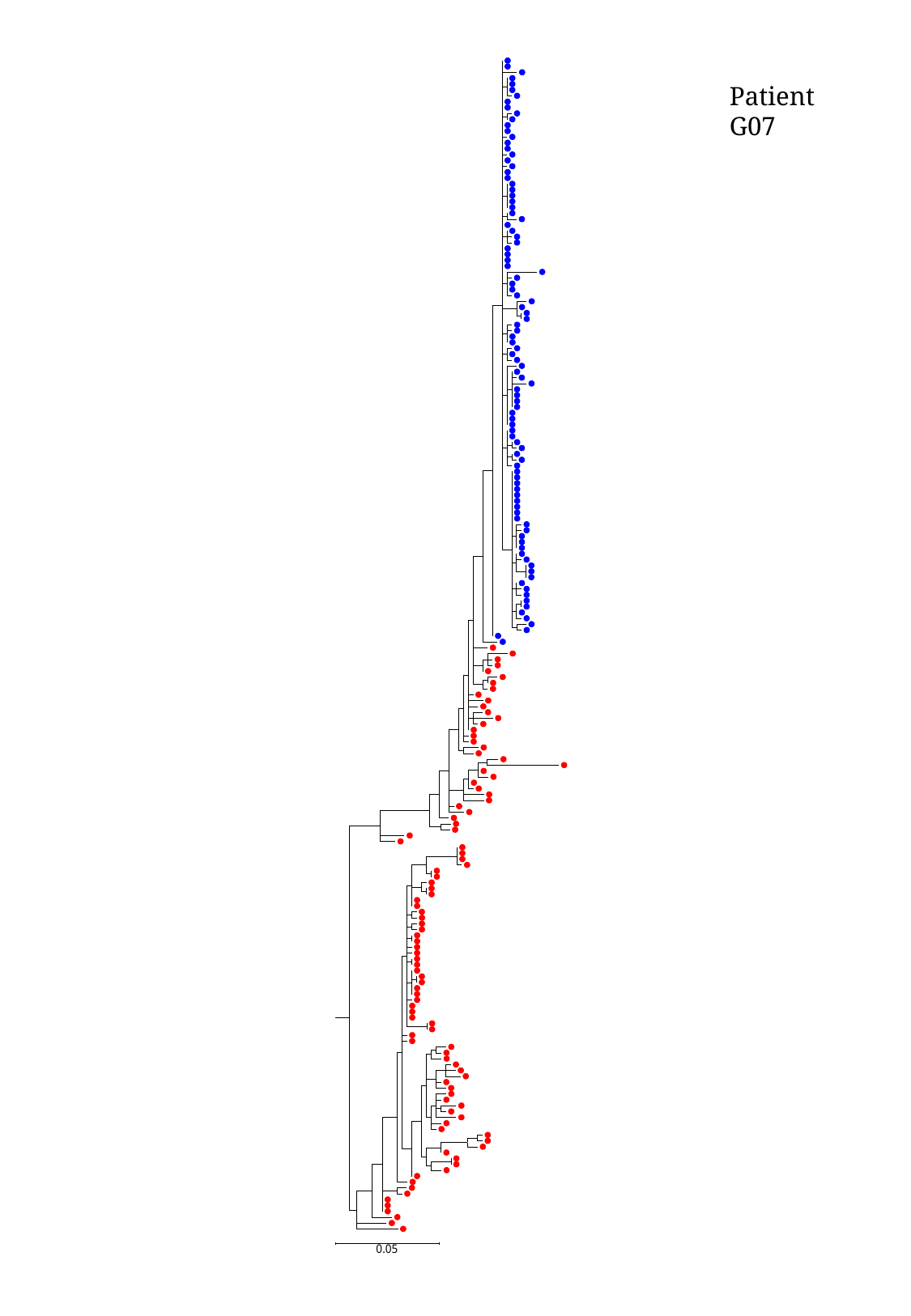

Patient G07

## Slide 18
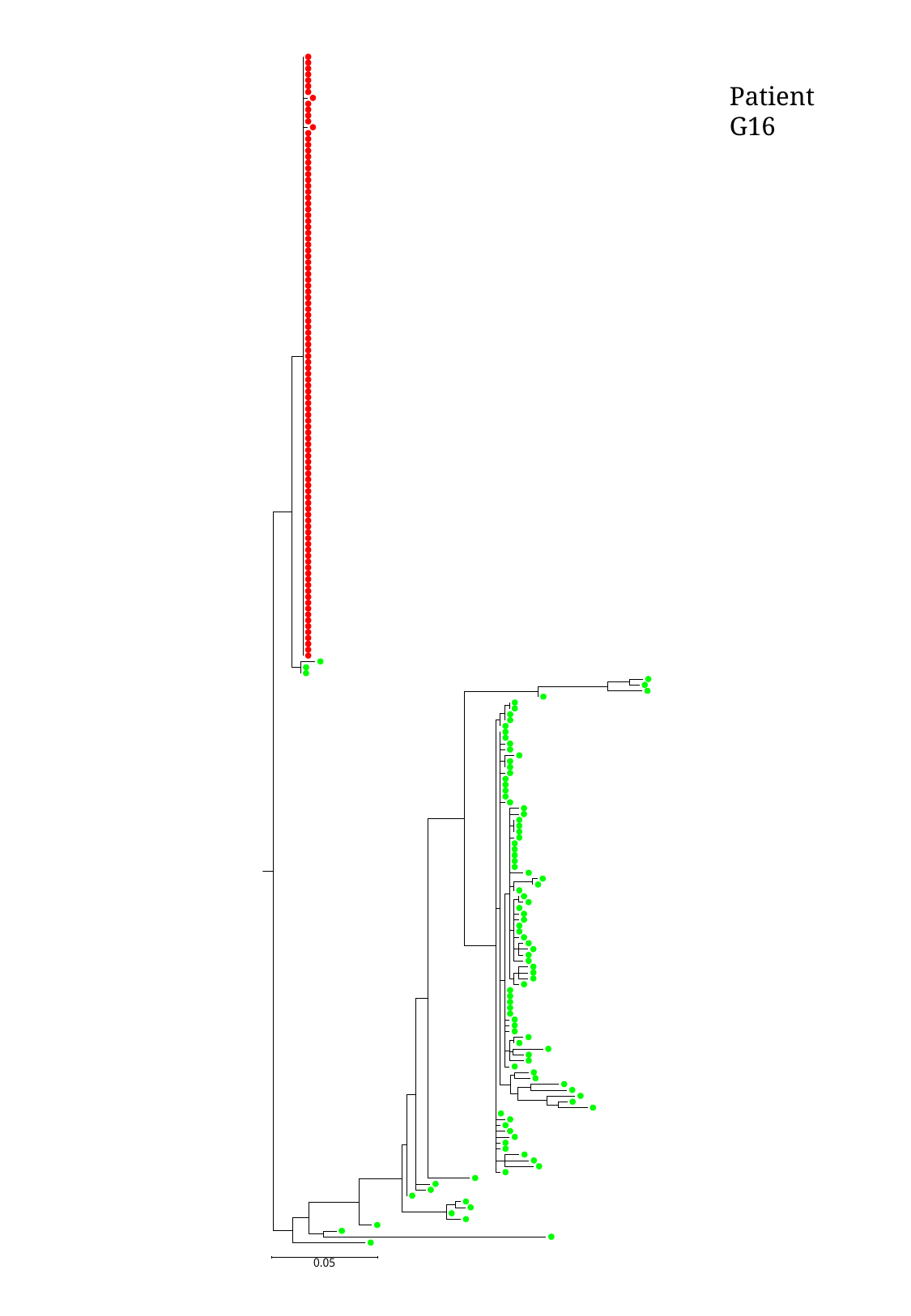

Patient G16

## Slide 19
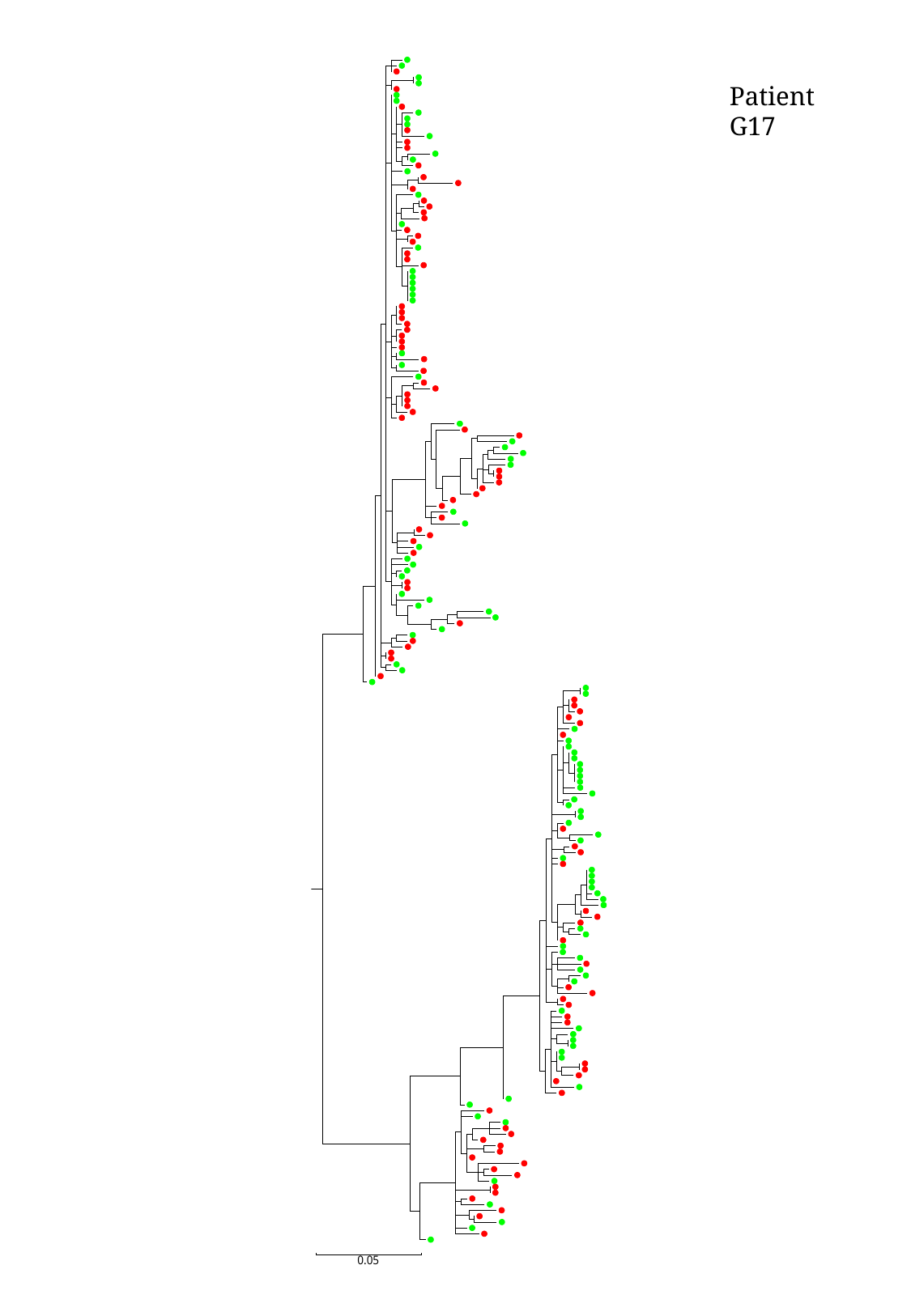

Patient G17

## Slide 20
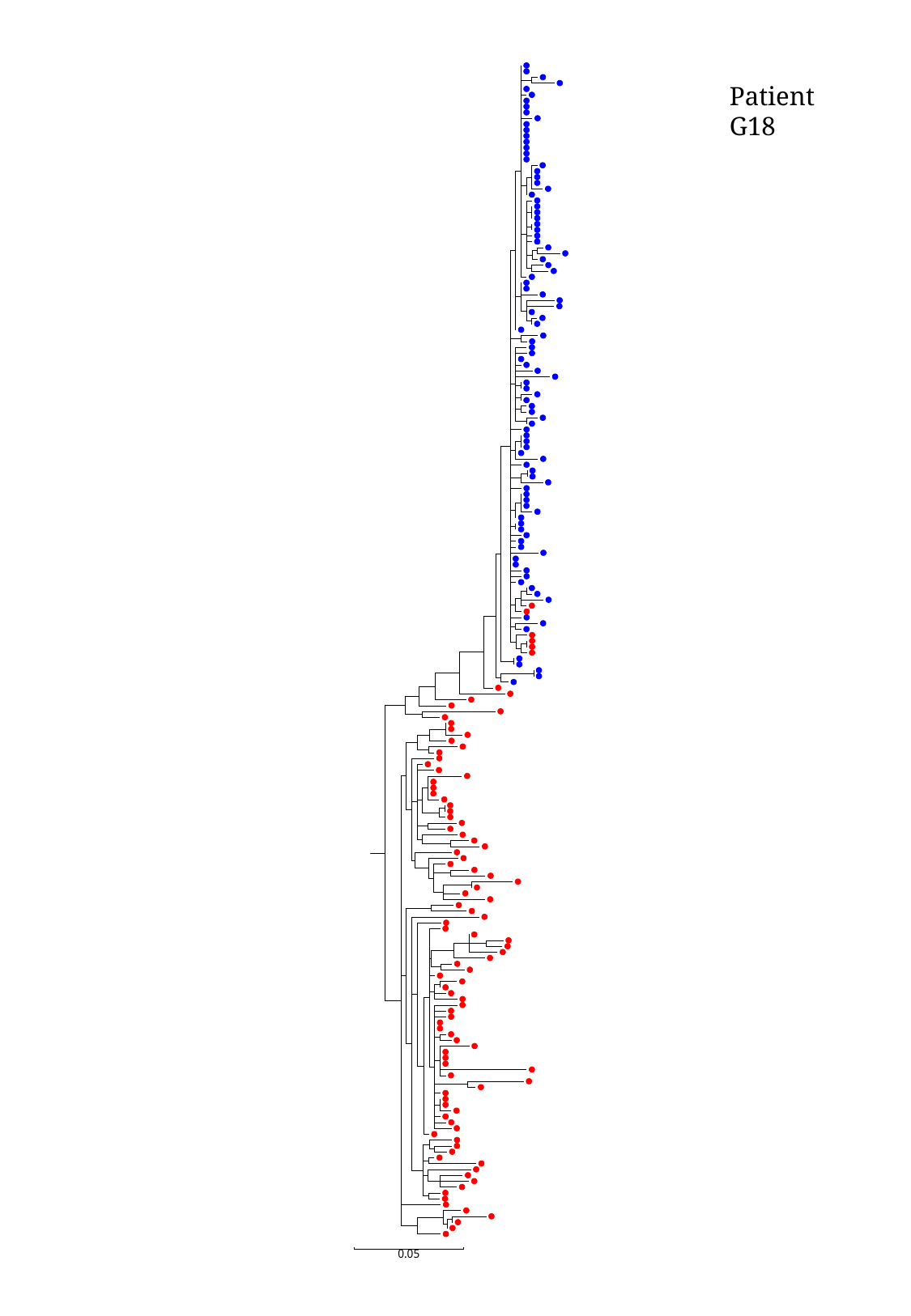

Patient G18

## Slide 21
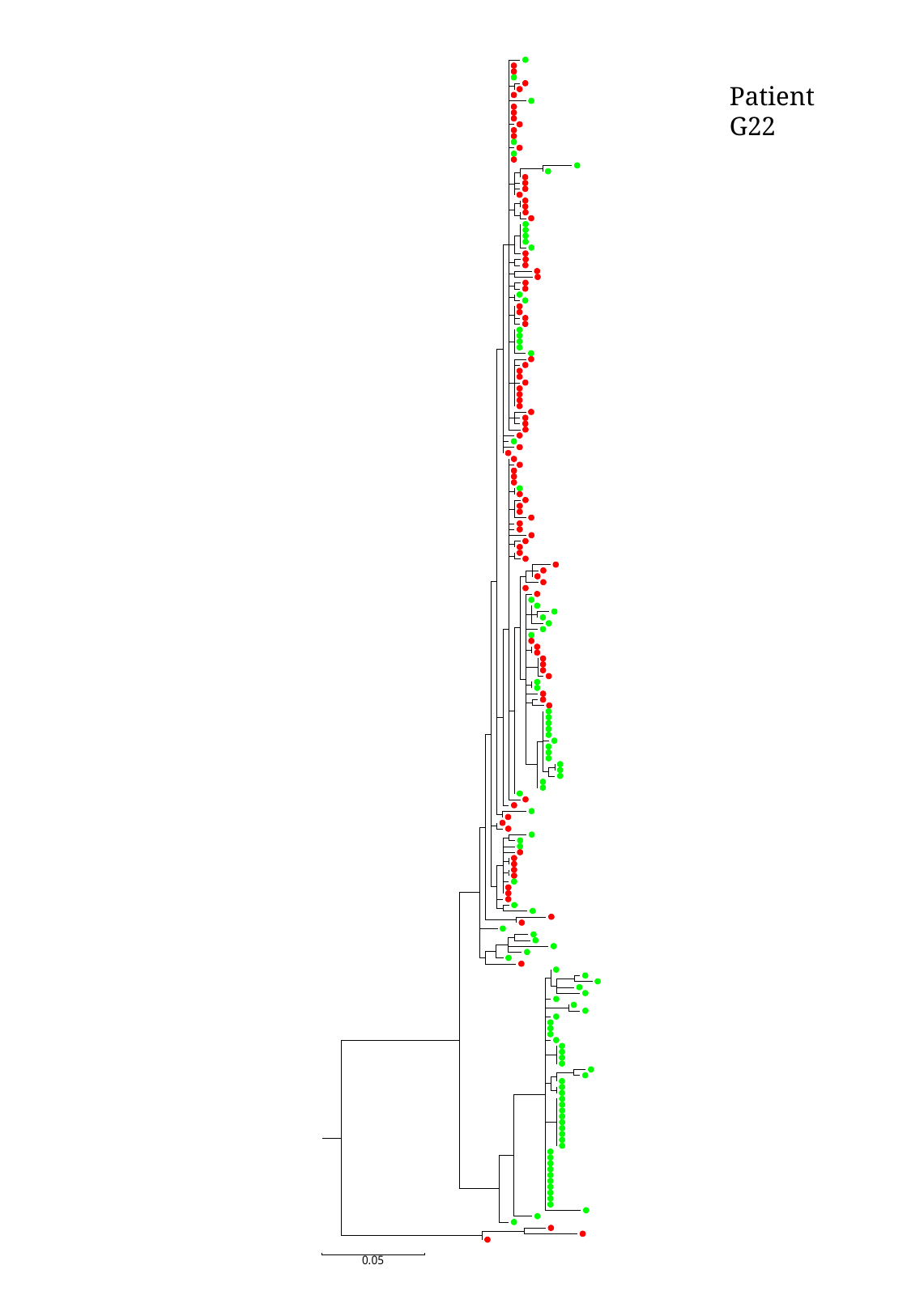

Patient G22

## Slide 22
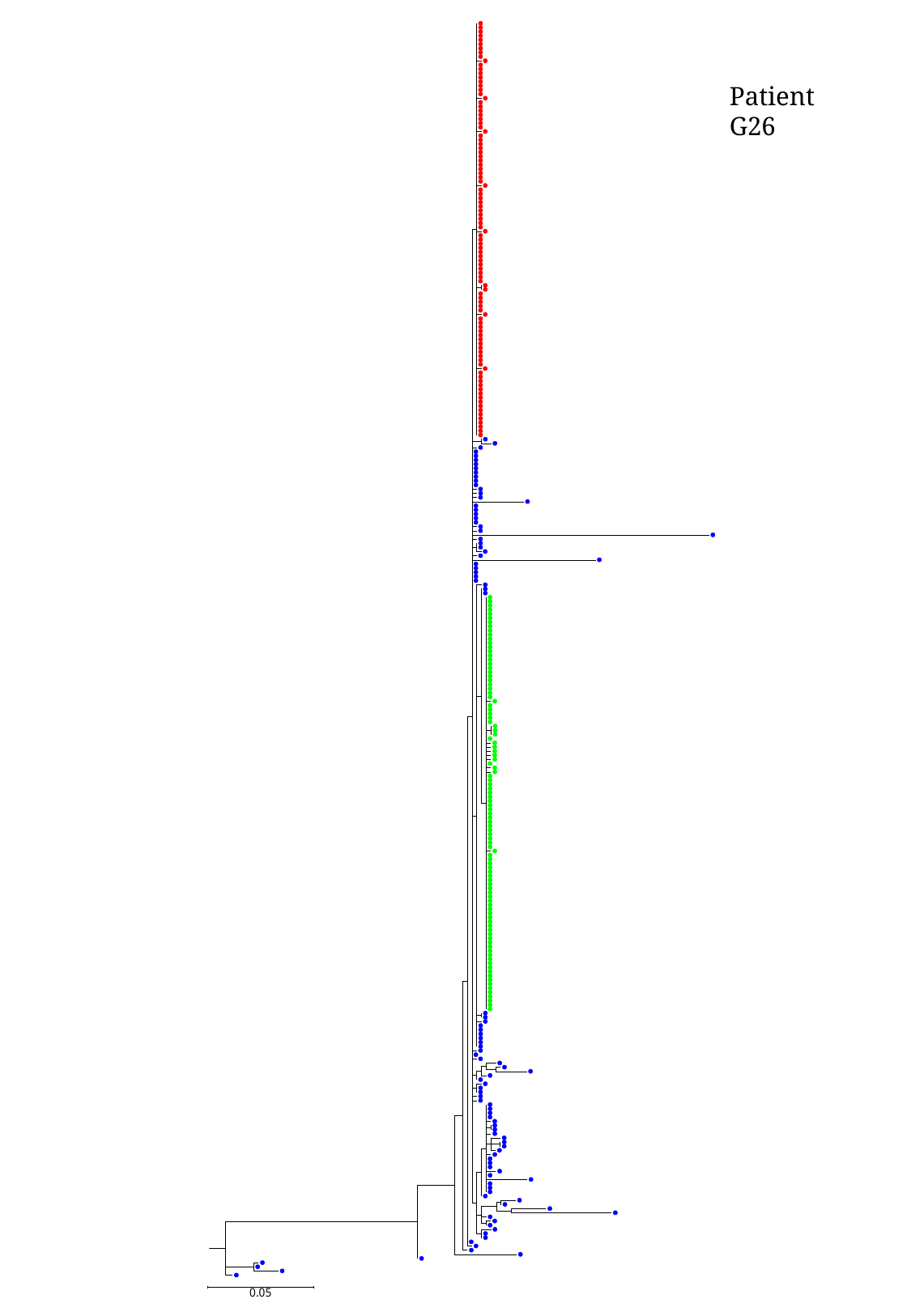

Patient G26
