# Supplementary material for: Genetic Variability of Hepatitis C Virus before and after Combined Therapy of Interferon plus Ribavirin
Source: PLoS One. 2008 Aug 26;3(8):e3058. doi: 10.1371/journal.pone.0003058 (PMC2518109; doi:10.1371/journal.pone.0003058)
Supplement: Figure S2 — Phylogenetic trees for the NS5A region Phylogenetic trees for the NS5A region from all 22 analyzed patients. Different symbols are used to denote sequences sampled at T0 (red dots), T1 (green dots) and T2 (blue dots). (0.37 MB PPT) [file pone.0003058.s002.ppt]

## Slide 1
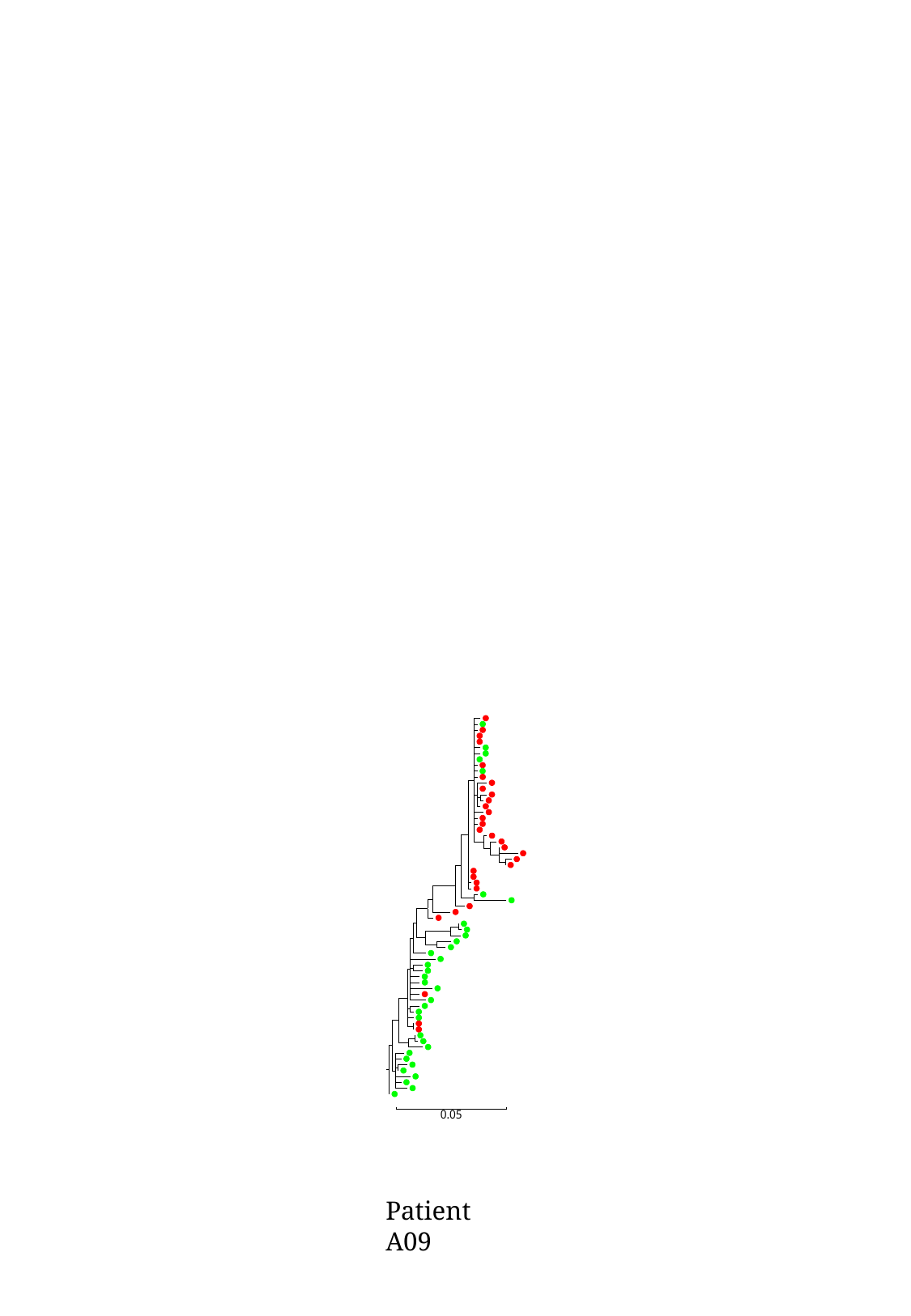

Patient A09

## Slide 2
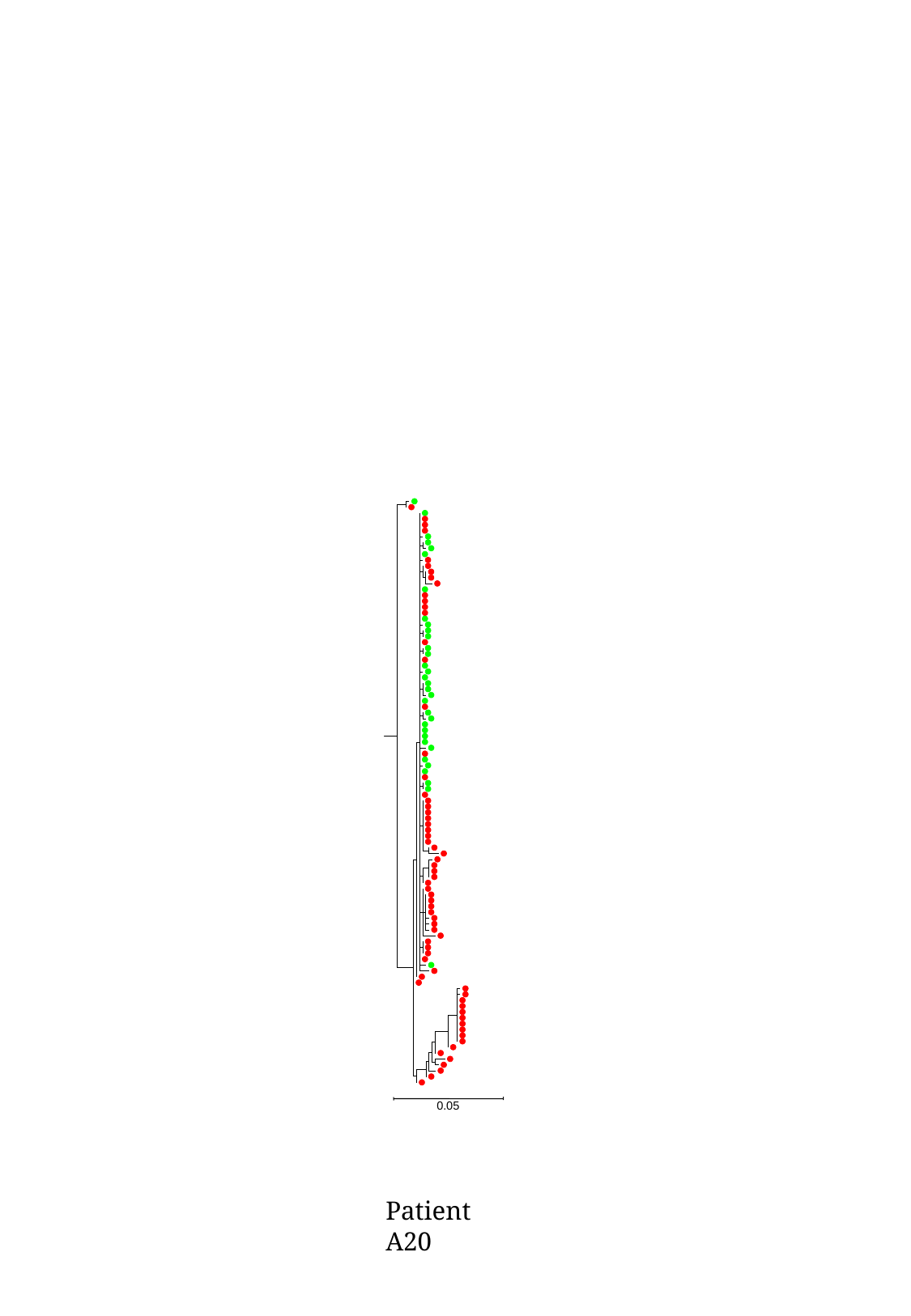

0.05
Patient A20

## Slide 3
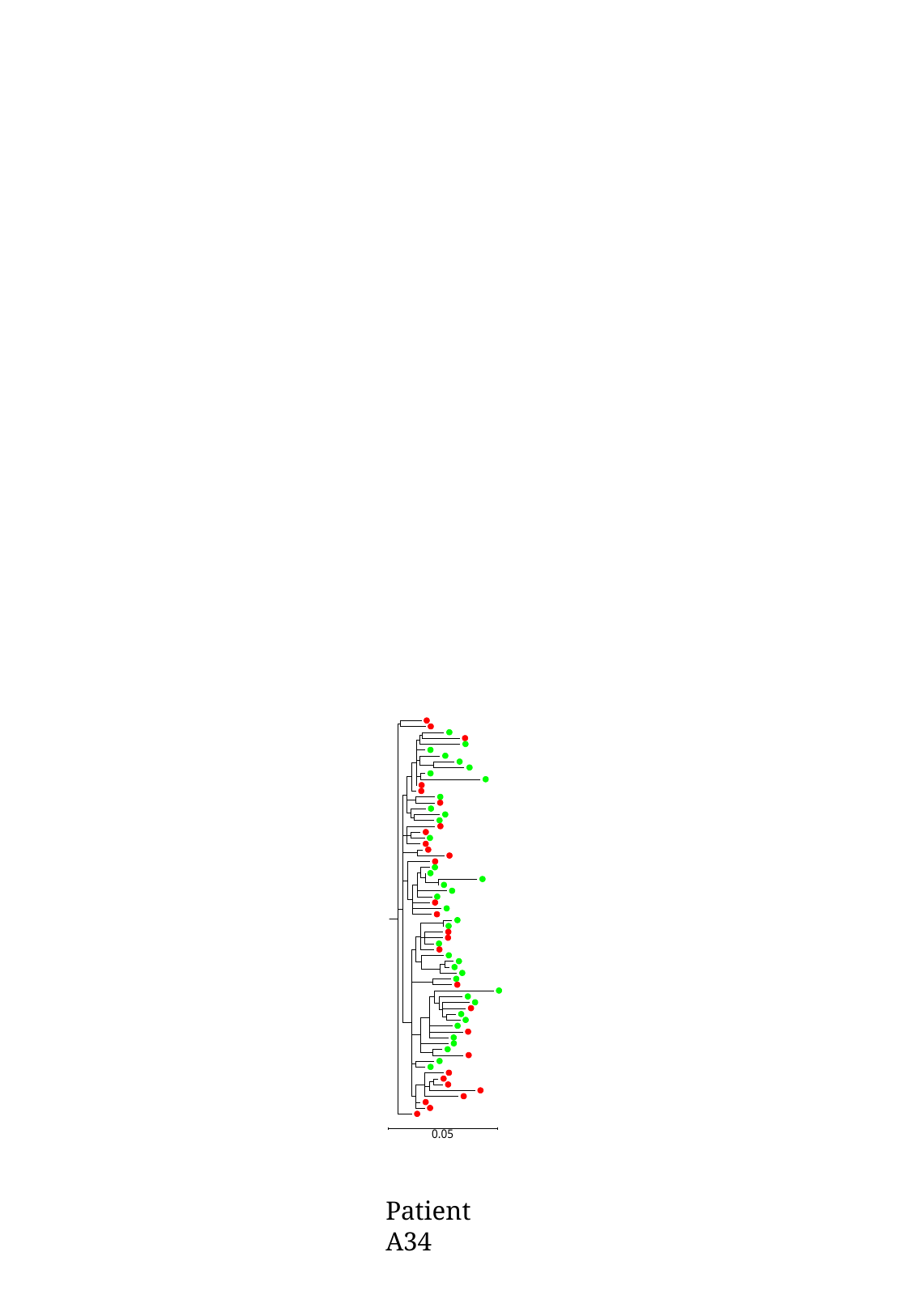

Patient A34

## Slide 4
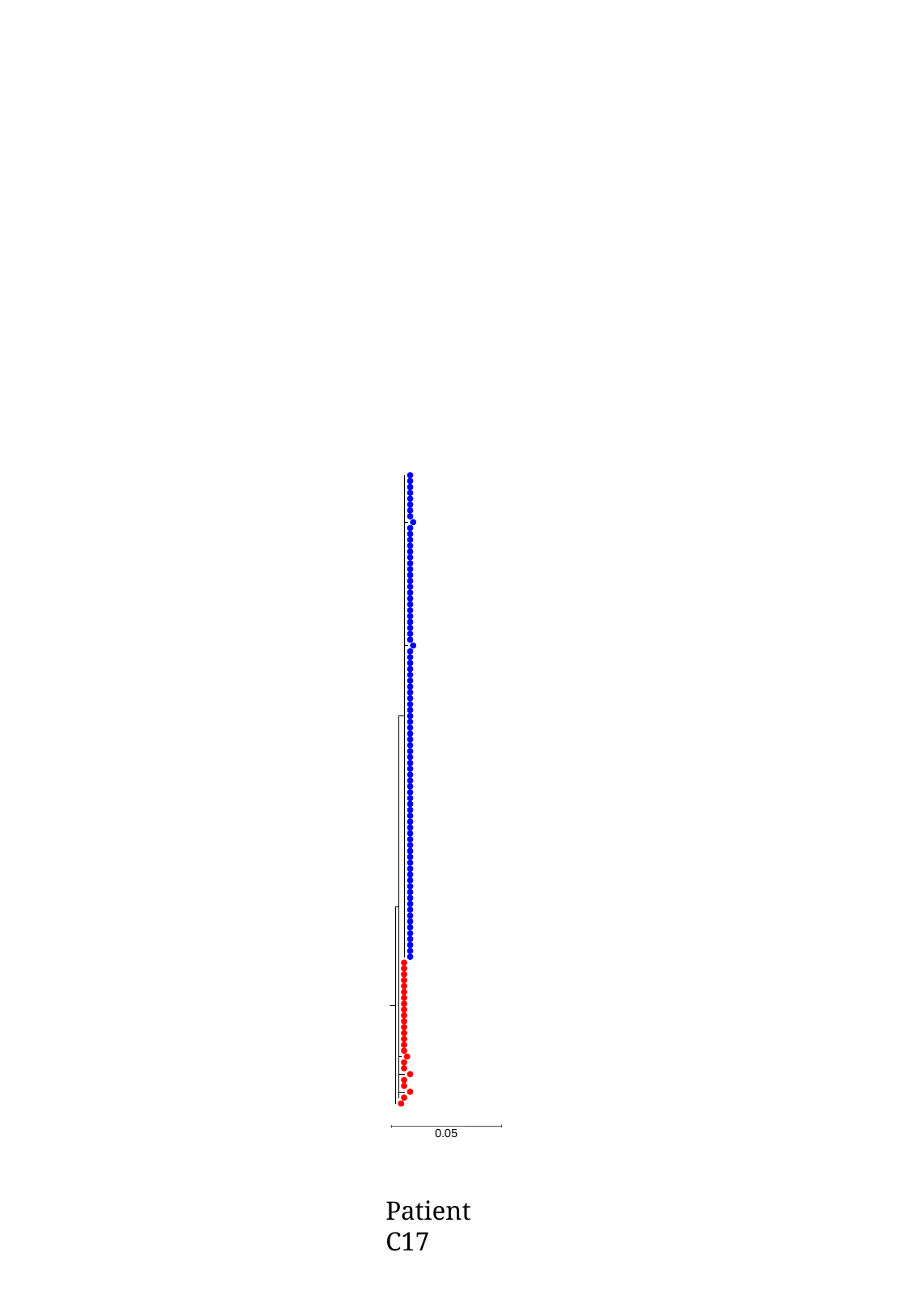

0.05
Patient C17

## Slide 5
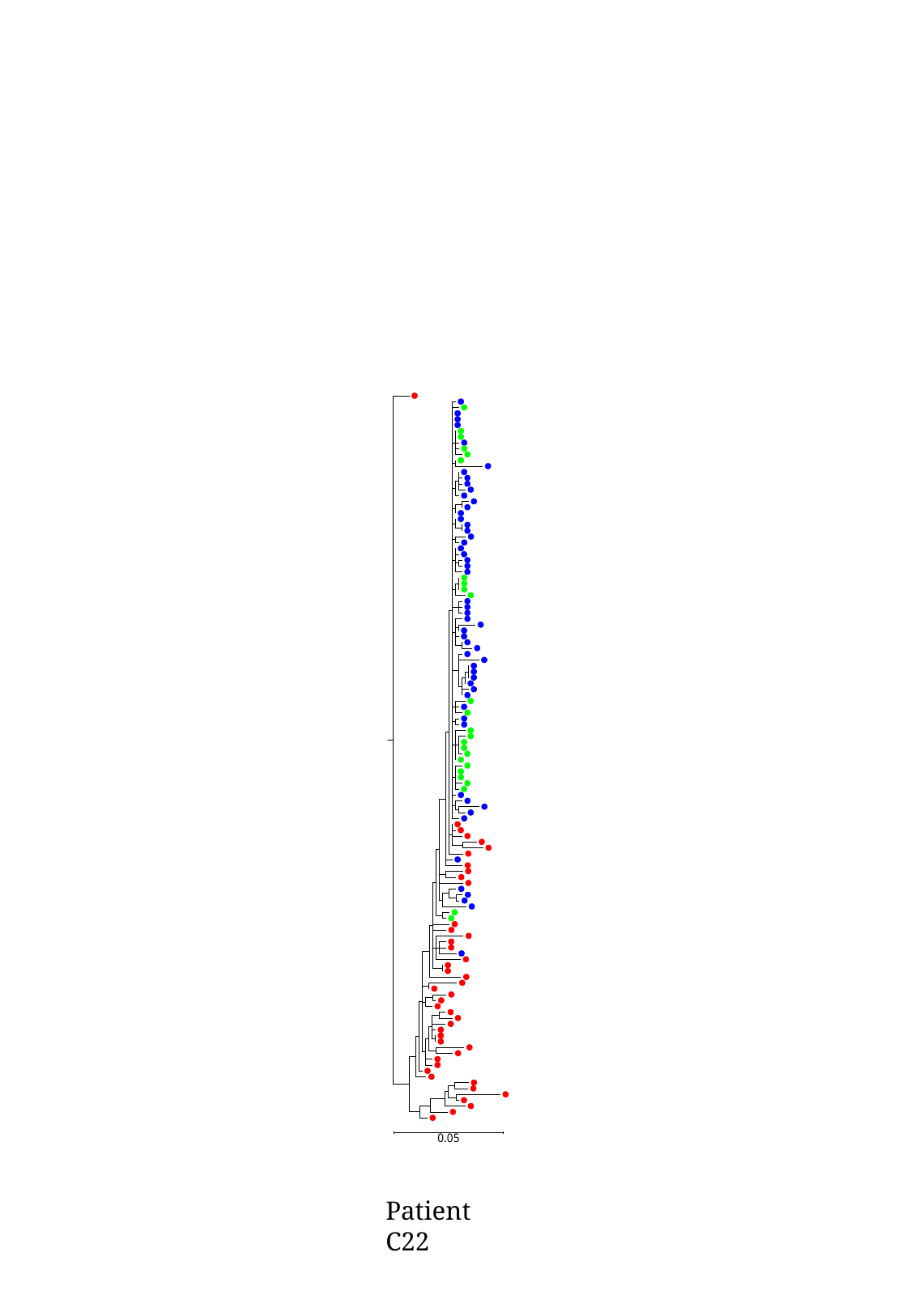

Patient C22

## Slide 6
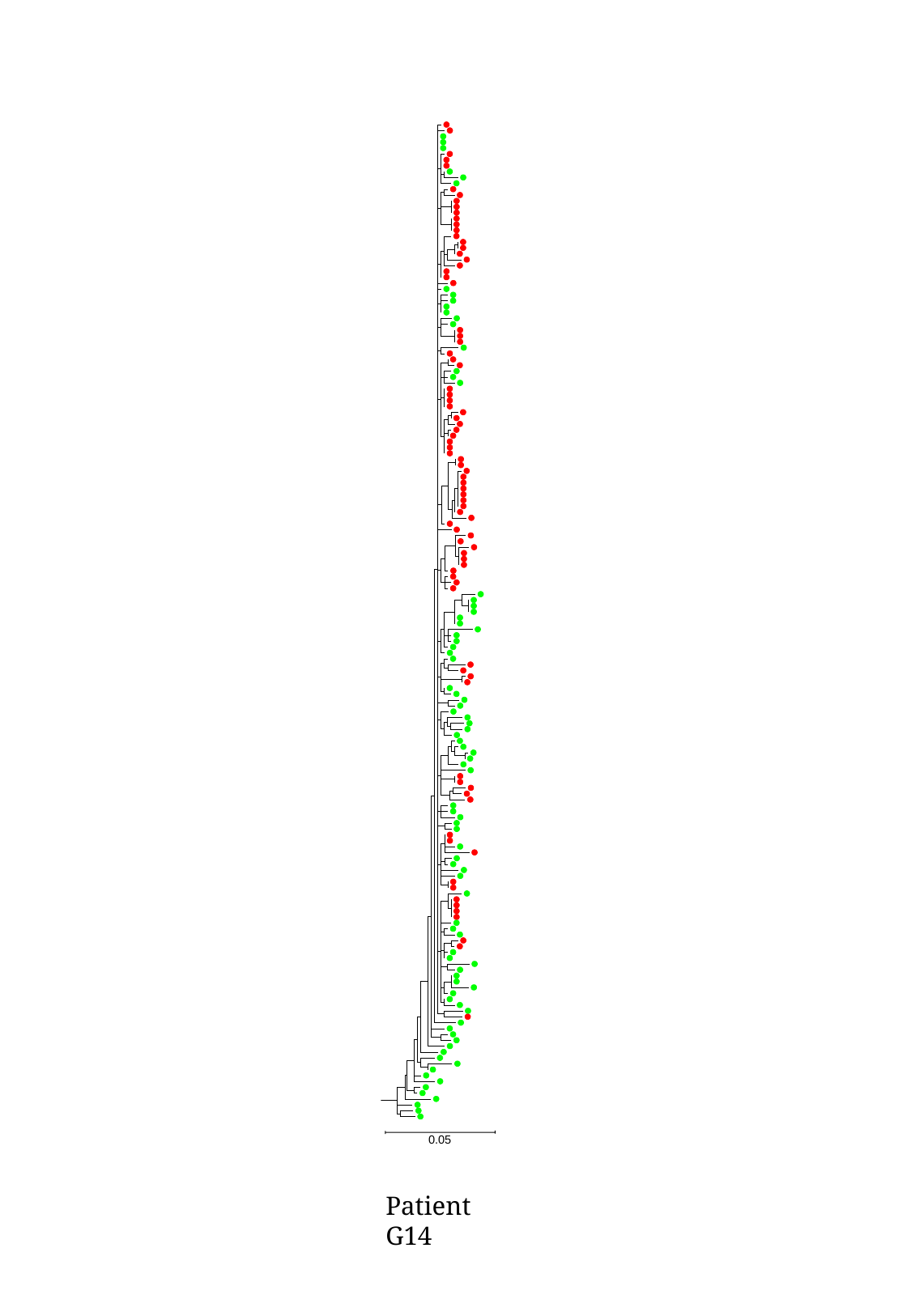

0.05
Patient G14

## Slide 7
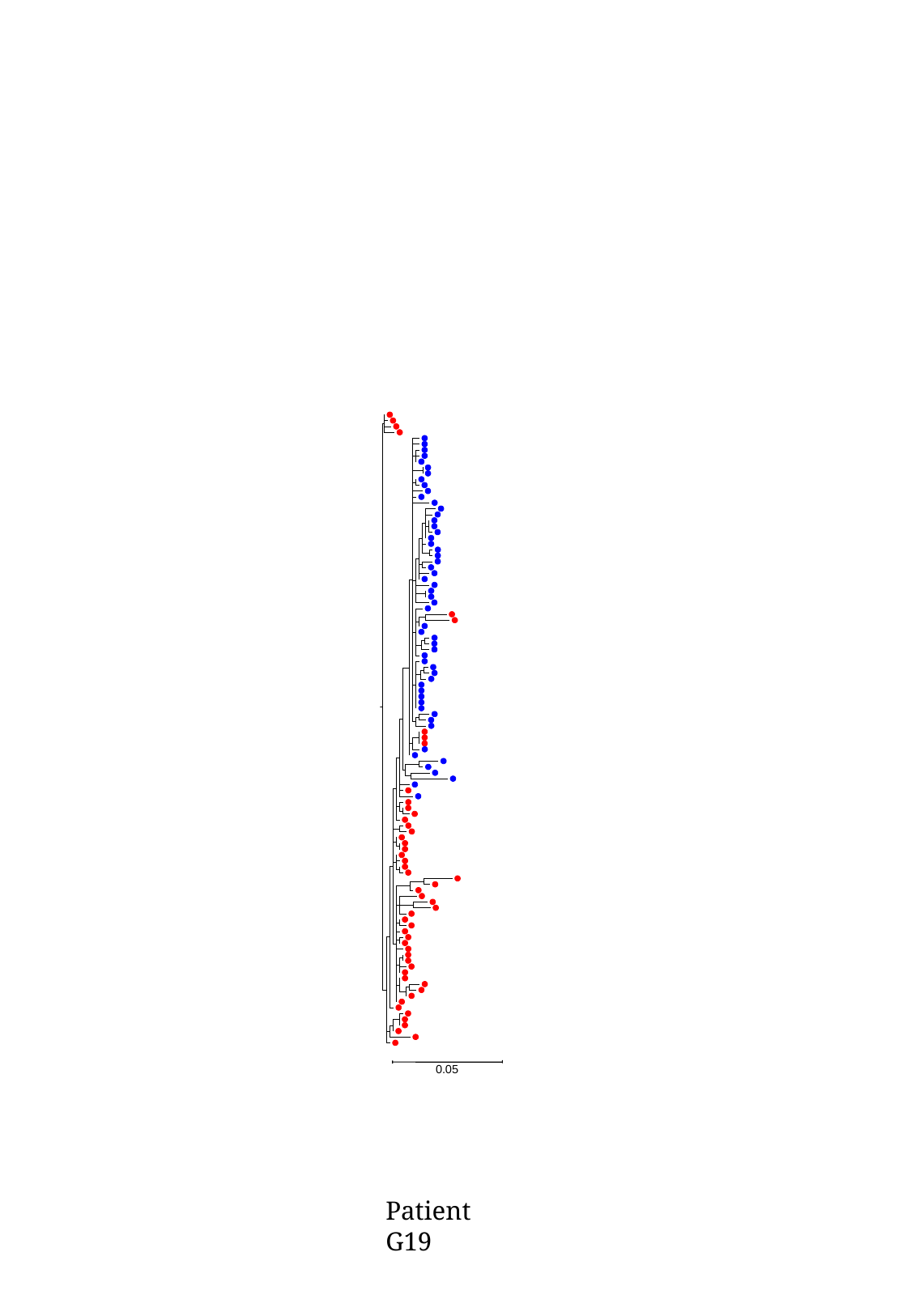

0.05
Patient G19

## Slide 8
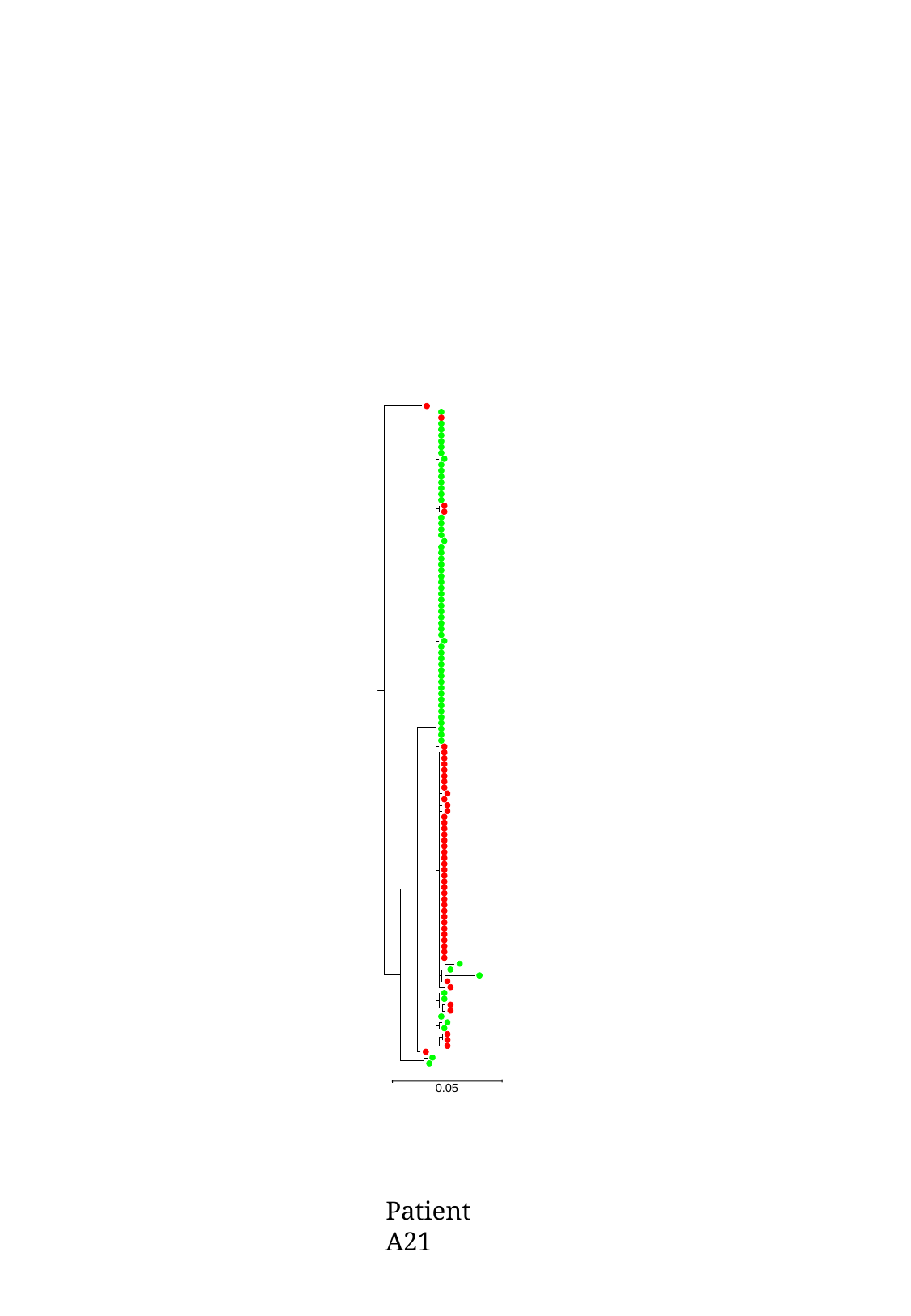

0.05
Patient A21

## Slide 9
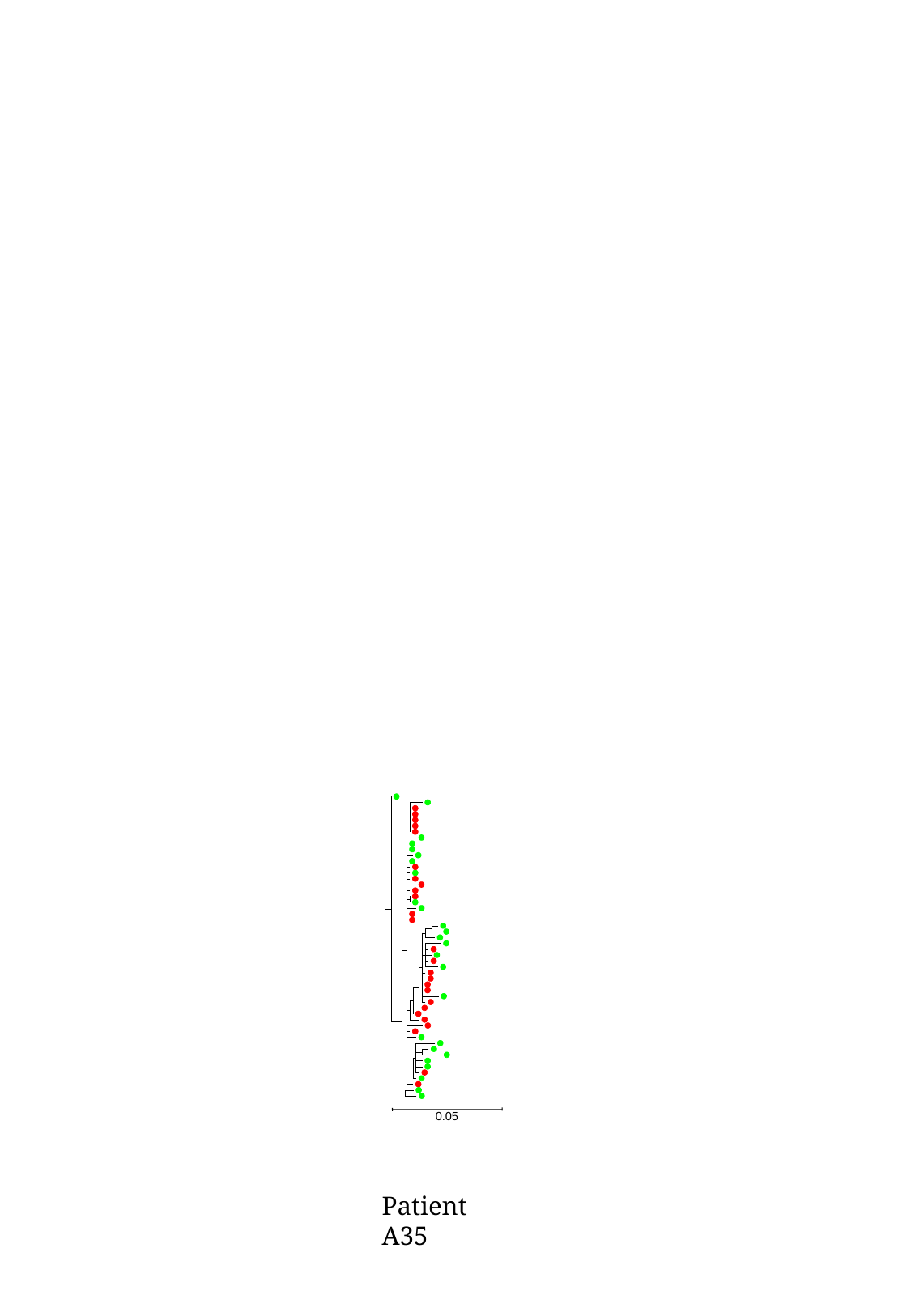

0.05
Patient A35

## Slide 10
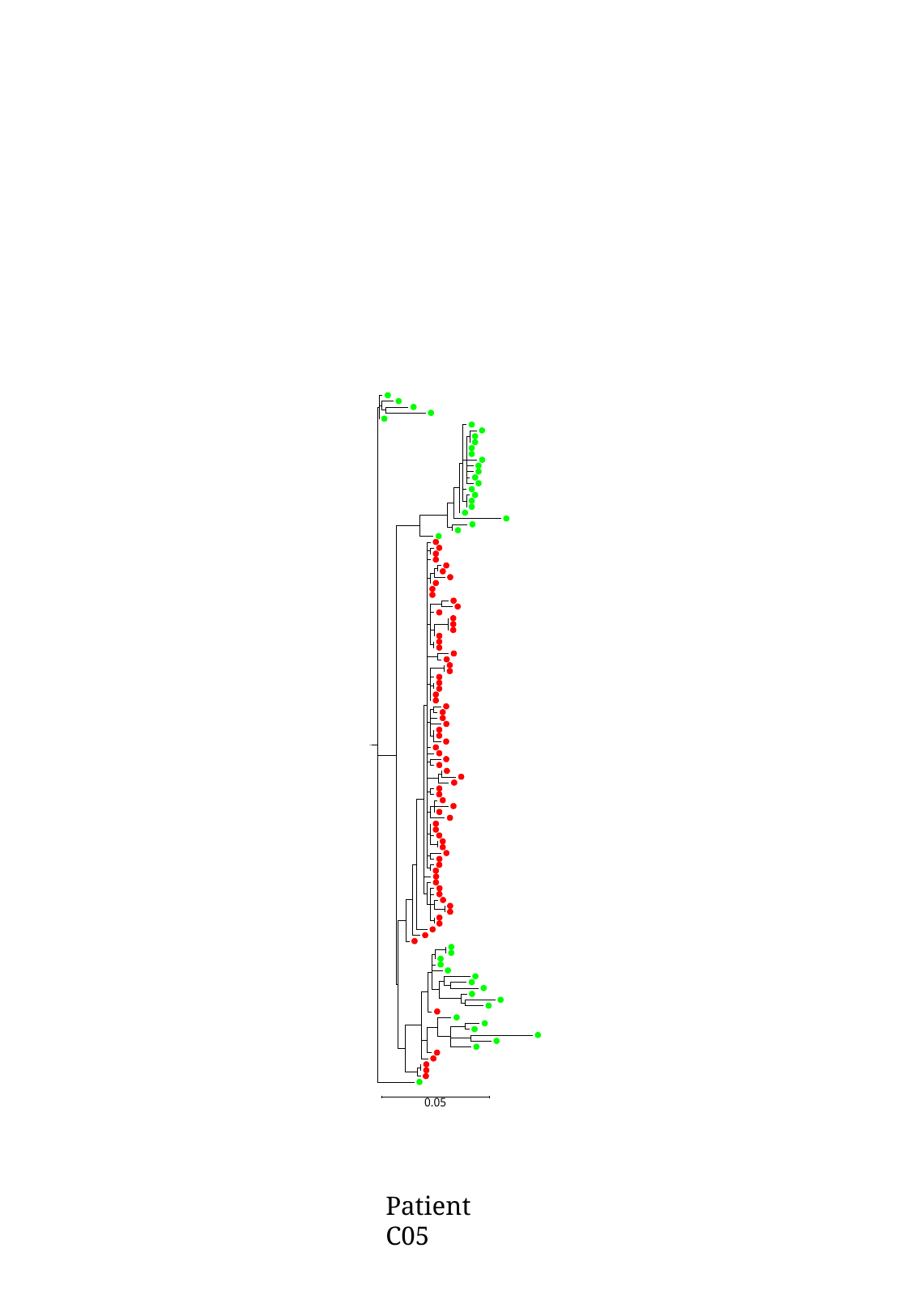

Patient C05

## Slide 11
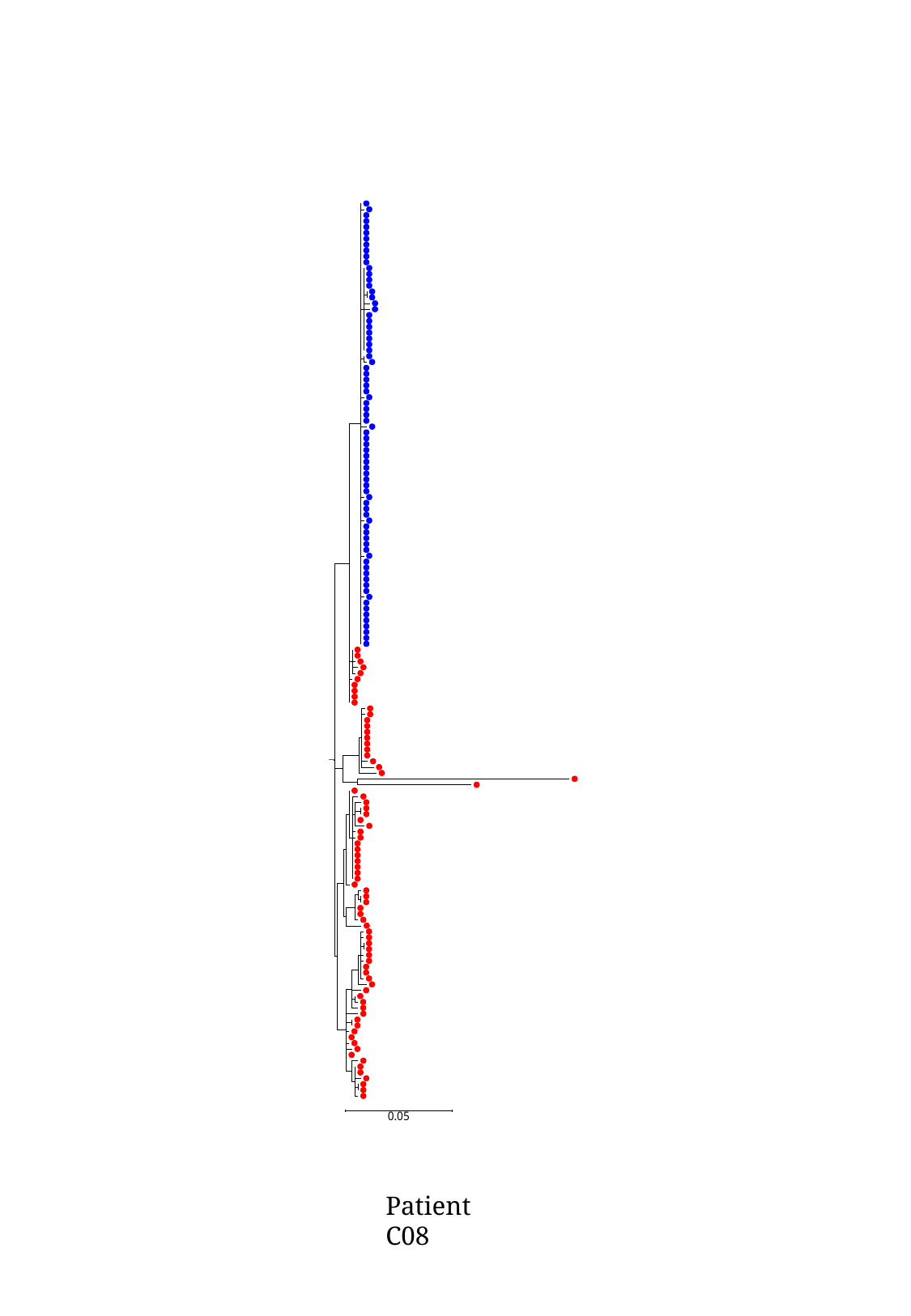

Patient C08

## Slide 12
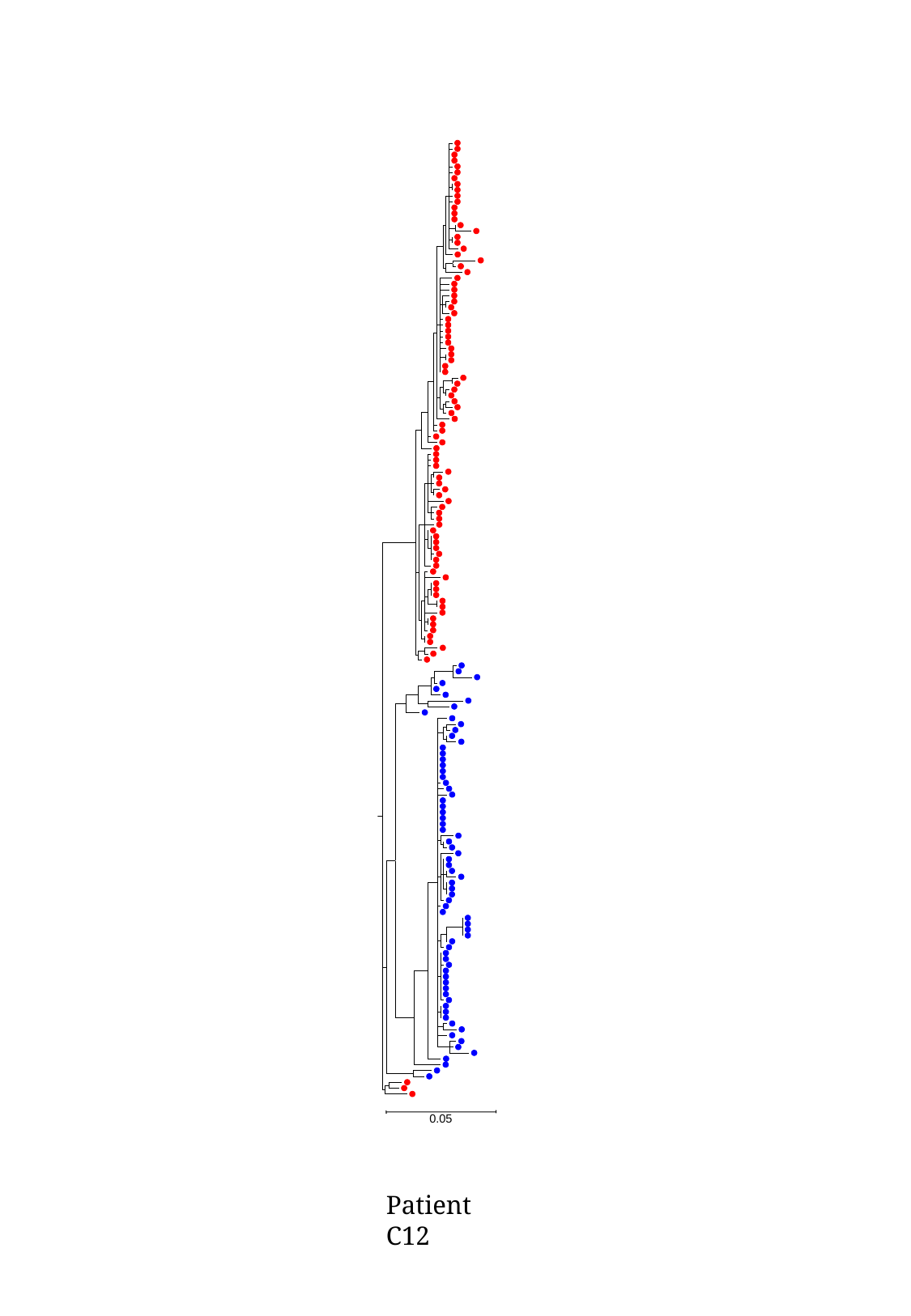

0.05
Patient C12

## Slide 13
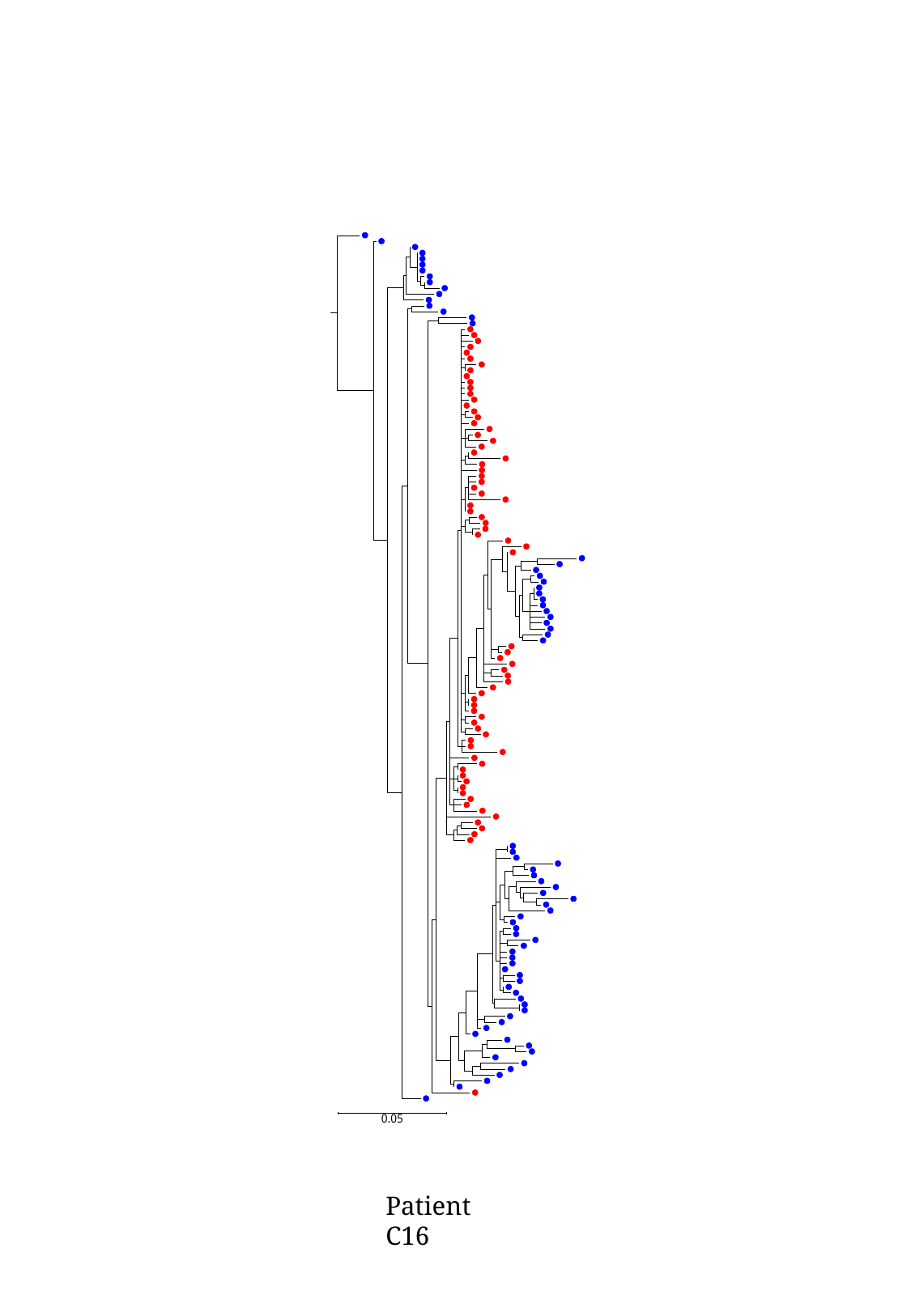

Patient C16

## Slide 14
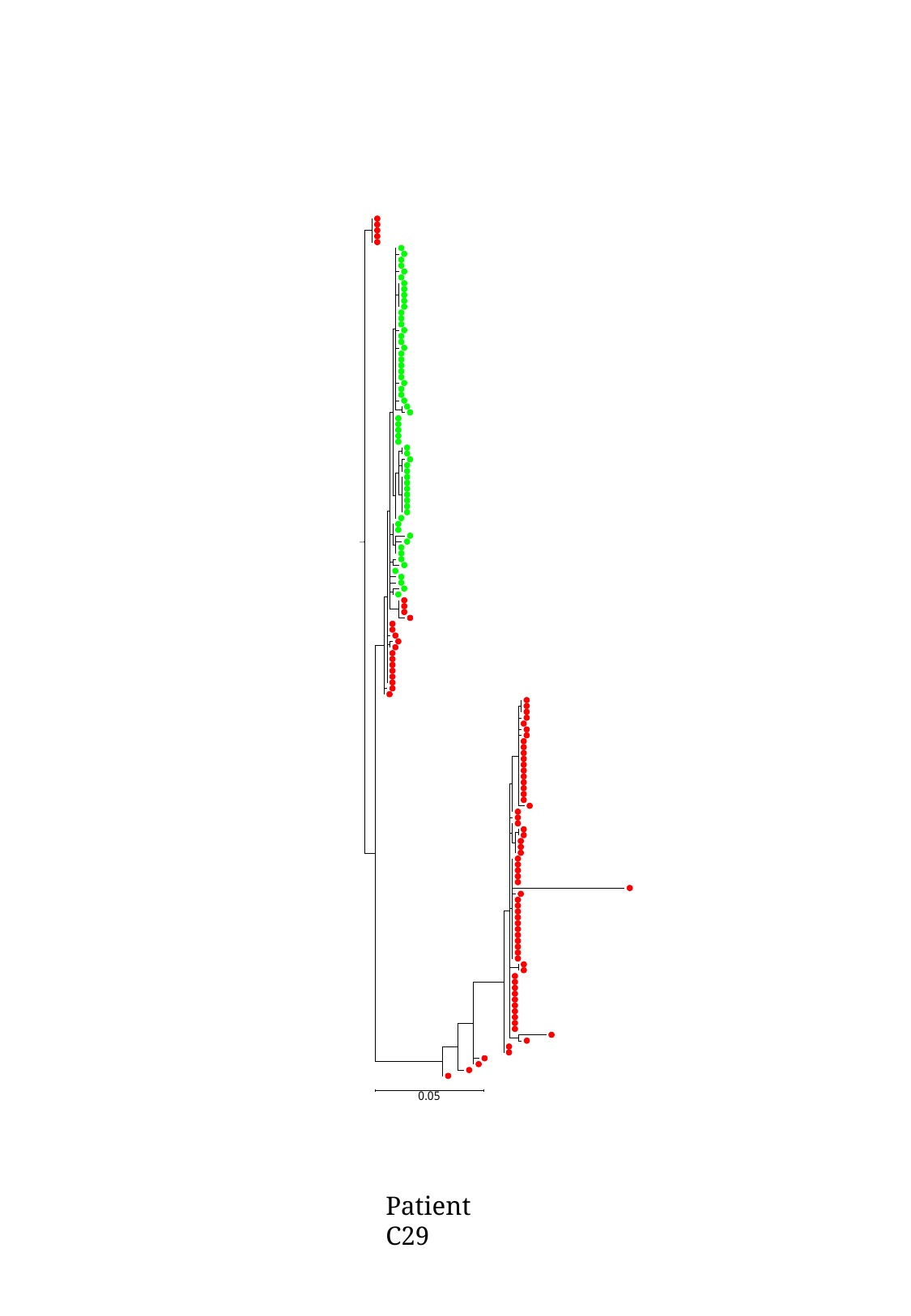

Patient C29

## Slide 15
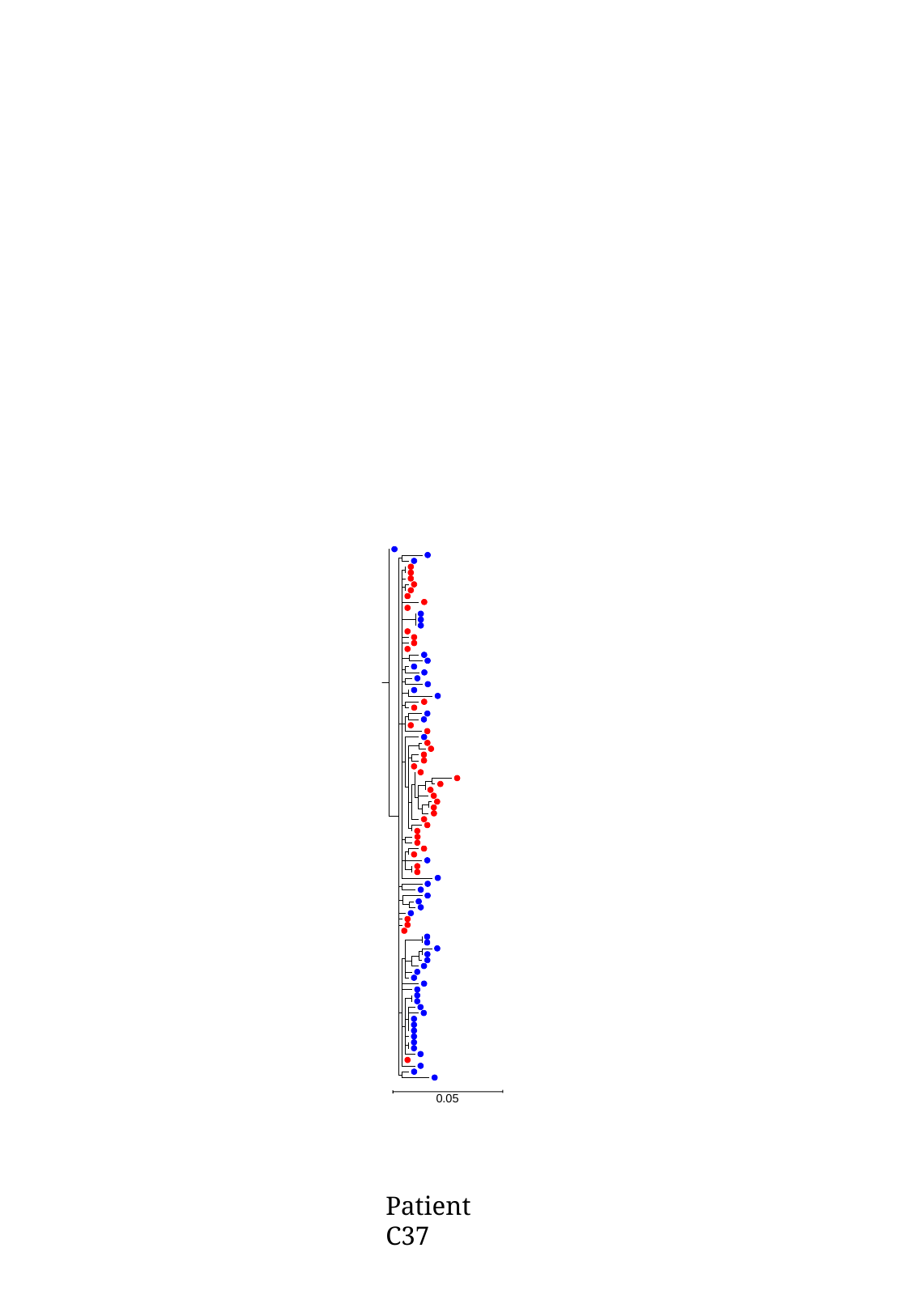

0.05
Patient C37

## Slide 16
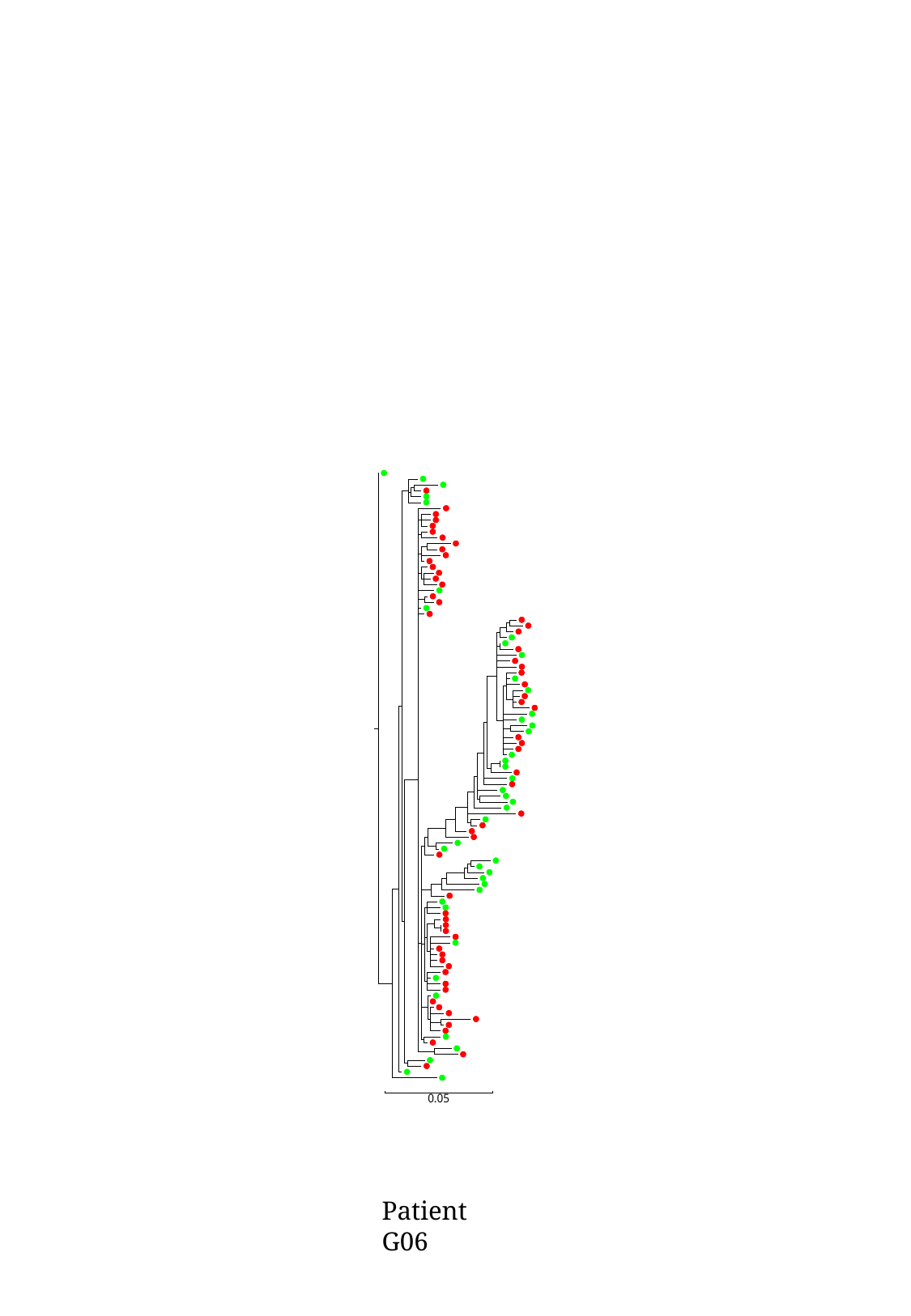

Patient G06

## Slide 17
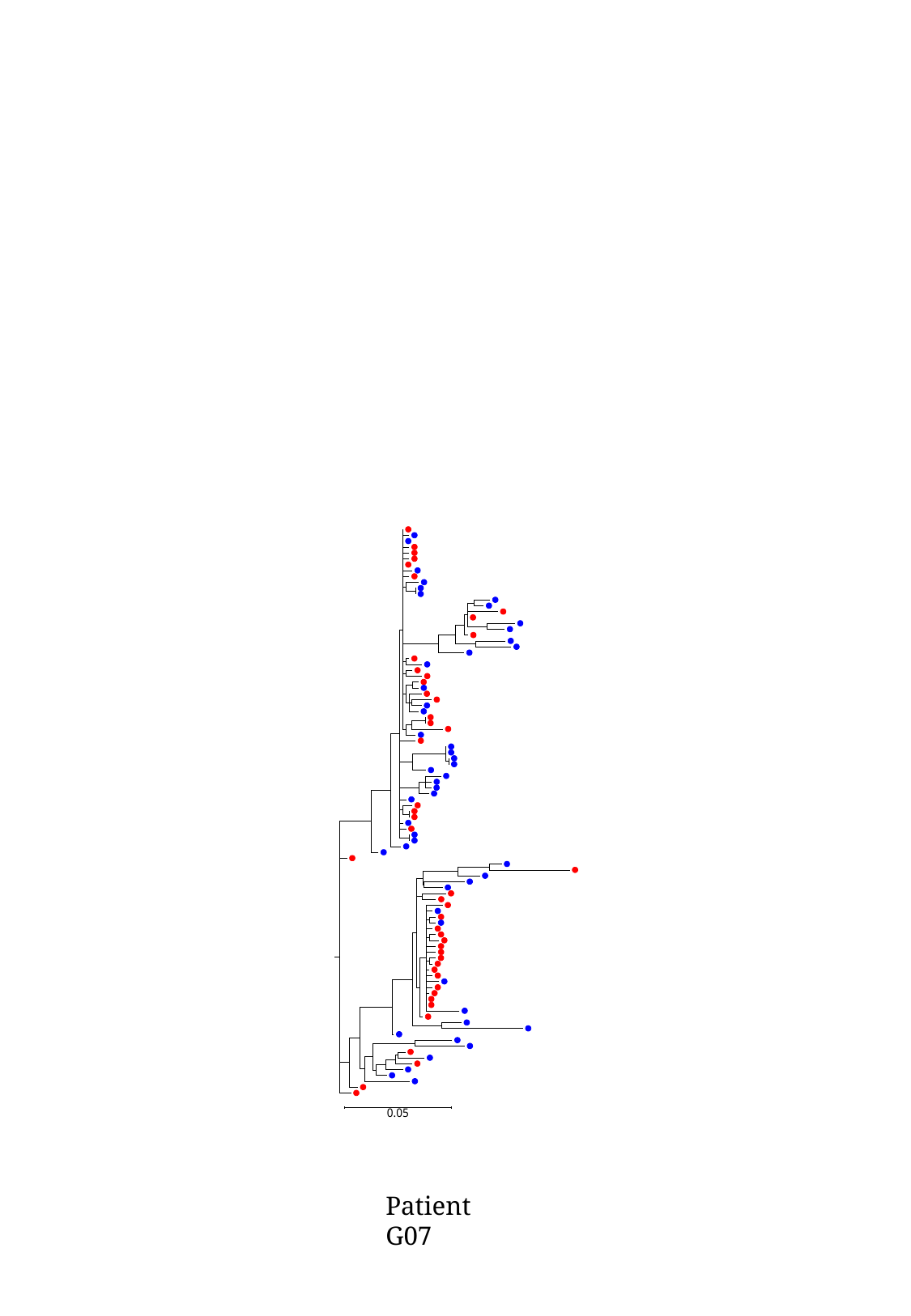

Patient G07

## Slide 18
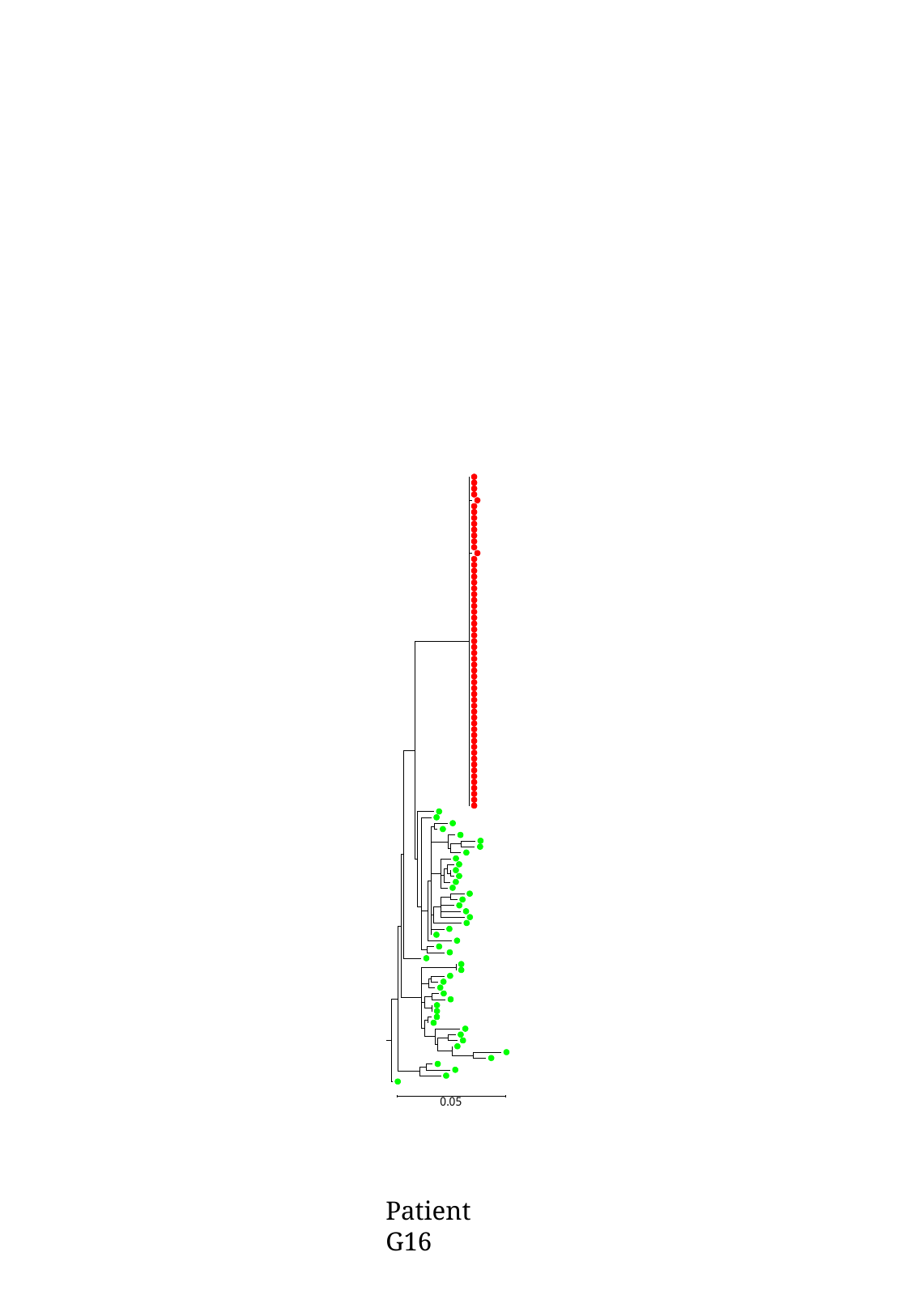

Patient G16

## Slide 19
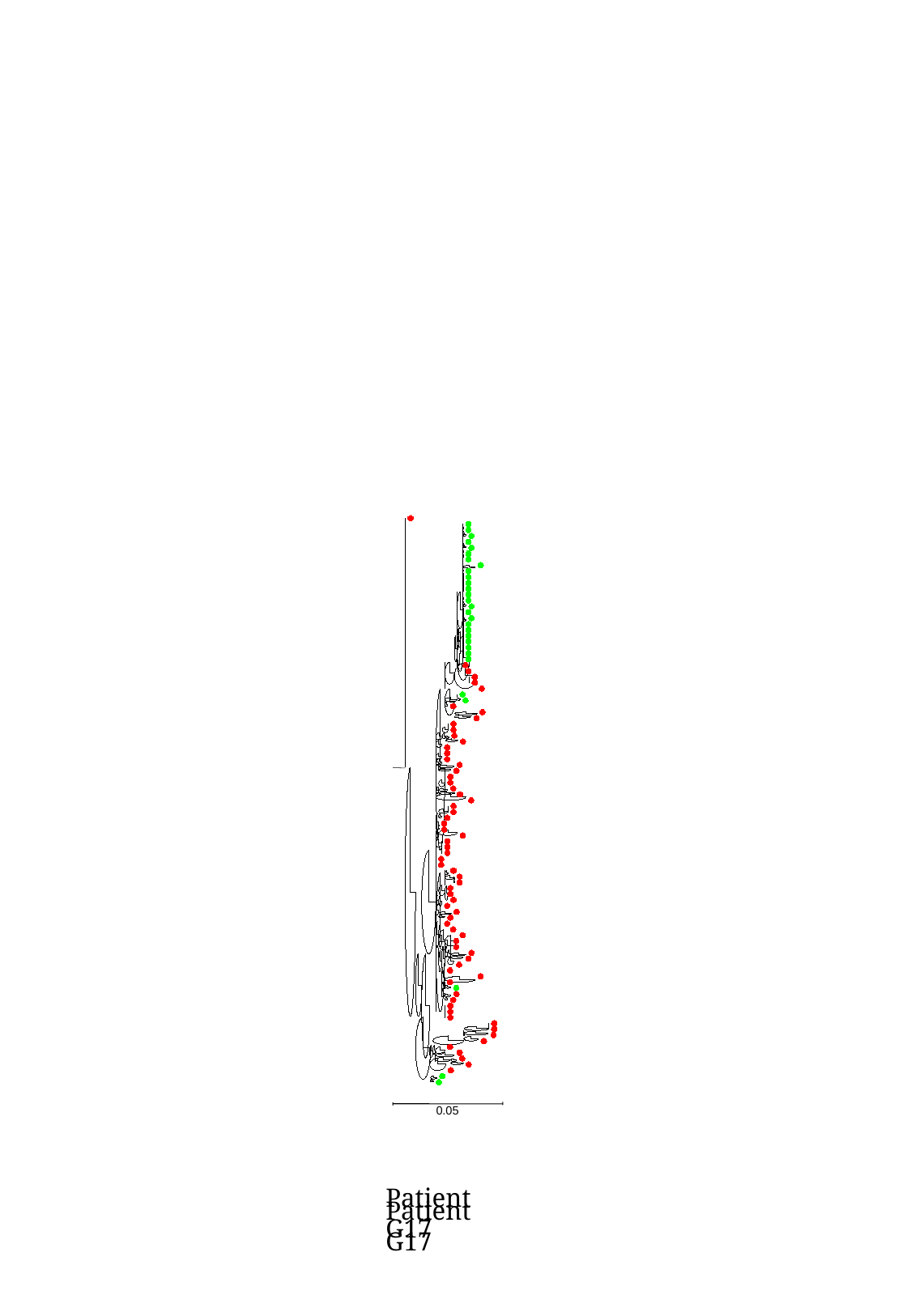

0.05
Patient G17
Patient G17

## Slide 20
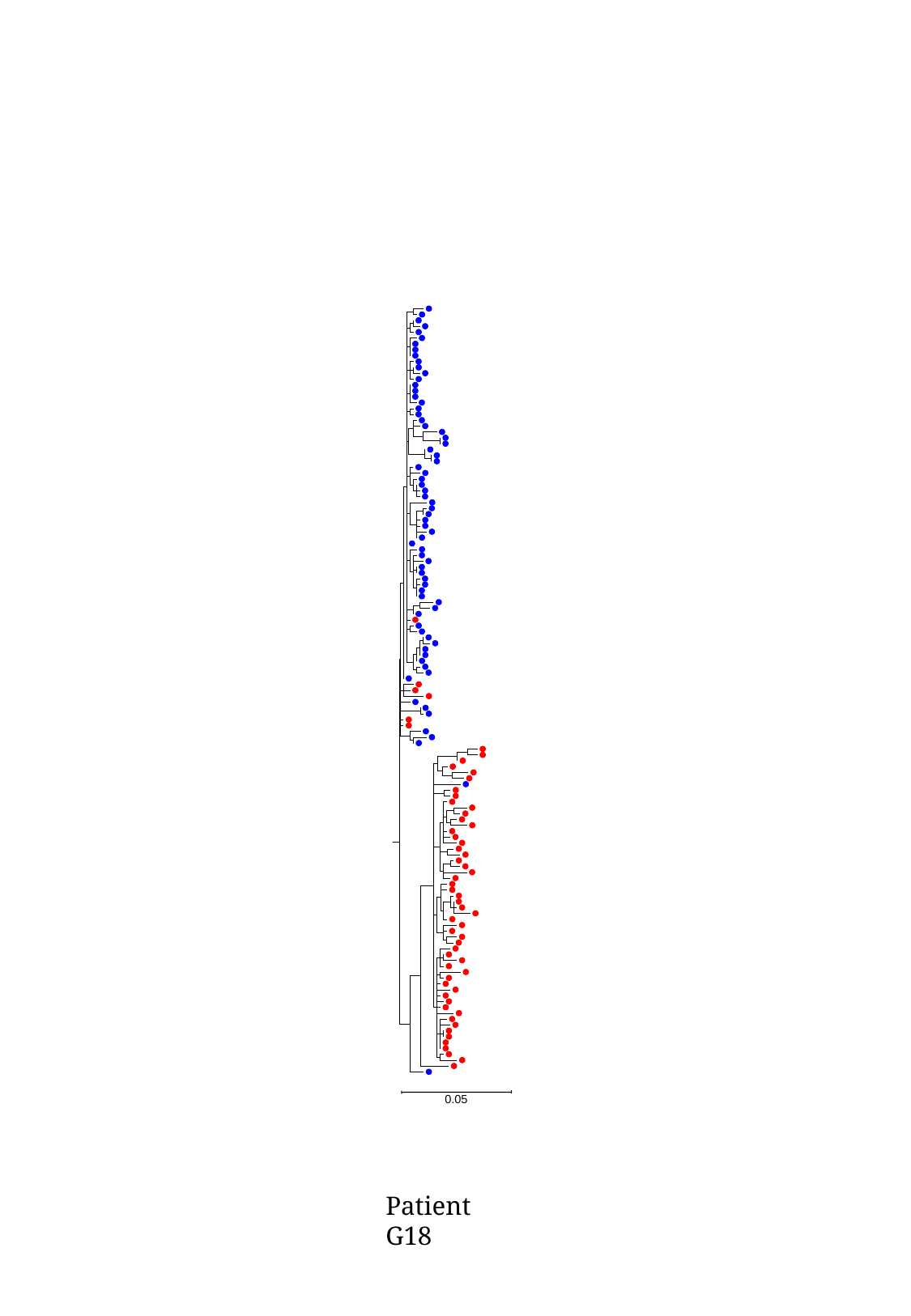

0.05
Patient G18

## Slide 21
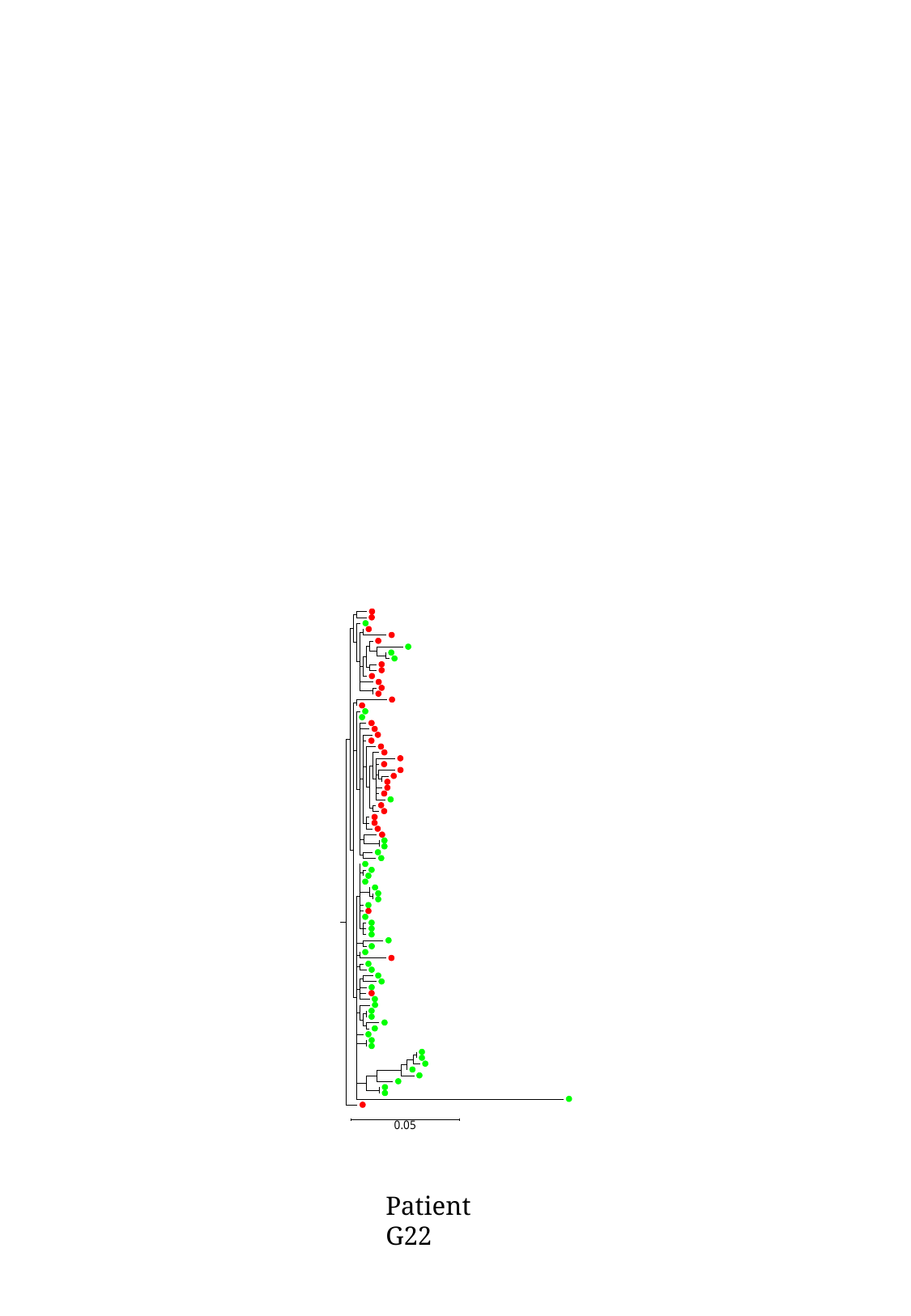

Patient G22

## Slide 22
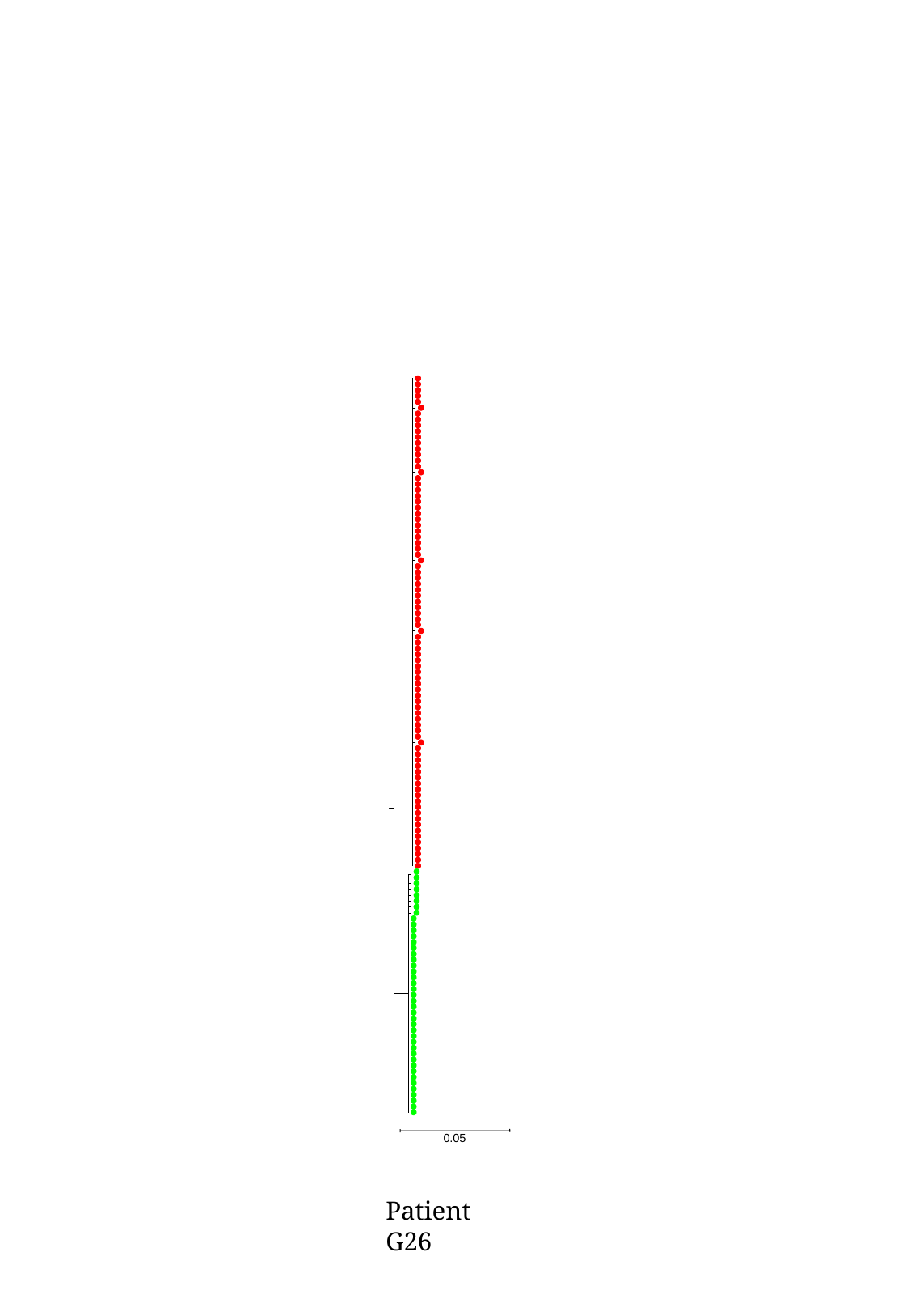

0.05
Patient G26
