# Supplementary material for: Genetic Variability of Hepatitis C Virus before and after Combined Therapy of Interferon plus Ribavirin
Source: PLoS One. 2008 Aug 26;3(8):e3058. doi: 10.1371/journal.pone.0003058 (PMC2518109; doi:10.1371/journal.pone.0003058)
Supplement: Table S1 — Genetic variability measures in the E1-E2 region of the HCV genome. (0.09 MB DOC) [file pone.0003058.s003.doc]

**Supplementary data**

**Table S1.** Genetic variability measures in the E1-E2 region of the HCV genome. T0 denotes samples before initiation of treatment and T1/T2 indicate those after 6/12 months of treatment. Abbreviations used: Seqs, number of clones analyzed; Acc. num.: GenBank accession numbers; S, number of polymorphic sites (for a fragment of 472 nucleotides); η, minimum number of mutations; Nhap, number of different haplotypes; π, nucleotide diversity; SD, standard deviation.

| Sample | Seqs | Acc. num. | S | η | Nhap | π | SD (π) |
| --- | --- | --- | --- | --- | --- | --- | --- |
| A09T0 | 100 | AM271252-271322 | 96 | 105 | 71 | 0.01237 | 0.00234 |
| A09T1 | 114 | AM707040-707154 | 131 | 147 | 86 | 0.02748 | 0.00298 |
| A20T0 | 112 | AM271587-271673 | 101 | 112 | 87 | 0.02403 | 0.00170 |
| A20T1 | 98 | AM707155-707252 | 102 | 123 | 82 | 0.03159 | 0.00142 |
| A21T0 | 100 | AM271674-271745 | 108 | 118 | 72 | 0.03865 | 0.00350 |
| A21T1 | 108 | AM707979-708086 | 157 | 171 | 84 | 0.06441 | 0.00117 |
| A34T0 | 100 | AM272228-272316 | 118 | 126 | 89 | 0.02922 | 0.00115 |
| A34T1 | 112 | AM707253-707364 | 110 | 123 | 101 | 0.02840 | 0.00091 |
| A35T0 | 109 | AM272317-272388 | 61 | 63 | 72 | 0.01173 | 0.00045 |
| A35T1 | 113 | AM707365-707477 | 117 | 122 | 98 | 0.01896 | 0.00073 |
| C05T0 | 100 | AM272497-272590 | 134 | 153 | 94 | 0.04689 | 0.00129 |
| C05T1 | 100 | AM708087-708186 | 109 | 119 | 65 | 0.02523 | 0.00316 |
| C08T0 | 100 | AM272699-272782 | 119 | 133 | 84 | 0.04604 | 0.00121 |
| C08T2 | 100 | AM708187-708186 | 27 | 27 | 31 | 0.00373 | 0.00030 |
| C12T0 | 100 | AM272988-273075 | 89 | 103 | 88 | 0.02486 | 0.00160 |
| C12T2 | 100 | AM708287-708386 | 87 | 90 | 76 | 0.03215 | 0.00057 |
| C16T0 | 101 | AM273267-273332 | 78 | 86 | 66 | 0.02513 | 0.00219 |
| C16T2 | 107 | AM708387-708493 | 110 | 122 | 90 | 0.04823 | 0.00095 |
| C17T0 | 106 | AM273333-273376 | 41 | 42 | 44 | 0.00799 | 0.00067 |
| C17T2 | 100 | AM713193-713292 | 14 | 14 | 20 | 0.00292 | 0.00050 |
| C22T0 | 100 | AM273635-273716 | 91 | 103 | 81 | 0.04310 | 0.00087 |
| C22T1 | 100 | AM707478-707577 | 67 | 67 | 69 | 0.01348 | 0.00097 |
| C22T2 | 101 | AM707578-707678 | 79 | 81 | 67 | 0.02047 | 0.00051 |
| C29T0 | 100 | AM273966-274001 | 68 | 71 | 36 | 0.03284 | 0.00406 |
| C29T1 | 100 | AM708494-708593 | 41 | 41 | 34 | 0.00431 | 0.00051 |
| C37T0 | 101 | AM274396-274431 | 27 | 27 | 35 | 0.00844 | 0.00070 |
| C37T2 | 100 | AM708594-708693 | 99 | 100 | 82 | 0.01283 | 0.00069 |
| G06T0 | 100 | AM274598-274694 | 112 | 123 | 97 | 0.02866 | 0.00118 |
| G06T1 | 100 | AM708694-708793 | 106 | 118 | 94 | 0.02301 | 0.00159 |
| G07T0 | 100 | AM274695-274778 | 103 | 109 | 84 | 0.03989 | 0.00167 |
| G07T2 | 100 | AM708794-708893 | 52 | 54 | 56 | 0.00774 | 0.00053 |
| G14T0 | 100 | AM274966-275025 | 102 | 106 | 60 | 0.02559 | 0.00282 |
| G14T1 | 100 | AM707779-707878 | 57 | 58 | 66 | 0.02260 | 0.00079 |
| G16T0 | 103 | AM275026-275028 | 2 | 2 | 3 | 0.00008 | 0.00006 |
| G16T1 | 100 | AM708894-708993 | 142 | 152 | 79 | 0.03138 | 0.00399 |
| G17T0 | 101 | AM275029-275117 | 97 | 101 | 89 | 0.05071 | 0.00148 |
| G17T1 | 100 | AM708994-709093 | 106 | 113 | 81 | 0.05329 | 0.00076 |
| G18T0 | 100 | AM275118-275204 | 104 | 110 | 87 | 0.03142 | 0.00131 |
| G18T2 | 100 | AM709094-709193 | 77 | 78 | 65 | 0.01010 | 0.00059 |
| G19T0 | 100 | AM275205-275266 | 80 | 82 | 62 | 0.02765 | 0.00275 |
| G19T2 | 100 | AM707879-707978 | 109 | 112 | 78 | 0.04254 | 0.00109 |
| G22T0 | 100 | AM271041-271115 | 88 | 93 | 75 | 0.01468 | 0.00229 |
| G22T1 | 102 | AM709194-709295 | 72 | 73 | 59 | 0.02872 | 0.00060 |
| G26T0 | 100 | AM271118-271126 | 8 | 8 | 9 | 0.00038 | 0.00012 |
| G26T1 | 100 | AM709296-709395 | 9 | 9 | 10 | 0.00051 | 0.00013 |
| G26T2 | 102 | AM709396-709497 | 93 | 101 | 58 | 0.01778 | 0.00314 |
