# Supplementary material for: Genetic Variability of Hepatitis C Virus before and after Combined Therapy of Interferon plus Ribavirin
Source: PLoS One. 2008 Aug 26;3(8):e3058. doi: 10.1371/journal.pone.0003058 (PMC2518109; doi:10.1371/journal.pone.0003058)
Supplement: Table S2 — Genetic variability measures in the NS5A region of the HCV genome. (0.09 MB DOC) [file pone.0003058.s004.doc]

**Supplementary data**

**Table S2.** Genetic variability measures in the NS5A region of the HCV genome. T0 denotes samples before initiation of treatment and T1/T2 indicate those after 6/12 months of treatment. Abbreviations used: Seqs, number of clones analyzed; Acc. num.: GenBank accession number; S, number of polymorphic sites (for a fragment of 743 nucleotides); η, minimum number of mutations; Nhap, number of different haplotypes; π, nucleotide diversity; SD, standard deviation.

| Sample | Seqs | Acc. num. | S | η | Nhap | π | SD (π) |
| --- | --- | --- | --- | --- | --- | --- | --- |
| A09T0 | 32 | AM282426-282453 | 52 | 53 | 28 | 0.01231 | 0.00168 |
| A09T1 | 34 | AM700592-700625 | 100 | 107 | 34 | 0.02122 | 0.00114 |
| A20T0 | 67 | AM279812-279843 | 34 | 34 | 32 | 0.00972 | 0.00105 |
| A20T1 | 33 | AM700626-700658 | 28 | 28 | 17 | 0.00281 | 0.00083 |
| A21T0 | 49 | AM279844-279858 | 35 | 36 | 15 | 0.00290 | 0.00106 |
| A21T1 | 64 | AM700964-701027 | 35 | 36 | 12 | 0.00245 | 0.00108 |
| A34T0 | 29 | AM280239-280267 | 59 | 61 | 29 | 0.01330 | 0.00076 |
| A34T1 | 39 | AM700659-700697 | 93 | 98 | 39 | 0.01846 | 0.00076 |
| A35T0 | 27 | AM280268-280287 | 28 | 29 | 20 | 0.00617 | 0.00060 |
| A35T1 | 25 | AM701028-701052 | 59 | 64 | 23 | 0.01210 | 0.00098 |
| C05T0 | 75 | AM280436-280497 | 72 | 77 | 62 | 0.01025 | 0.00086 |
| C05T1 | 43 | AM701053-701095 | 81 | 84 | 39 | 0.02404 | 0.00131 |
| C08T0 | 77 | AM280551-280602 | 144 | 155 | 52 | 0.01873 | 0.00216 |
| C08T2 | 76 | AM701096-701171 | 15 | 15 | 14 | 0.00102 | 0.00018 |
| C12T0 | 92 | AM280674-280748 | 114 | 119 | 75 | 0.01470 | 0.00067 |
| C12T2 | 71 | AM701172-701242 | 81 | 81 | 45 | 0.01380 | 0.00182 |
| C16T0 | 74 | AM280865-280933 | 124 | 130 | 69 | 0.01169 | 0.00079 |
| C16T2 | 74 | AM701243-701316 | 125 | 128 | 68 | 0.02529 | 0.00062 |
| C17T0 | 25 | AM280934-280939 | 6 | 6 | 6 | 0.00074 | 0.00029 |
| C17T2 | 85 | AM713293-713377 | 2 | 2 | 3 | 0.00006 | 0.00004 |
| C22T0 | 44 | AM281104-281145 | 98 | 108 | 42 | 0.01864 | 0.00110 |
| C22T1 | 25 | AM712211-712235 | 35 | 35 | 20 | 0.00671 | 0.00093 |
| C22T2 | 55 | AM700698-700752 | 55 | 67 | 50 | 0.00935 | 0.00085 |
| C29T0 | 87 | AM281392-281423 | 106 | 109 | 32 | 0.02972 | 0.00281 |
| C29T1 | 60 | AM701317-701376 | 31 | 31 | 27 | 0.00407 | 0.00032 |
| C37T0 | 42 | AM281685-281721 | 59 | 61 | 37 | 0.00842 | 0.00076 |
| C37T2 | 49 | AM701377-701425 | 113 | 114 | 42 | 0.01223 | 0.00071 |
| G06T0 | 61 | AM281829-281888 | 155 | 164 | 60 | 0.02628 | 0.00139 |
| G06T1 | 43 | AM701426-701468 | 116 | 121 | 42 | 0.02711 | 0.00087 |
| G07T0 | 48 | AM281889-281932 | 133 | 135 | 44 | 0.03625 | 0.00147 |
| G07T2 | 49 | AM701469-701517 | 145 | 151 | 45 | 0.03861 | 0.00257 |
| G14T0 | 84 | AM282015-282070 | 91 | 92 | 56 | 0.01080 | 0.00049 |
| G14T1 | 86 | AM700822-700907 | 117 | 122 | 79 | 0.01350 | 0.00071 |
| G16T0 | 57 | AM282071-282073 | 2 | 2 | 3 | 0.00009 | 0.00006 |
| G16T1 | 47 | AM701518-701564 | 121 | 125 | 45 | 0.02303 | 0.00087 |
| G17T0 | 68 | AM282074-282127 | 107 | 112 | 54 | 0.01546 | 0.00126 |
| G17T1 | 29 | AM701565-701593 | 30 | 30 | 11 | 0.00514 | 0.00169 |
| G18T0 | 60 | AM282128-282185 | 118 | 127 | 58 | 0.01521 | 0.00094 |
| G18T2 | 71 | AM701594-701664 | 93 | 94 | 62 | 0.01026 | 0.00077 |
| G19T0 | 52 | AM282186-282232 | 63 | 65 | 47 | 0.01068 | 0.00099 |
| G19T2 | 56 | AM700908-700963 | 84 | 86 | 49 | 0.00925 | 0.00076 |
| G22T0 | 36 | AM282283-282318 | 73 | 77 | 36 | 0.01249 | 0.00087 |
| G22T1 | 49 | AM701665-701713 | 107 | 111 | 41 | 0.01594 | 0.00243 |
| G26T0 | 84 | AM282322-282327 | 5 | 5 | 6 | 0.00016 | 0.00007 |
| G26T1 | 42 | AM701714-701755 | 7 | 7 | 8 | 0.00051 | 0.00015 |
