# Supplementary material for: Genetic Variability of Hepatitis C Virus before and after Combined Therapy of Interferon plus Ribavirin
Source: PLoS One. 2008 Aug 26;3(8):e3058. doi: 10.1371/journal.pone.0003058 (PMC2518109; doi:10.1371/journal.pone.0003058)
Supplement: Table S3 — Positions detected to change significantly in amino acid composition between samples at T0 and T1/T2 for each patient included in the study for the E1-E2 region. (0.26 MB DOC) [file pone.0003058.s005.doc]

**Table S3.** Positions detected to change significantly in amino acid composition between samples at T0 and T1/T2 for each patient included in the study for the E1-E2 region. Hypervariable regions are indicated by colour highlighting of the corresponding positions (yellow, HVR1; blue, HVR3; green, HVR2).

|  | Amino acid position | | | | | | | | | | | | | | | | | | | | | | | | | | | | | | | | | | | |
| --- | --- | --- | --- | --- | --- | --- | --- | --- | --- | --- | --- | --- | --- | --- | --- | --- | --- | --- | --- | --- | --- | --- | --- | --- | --- | --- | --- | --- | --- | --- | --- | --- | --- | --- | --- | --- |
|  | 2 | 3 | 8 | 13 | 17 | 18 | 22 | 32 | 38 | 44 | 56 | 57 | 58 | 59 | 60 | 61 | 62 | 63 | 64 | 65 | 66 | 67 | 68 | 69 | 70 | 71 | 72 | 73 | 74 | 75 | 76 | 77 | 78 | 80 | 81 | 83 |
| A09 |  |  |  |  |  |  |  |  |  |  |  | x |  | x |  |  |  |  | x |  |  |  |  | x | x | x | x | x |  | x |  | x |  |  |  | x |
| A20 |  |  |  |  |  |  |  |  |  |  |  |  |  |  |  |  |  |  |  |  |  |  |  |  |  |  |  |  |  |  |  |  |  |  |  |  |
| A21 |  |  |  |  |  |  |  |  |  |  |  | x |  | x |  |  |  |  | x | x | x | x | x | x | x | x |  | x | x | x | x | x | x |  |  | x |
| A34 |  |  |  |  |  |  |  |  |  |  |  |  |  |  |  |  |  |  |  |  |  |  |  |  |  |  |  |  |  |  |  |  |  |  |  |  |
| A35 |  |  |  |  |  |  |  |  |  |  |  |  |  |  |  |  |  | x |  | x |  | x |  |  |  |  |  |  |  |  |  |  |  |  |  |  |
| C05 |  | x |  |  |  |  |  |  |  |  |  | x |  | x | x |  |  |  |  |  |  | x | x |  | x |  | x |  | x |  |  | x | x | x |  | x |
| C08 |  |  |  |  | x |  |  |  |  |  |  | x | x | x | x |  |  |  | x | x |  | x | x | x | x | x | x |  | x |  |  | x | x | x |  |  |
| C12 |  |  |  |  |  | x |  |  |  |  |  | x |  | x |  | x |  |  | x | x | x | x | x | x | x | x | x | x |  | x | x | x |  |  |  |  |
| C16 | x |  |  |  |  |  |  |  |  |  |  | x |  | x | x | x |  |  | x | x | x | x | x |  | x | x | x | x | x | x |  |  | x |  |  |  |
| C17 |  |  |  |  |  |  |  |  |  |  |  |  |  | x | x |  |  |  |  | x |  |  |  |  | x |  | x | x | x | x | x |  | x |  |  |  |
| C22T0_T1 |  |  | x |  |  |  |  |  | x |  |  | x |  |  | x |  |  |  | x | x |  | x | x | x |  |  | x | x |  |  | x |  | x | x | x |  |
| C22T1_T2 |  |  |  | x |  |  |  |  | x |  |  | x |  |  |  |  |  |  |  | x |  | x | x |  | x | x |  |  |  |  |  |  |  |  |  |  |
| C29 |  |  |  |  | x |  |  |  |  | x |  | x |  | x | x | x | x |  | x | x | x | x | x |  | x | x |  | x |  |  |  | x | x |  |  |  |
| C37 |  |  |  |  |  |  |  |  |  |  |  |  |  |  |  |  |  |  |  |  |  |  |  |  |  |  |  |  |  |  |  |  |  |  |  |  |
| G06 |  |  |  |  |  |  |  |  |  |  |  |  |  |  |  |  |  |  |  |  |  |  |  |  |  |  |  |  |  |  |  |  |  |  |  |  |
| G07 |  |  |  |  |  |  |  |  | x |  | x |  |  | x |  |  |  |  | x | x |  |  |  |  | x |  | x | x | x |  |  | x |  |  | x |  |
| G14 |  |  | x |  |  |  |  | x |  |  |  | x |  | x |  |  |  |  | x | x |  | x |  |  |  |  | x | x |  |  |  | x | x | x |  | x |
| G16 |  |  |  |  |  |  |  |  |  |  |  | x |  | x | x |  |  | x | x |  |  | x | x | x | x | x | x | x | x | x | x |  | x |  |  | x |
| G17 |  |  |  |  |  |  |  |  |  |  |  |  |  |  |  |  |  |  |  |  |  |  |  |  |  |  |  |  |  |  |  |  |  |  |  |  |
| G18 |  |  |  |  |  |  | x |  |  |  |  |  |  | x |  |  |  |  |  |  |  | x |  |  |  |  |  | x |  |  |  |  | x |  | x |  |
| G19 |  |  |  |  |  |  |  |  |  |  |  |  |  |  |  |  |  |  |  | x | x | x | x | x |  | x | x | x | x |  |  |  |  |  | x |  |
| G22 |  |  |  |  |  |  |  |  |  |  |  |  |  |  |  |  |  |  |  |  |  |  |  |  | x |  |  |  | x |  |  |  | x | x | x |  |
| G26T0_T1 |  |  |  |  |  |  |  |  |  |  |  |  |  | x |  |  |  |  |  | x |  |  |  |  |  |  |  |  |  |  |  |  |  |  |  |  |
| G26T1_T2 |  |  |  |  |  |  |  |  |  |  |  |  |  | x |  |  |  |  | x | x |  |  | x |  |  |  |  |  |  |  |  |  |  |  |  |  |
| Total | 1 | 1 | 2 | 1 | 2 | 1 | 1 | 1 | 3 | 1 | 1 | 11 | 1 | 14 | 7 | 3 | 1 | 2 | 11 | 14 | 5 | 13 | 11 | 7 | 12 | 9 | 11 | 12 | 9 | 6 | 5 | 8 | 11 | 5 | 5 | 5 |

|  | Amino acid position | | | | | | | | | | | | | | | | | | | | | | | | | | | | | | | | | | | |
| --- | --- | --- | --- | --- | --- | --- | --- | --- | --- | --- | --- | --- | --- | --- | --- | --- | --- | --- | --- | --- | --- | --- | --- | --- | --- | --- | --- | --- | --- | --- | --- | --- | --- | --- | --- | --- |
|  | 84 | 87 | 88 | 89 | 90 | 93 | 99 | 103 | 105 | 106 | 107 | 111 | 112 | 115 | 116 | 117 | 118 | 119 | 120 | 122 | 123 | 126 | 127 | 130 | 133 | 134 | 137 | 139 | 144 | 146 | 148 | 151 | 152 | 153 | 154 | Total |
| A09 | x | x |  |  |  |  |  |  |  |  | x | x |  | x |  | x | x |  |  |  |  |  |  |  |  |  | x |  |  | x | x |  |  |  |  | 21 |
| A20 |  |  |  |  |  |  |  |  |  |  |  |  |  |  |  |  |  |  |  |  |  |  |  |  |  |  |  |  |  |  |  |  |  |  |  | 0 |
| A21 |  | x |  |  |  |  |  |  |  |  |  |  |  | x |  |  | x | x |  |  |  |  |  |  |  |  |  |  |  |  |  |  |  |  |  | 21 |
| A34 |  |  |  |  |  |  |  |  |  |  |  |  |  |  |  |  |  |  |  |  |  |  |  |  |  |  |  |  |  |  |  |  |  |  |  | 0 |
| A35 |  |  |  |  |  |  |  |  |  |  |  |  |  |  |  |  |  |  |  |  |  |  |  |  |  |  |  |  |  |  |  |  |  |  |  | 3 |
| C05 |  | x |  |  |  |  |  |  | x |  | x |  |  |  |  |  |  | x |  |  |  |  |  |  |  |  |  |  |  |  |  | x |  |  |  | 18 |
| C08 |  | x |  |  |  |  |  |  |  |  | x |  | x | x |  |  | x | x |  | x |  |  |  |  |  | x |  |  |  |  | x | x |  |  |  | 27 |
| C12 |  |  |  |  |  |  |  |  |  |  | x |  |  |  |  |  |  |  |  |  |  |  |  |  |  |  | x |  |  |  |  |  |  | x |  | 20 |
| C16 |  |  |  |  |  |  |  | x |  |  |  |  |  |  |  |  |  |  |  |  |  |  |  |  |  |  |  |  | x |  | x | x |  |  |  | 21 |
| C17 |  |  |  |  |  |  |  |  |  |  |  |  |  | x |  | x |  |  |  |  |  | x |  |  |  |  |  |  |  |  |  |  |  |  |  | 13 |
| C22T0_T1 |  |  |  |  |  |  |  |  |  |  | x | x |  | x |  | x |  | x |  | x |  |  |  |  | x |  |  |  |  |  |  |  |  |  | x | 23 |
| C22T1_T2 |  |  |  |  |  |  |  |  |  |  |  |  |  | x |  |  |  |  |  |  |  |  |  |  |  |  |  |  |  |  |  |  |  |  |  | 9 |
| C29 |  | x |  |  |  |  |  |  |  |  | x |  |  |  | x | x | x |  | x |  | x | x |  |  |  |  |  |  |  |  |  |  |  |  |  | 25 |
| C37 |  |  |  |  |  |  |  |  |  |  |  |  |  |  |  |  |  |  |  |  |  |  |  |  |  |  |  |  |  |  |  |  |  |  |  | 0 |
| G06 |  |  |  |  |  |  |  |  |  |  |  |  |  |  |  |  |  |  |  |  |  |  |  |  | x |  |  | x |  |  |  |  |  |  |  | 2 |
| G07 |  |  |  |  |  |  |  |  |  | x |  | x |  |  |  |  | x |  |  |  |  |  |  |  |  |  |  |  |  |  | x | x |  | x |  | 17 |
| G14 |  |  |  |  |  | x |  |  |  |  | x |  |  |  |  |  |  | x |  |  |  |  |  |  |  |  | x |  |  |  |  |  |  |  |  | 17 |
| G16 |  | x |  | x | x |  | x |  |  |  | x |  |  |  |  |  | x | x |  |  |  | x | x | x |  |  |  |  |  |  |  |  | x | x |  | 29 |
| G17 |  |  |  |  |  |  |  |  |  |  |  |  |  |  |  |  |  |  |  |  |  |  |  |  |  |  |  |  |  |  |  |  |  |  |  | 0 |
| G18 |  |  |  |  |  |  |  |  |  |  | x | x |  |  |  |  | x | x |  |  |  |  |  | x |  |  |  |  |  |  | x | x |  | x |  | 14 |
| G19 | x |  |  |  |  |  |  |  |  |  | x | x |  | x |  | x | x |  |  |  |  |  |  |  |  |  |  |  |  |  | x |  |  |  |  | 17 |
| G22 |  |  | x |  |  |  |  |  |  |  |  |  |  |  |  |  |  | x |  |  |  |  |  |  |  |  |  |  |  |  | x |  |  | x |  | 9 |
| G26T0_T1 |  |  |  |  |  |  |  |  |  |  |  |  |  |  |  |  |  |  |  |  |  |  |  |  |  |  |  |  |  |  | x | x |  |  |  | 4 |
| G26T1_T2 |  |  |  |  |  |  |  |  |  |  |  |  |  |  |  |  |  |  | x |  |  |  |  |  |  |  |  |  |  |  |  | x |  |  |  | 6 |
| Total | 2 | 6 | 1 | 1 | 1 | 1 | 1 | 1 | 1 | 1 | 10 | 5 | 1 | 7 | 1 | 5 | 8 | 8 | 2 | 2 | 1 | 3 | 1 | 2 | 2 | 1 | 3 | 1 | 1 | 1 | 8 | 7 | 1 | 5 | 1 |  |
