# Supplementary material for: Genetic Variability of Hepatitis C Virus before and after Combined Therapy of Interferon plus Ribavirin
Source: PLoS One. 2008 Aug 26;3(8):e3058. doi: 10.1371/journal.pone.0003058 (PMC2518109; doi:10.1371/journal.pone.0003058)
Supplement: Table S4 — Synonymous and non-synonymous substitutions levels in the six sub-regions of the E1-E2 region. (0.09 MB DOC) [file pone.0003058.s006.doc]

**Supplementary data**

**Table S4.** Synonymous (Ks) and non-synonymous (Ka) substitutions in the six sub-regions (E1, HVR1, E2_1, HVR3, E2_2, HVR2) of the E1-E2 region. For patients C22 and G26, two estimates were obtained (T0_T1 and T0_T2).

|  | E1 | |  | HVR1 | |  | E2_1 | |  | HVR3 | |  | E2_2 | |  | HVR2 | |
| --- | --- | --- | --- | --- | --- | --- | --- | --- | --- | --- | --- | --- | --- | --- | --- | --- | --- |
| Patient | *K_s_* | *K_a_* |  | *K_s_* | *K_a_* |  | *K_s_* | *K_a_* |  | *K_s_* | *K_a_* |  | *K_s_* | *K_a_* |  | *K_s_* | *K_a_* |
| A09 | 0.0406 | 0.0018 |  | 0.0932 | 0.0591 |  | 0.1092 | 0.0121 |  | 0.0485 | 0.0308 |  | 0.0612 | 0.0091 |  | 0.0487 | 0.0296 |
| A20 | 0.0351 | 0.0017 |  | 0.0837 | 0.1244 |  | 0.0156 | 0.0116 |  | 0.0540 | 0.0353 |  | 0.0190 | 0.0123 |  | 0.0771 | 0.0601 |
| A21 | 0.1356 | 0.0039 |  | 0.1505 | 0.2657 |  | 0.1918 | 0.0080 |  | 0.0399 | 0.0728 |  | 0.1391 | 0.0109 |  | 0.0994 | 0.0277 |
| A34 | 0.0581 | 0.0085 |  | 0.0433 | 0.0784 |  | 0.1214 | 0.0003 |  | 0.0232 | 0.0699 |  | 0.0200 | 0.0015 |  | 0.0288 | 0.0000 |
| A35 | 0.0302 | 0.0010 |  | 0.0248 | 0.0457 |  | 0.0215 | 0.0003 |  | 0.1001 | 0.0367 |  | 0.0807 | 0.0027 |  | 0.1162 | 0.0018 |
| C05 | 0.0377 | 0.0019 |  | 0.0655 | 0.0984 |  | 0.0246 | 0.0036 |  | 0.1205 | 0.0246 |  | 0.0758 | 0.0013 |  | 0.0187 | 0.0247 |
| C08 | 0.0146 | 0.0005 |  | 0.0029 | 0.0017 |  | 0.0039 | 0.0003 |  | 0.0429 | 0.0014 |  | 0.0012 | 0.0013 |  | 0.0000 | 0.0019 |
| C12 | 0.0505 | 0.0047 |  | 0.1058 | 0.1246 |  | 0.0956 | 0.0087 |  | 0.0246 | 0.0008 |  | 0.1055 | 0.0003 |  | 0.0197 | 0.0029 |
| C16 | 0.0363 | 0.0048 |  | 0.1468 | 0.2383 |  | 0.1216 | 0.0079 |  | 0.0684 | 0.0000 |  | 0.1428 | 0.0040 |  | 0.0181 | 0.0514 |
| C17 | 0.0018 | 0.0005 |  | 0.0000 | 0.0085 |  | 0.0098 | 0.0000 |  | 0.0053 | 0.0084 |  | 0.0193 | 0.0003 |  | 0.0000 | 0.0000 |
| C22T1 | 0.0366 | 0.0050 |  | 0.0655 | 0.0099 |  | 0.0493 | 0.0000 |  | 0.0361 | 0.0022 |  | 0.0358 | 0.0010 |  | 0.0810 | 0.0039 |
| C22T2 | 0.0335 | 0.0008 |  | 0.0686 | 0.0602 |  | 0.1123 | 0.0007 |  | 0.0208 | 0.0187 |  | 0.0338 | 0.0010 |  | 0.0709 | 0.0010 |
| C29 | 0.0145 | 0.0011 |  | 0.0133 | 0.0058 |  | 0.0108 | 0.0000 |  | 0.0066 | 0.0000 |  | 0.0107 | 0.0017 |  | 0.0080 | 0.0038 |
| C37 | 0.0281 | 0.0013 |  | 0.0491 | 0.0098 |  | 0.0570 | 0.0109 |  | 0.0190 | 0.0051 |  | 0.0245 | 0.0074 |  | 0.0308 | 0.0137 |
| G06 | 0.0560 | 0.0061 |  | 0.0653 | 0.0161 |  | 0.0554 | 0.0000 |  | 0.0529 | 0.0059 |  | 0.0532 | 0.0368 |  | 0.0071 | 0.0470 |
| G07 | 0.0247 | 0.0005 |  | 0.0223 | 0.0041 |  | 0.0221 | 0.0003 |  | 0.0211 | 0.0015 |  | 0.0121 | 0.0007 |  | 0.0595 | 0.0324 |
| G14 | 0.0671 | 0.0043 |  | 0.0967 | 0.0288 |  | 0.0101 | 0.0053 |  | 0.0298 | 0.0000 |  | 0.1038 | 0.0022 |  | 0.1639 | 0.0000 |
| G16 | 0.0401 | 0.0011 |  | 0.0670 | 0.1058 |  | 0.0512 | 0.0134 |  | 0.1031 | 0.0219 |  | 0.0727 | 0.0148 |  | 0.1106 | 0.0354 |
| G17 | 0.0655 | 0.0007 |  | 0.3088 | 0.1574 |  | 0.1041 | 0.0007 |  | 0.1053 | 0.0405 |  | 0.1217 | 0.0099 |  | 0.2315 | 0.0956 |
| G18 | 0.0287 | 0.0021 |  | 0.0220 | 0.0014 |  | 0.0445 | 0.0077 |  | 0.0490 | 0.0000 |  | 0.0536 | 0.0013 |  | 0.0151 | 0.0028 |
| G19 | 0.0666 | 0.0008 |  | 0.1769 | 0.0942 |  | 0.1254 | 0.0173 |  | 0.0992 | 0.1035 |  | 0.0735 | 0.0193 |  | 0.1568 | 0.0000 |
| G22 | 0.0522 | 0.0005 |  | 0.0639 | 0.0603 |  | 0.0867 | 0.0092 |  | 0.1097 | 0.0385 |  | 0.0714 | 0.0016 |  | 0.2826 | 0.0190 |
| G26T1 | 0.0000 | 0.0003 |  | 0.0000 | 0.0004 |  | 0.0000 | 0.0000 |  | 0.0000 | 0.0016 |  | 0.0012 | 0.0003 |  | 0.0000 | 0.0020 |
| G26T2 | 0.0148 | 0.0006 |  | 0.0237 | 0.0669 |  | 0.0339 | 0.0003 |  | 0.0242 | 0.0417 |  | 0.0148 | 0.0111 |  | 0.0909 | 0.0261 |
