# Supplementary material for: Genetic Variability of Hepatitis C Virus before and after Combined Therapy of Interferon plus Ribavirin
Source: PLoS One. 2008 Aug 26;3(8):e3058. doi: 10.1371/journal.pone.0003058 (PMC2518109; doi:10.1371/journal.pone.0003058)
Supplement: Table S5 — Relative change in the levels of synonymous and non-synonymous to synonymous substitutions in the six sub-regions of the E1-E2 region. (0.09 MB DOC) [file pone.0003058.s007.doc]

**Supplementary data**

**Table S5.** Relative change in the levels of synonymous (ΔKs=(Ks_T1_ – Ks_T0_)/Ks_T0_) and non-synonymous to synonymous (ΔKa/Ks) substitutions in the six sub-regions (E1, HVR1, E2_1, HVR3, E2_2, HVR2) of the E1-E2 region. For patients C22 and G26, two estimates were obtained (T0_T1 and T0_T2). Values in bold type represent absolute instead of relative changes in the corresponding levels because the corresponding value at T0 was equal to 0 or not computable.

|  | E1 | |  | HVR1 | |  | E2_1 | |  | HVR3 | |  | E2_2 | |  | HVR2 | |
| --- | --- | --- | --- | --- | --- | --- | --- | --- | --- | --- | --- | --- | --- | --- | --- | --- | --- |
| Patient | Δ*K_s_* | Δ*K_a_ /K_s_* |  | Δ*K_s_* | Δ*K_a_ /K_s_* |  | Δ*K_s_* | Δ*K_a_ /K_s_* |  | Δ*K_s_* | Δ*K_a_ /K_s_* |  | Δ*K_s_* | Δ*K_a_ /K_s_* |  | Δ*K_s_* | Δ*K_a_ /K_s_* |
| A09 | 0.7756 | -0.1096 |  | 0.8366 | 0.7799 |  | 0.9941 | -0.3674 |  | 2.1654 | -0.4231 |  | 1.1828 | -0.4090 |  | 3.5510 | 1.2484 |
| A20 | 0.1269 | 0.4124 |  | 0.3486 | -0.002 |  | -0.4367 | 1.2468 |  | 0.8027 | -0.2441 |  | 2.4436 | -0.6446 |  | 1.1224 | -0.4038 |
| A21 | 0.9922 | 0.0375 |  | 0.0757 | 0.6362 |  | 1.3217 | 2.389 |  | 4.8473 | -0.7337 |  | 0.5441 | 0.8296 |  | -0.3252 | 0.6422 |
| A34 | -0.1612 | 0.1101 |  | 0.187 | -0.0975 |  | -0.1029 | -0.4995 |  | 0.7724 | -0.3416 |  | -0.3839 | -0.3362 |  | 0.83 | -1 |
| A35 | 1.4 | -0.0671 |  | 0.9078 | 0.3838 |  | 2.5655 | **0.014** |  | 0.947 | -0.5441 |  | -0.0754 | 3.7232 |  | 0.1533 | **0.0151** |
| C05 | -0.4624 | -0.327 |  | -0.3036 | -0.3371 |  | -0.6841 | -0.4106 |  | 0.5031 | -0.542 |  | -0.239 | 0.0482 |  | -0.7795 | 5.9676 |
| C08 | -0.523 | -0.5818 |  | -0.9717 | -0.7099 |  | -0.8592 | -0.8316 |  | -0.5747 | -0.9563 |  | -0.9861 | 11.305 |  | -1 | -1 |
| C12 | 0.4341 | 2.3968 |  | 0.0533 | 0.2222 |  | 0.0316 | 2.5076 |  | -0.227 | -0.3516 |  | 1.4546 | -0.8984 |  | 1.3532 | -0.9176 |
| C16 | 0.4855 | 1.2637 |  | 0.6767 | 0.34 |  | 0.1564 | 0.0651 |  | -0.2552 | -1 |  | 2.7622 | -0.7066 |  | 0.1522 | 2.0898 |
| C17 | -0.9186 | 2.9201 |  | -1 | -1 |  | 0.9773 | -1 |  | **0.0053** | **1.5959** |  | 7.2364 | -0.9909 |  | -1 | **0** |
| C22T1 | -0.3216 | 0.9408 |  | -0.1149 | -0.919 |  | -0.5679 | -1 |  | -0.4366 | -0.9448 |  | -0.6635 | -0.7826 |  | -0.563 | -0.6597 |
| C22T2 | -0.3787 | -0.6554 |  | -0.0735 | -0.5296 |  | -0.0161 | -0.9467 |  | -0.6754 | -0.1773 |  | -0.6825 | -0.7718 |  | -0.6172 | -0.9008 |
| C29 | -0.5926 | -0.5301 |  | -0.5789 | -0.9146 |  | -0.8403 | -1 |  | -0.881 | -1 |  | -0.8672 | 0.1534 |  | -0.8852 | **0.4713** |
| C37 | 0.5456 | 0.303 |  | 0.196 | -0.4008 |  | 6.7714 | -0.8866 |  | **0.019** | **0.2687** |  | 0.9977 | 0.4798 |  | -0.4226 | **0.4455** |
| G06 | -0.0849 | 0.4717 |  | 0.0585 | -0.4195 |  | -0.2498 | -0.9468 |  | 0.4535 | -0.6495 |  | -0.4304 | 0.2451 |  | -0.3063 | 0.0125 |
| G07 | -0.6842 | -0.818 |  | -0.8809 | -0.6142 |  | -0.9026 | 4.1241 |  | -0.8281 | -0.7413 |  | -0.612 | -0.3553 |  | -0.5483 | 1.8172 |
| G14 | 0.9569 | -0.381 |  | 0.114 | -0.6322 |  | -0.7655 | 4.802 |  | 1.586 | -1 |  | 0.4072 | -0.892 |  | -0.2602 | -1 |
| G16 | **0.0401** | **0.0283** |  | **0.067** | **1.5809** |  | **0.0512** | **0.2621** |  | **0.1031** | **0.2123** |  | **0.0727** | **0.2038** |  | **0.1106** | **0.3202** |
| G17 | 0.192 | -0.5766 |  | -0.0377 | 0.0222 |  | -0.1741 | N.A. |  | 0.3259 | -0.1474 |  | 0.5755 | -0.2617 |  | 0.0675 | 0.0024 |
| G18 | -0.4349 | -0.1865 |  | -0.7844 | -0.8811 |  | 0.4792 | -0.6812 |  | -0.651 | -1 |  | -0.1063 | -0.844 |  | -0.8924 | -0.5798 |
| G19 | 0.7324 | -0.7228 |  | 1.6749 | -0.4386 |  | -0.1106 | 1.0986 |  | 1.0638 | -0.1112 |  | 1.0671 | -0.3366 |  | 1.3812 | -1 |
| G22 | 0.3051 | -0.4311 |  | 1.4307 | -0.0505 |  | 2.0562 | -0.3034 |  | 0.3469 | 36.433 |  | 2.0014 | 0.5832 |  | 0.7722 | -0.7588 |
| G26T1 | **0** | **0** |  | **0** | **0** |  | **0** | **0** |  | **0** | **0** |  | -0.0002 | 0.0002 |  | -1 | **0** |
| G26T2 | **0.0148** | **0.0434** |  | **0.0237** | **2.8182** |  | **0.0339** | **0.0098** |  | **0.0242** | **1.7184** |  | 11.319 | 1.6962 |  | 23.111 | **0.287** |
