# Supplementary material for: Genetic Variability of Hepatitis C Virus before and after Combined Therapy of Interferon plus Ribavirin
Source: PLoS One. 2008 Aug 26;3(8):e3058. doi: 10.1371/journal.pone.0003058 (PMC2518109; doi:10.1371/journal.pone.0003058)
Supplement: Table S6 — Positions detected to change significantly in amino acid composition between samples at T0 and T1/T2 for each patient included in the study for the NS5A region. (0.18 MB DOC) [file pone.0003058.s008.doc]

**Table S6.** Positions detected to change significantly in amino acid composition between samples at T0 and T1/T2 for each patient included in the study for the NS5A region. The most relevant regions are indicated by colour highlighting of the corresponding positions (yellow, ISDR; blue, rest of PKR-BD; green, V3 domain).

|  | Amino acid position | | | | | | | | | | | | | | | | | | | | | | | | | | | | | | | | |
| --- | --- | --- | --- | --- | --- | --- | --- | --- | --- | --- | --- | --- | --- | --- | --- | --- | --- | --- | --- | --- | --- | --- | --- | --- | --- | --- | --- | --- | --- | --- | --- | --- | --- |
|  | 16 | 31 | 34 | 47 | 49 | 71 | 74 | 76 | 78 | 79 | 80 | 81 | 85 | 86 | 89 | 99 | 114 | 119 | 122 | 123 | 125 | 145 | 149 | 159 | 160 | 162 | 165 | 169 | 172 | 181 | 182 | 191 | 194 |
| A09 |  |  |  |  |  |  |  |  |  |  |  |  |  |  |  |  |  |  |  |  |  |  |  |  | x | x |  |  |  |  |  |  |  |
| A20 |  |  |  |  | x |  |  |  |  |  |  |  |  |  |  |  |  |  |  |  |  |  |  |  |  |  |  |  |  |  |  |  |  |
| A21 |  |  |  |  | x |  |  |  |  |  |  |  |  |  |  |  |  |  |  |  |  |  |  |  |  |  |  |  |  |  |  |  |  |
| A34 |  |  |  |  |  |  |  |  |  |  |  |  |  |  |  |  |  |  |  |  |  |  |  |  |  |  |  |  |  |  |  |  |  |
| A35 |  |  |  |  |  |  |  |  |  |  |  |  |  |  |  |  |  |  |  |  |  |  |  |  |  |  |  |  |  |  |  |  |  |
| C05 |  |  |  |  |  | x | x |  | x |  | x |  |  |  |  |  |  |  |  |  |  | x |  |  |  |  |  |  |  |  |  |  |  |
| C08 |  |  | x |  |  |  |  |  |  |  |  |  | x |  |  |  | x |  |  |  |  |  | x |  |  |  |  |  |  |  |  | x |  |
| C12 |  |  |  |  |  |  |  |  |  |  | x |  |  |  |  |  | x |  |  |  |  |  | x |  |  |  |  | x | x |  |  |  |  |
| C16 |  |  |  | x |  |  |  |  |  |  |  |  |  |  |  |  | x |  |  |  |  |  |  | x |  | x |  |  |  |  |  |  |  |
| C17 |  |  |  |  |  |  |  |  |  |  |  |  |  |  |  |  |  |  |  |  |  |  |  |  |  |  |  |  |  |  |  |  |  |
| C22T0_T1 |  |  |  |  |  |  |  |  |  |  |  |  |  |  |  |  |  |  |  |  |  | x |  |  |  |  |  |  |  |  |  |  |  |
| C22T1_T2 |  |  |  |  |  |  |  |  |  |  |  |  |  |  |  |  |  |  |  |  |  | x |  |  |  |  |  |  |  |  |  |  |  |
| C29 |  | x |  |  |  | x | x | x |  | x |  | x |  | x | x | x |  | x | x | x |  |  |  |  |  | x |  |  |  |  | x |  | x |
| C37 |  |  |  |  |  |  |  |  |  |  |  |  |  |  |  |  |  |  |  |  |  |  |  |  |  | x | x |  |  |  |  |  |  |
| G06 |  |  |  |  |  |  |  |  |  |  |  |  |  |  |  |  |  |  |  |  |  |  |  |  |  |  |  |  |  |  |  |  |  |
| G07 |  |  |  |  |  |  |  |  |  |  |  |  |  |  |  |  |  |  |  |  |  |  |  |  |  |  |  |  |  |  |  |  |  |
| G14 |  |  |  |  |  |  |  |  |  |  |  |  |  |  |  |  | x |  |  |  |  |  |  |  |  |  |  |  |  |  |  |  |  |
| G16 |  |  |  |  | x |  |  |  |  |  |  |  |  |  |  |  |  |  |  |  | x |  |  |  |  |  |  |  |  |  |  |  |  |
| G17 | x |  |  |  |  |  |  |  |  |  |  |  |  |  |  |  |  |  |  |  |  |  |  |  |  |  |  |  |  |  |  |  |  |
| G18 |  |  |  |  |  |  |  |  |  |  | x |  |  |  |  |  |  |  |  |  |  |  | x |  |  |  |  |  |  | x |  |  |  |
| G19 |  |  |  |  |  |  |  |  |  |  |  |  |  |  |  |  | x |  |  |  |  |  |  |  |  |  |  |  |  |  |  |  |  |
| G22 |  |  |  |  |  |  |  |  |  |  |  |  |  |  |  |  |  |  |  |  |  |  |  |  |  |  |  |  |  |  |  |  |  |
| G26 |  |  |  |  |  |  |  |  |  |  | x |  |  |  |  |  |  |  |  |  |  |  |  |  |  |  |  |  |  |  |  |  |  |
| Total | 1 | 1 | 1 | 1 | 3 | 2 | 2 | 1 | 1 | 1 | 4 | 1 | 1 | 1 | 1 | 1 | 5 | 1 | 1 | 1 | 1 | 3 | 3 | 1 | 1 | 4 | 1 | 1 | 1 | 1 | 1 | 1 | 1 |

|  | Amino acid position | | | | | | | | | | | | | | | |
| --- | --- | --- | --- | --- | --- | --- | --- | --- | --- | --- | --- | --- | --- | --- | --- | --- |
|  | 218 | 220 | 222 | 223 | 226 | 232 | 234 | 235 | 236 | 239 | 240 | 241 | 244 | 245 | 247 | Total |
| A09 |  |  |  |  |  |  |  |  |  |  |  |  |  |  | x | 3 |
| A20 |  |  |  |  |  |  |  |  |  |  |  |  |  |  |  | 1 |
| A21 |  |  |  |  |  |  |  |  |  |  |  |  |  |  |  | 1 |
| A34 |  |  |  |  |  |  |  |  |  |  |  |  |  |  |  | 0 |
| A35 |  |  |  |  |  |  |  |  |  |  |  |  |  |  |  | 0 |
| C05 | x |  |  |  |  |  |  |  | x |  | x | x |  |  |  | 9 |
| C08 | x |  | x |  |  |  | x | x |  |  | x |  |  |  |  | 10 |
| C12 |  |  | x |  |  |  |  |  | x |  |  |  |  |  |  | 7 |
| C16 |  |  |  |  |  |  |  |  |  |  | x |  |  |  |  | 5 |
| C17 |  |  |  |  |  |  |  |  |  |  |  |  |  |  |  | 0 |
| C22T0_T1 |  |  |  |  |  |  |  |  |  |  |  |  |  |  |  | 1 |
| C22T1_T2 |  |  |  |  |  |  |  |  |  |  |  |  |  |  |  | 1 |
| C29 | x | x |  | x |  | x |  |  |  |  | x |  | x |  | x | 22 |
| C37 |  |  |  |  |  |  |  |  |  |  |  |  |  |  |  | 2 |
| G06 |  |  |  |  |  |  |  |  |  |  |  |  |  |  |  | 0 |
| G07 |  |  |  |  |  |  |  |  |  |  |  |  |  |  |  | 0 |
| G14 |  |  |  |  |  |  |  |  |  |  |  |  |  |  |  | 1 |
| G16 | x |  |  |  |  |  | x | x | x |  |  |  | x | x |  | 8 |
| G17 |  |  |  |  |  |  |  |  |  |  |  |  |  |  |  | 1 |
| G18 |  |  |  |  |  |  |  |  |  |  |  |  |  |  |  | 3 |
| G19 |  |  |  |  | x |  |  |  |  | x |  |  |  |  |  | 3 |
| G22 |  |  |  |  |  |  |  |  |  |  |  |  |  |  |  | 0 |
| G26 |  |  |  |  |  |  |  |  |  | x |  |  |  |  |  | 2 |
| Total | 4 | 1 | 2 | 1 | 1 | 1 | 2 | 2 | 3 | 2 | 4 | 1 | 2 | 1 | 2 |  |
