# Supplementary material for: Genetic Variability of Hepatitis C Virus before and after Combined Therapy of Interferon plus Ribavirin
Source: PLoS One. 2008 Aug 26;3(8):e3058. doi: 10.1371/journal.pone.0003058 (PMC2518109; doi:10.1371/journal.pone.0003058)
Supplement: Table S7 — Synonymous and non-synonymous substitutions levels in the five sub-regions of the NS5A region. (0.08 MB DOC) [file pone.0003058.s009.doc]

**Supplementary data**

**Table S7.** Synonymous (Ks) and non-synonymous (Ka) substitutions in the five sub-regions (NS5A_1, ISDR, Rest of PKR-BD, NS5A_2, V3) considered in the NS5A region. Two comparisons were performed for patient C22 (T0_T1 and T0_T2).

|  | NS5A_1 | |  | ISDR | |  | Rest of PKR-BD | |  | NS5A_2 | |  | V3 | |
| --- | --- | --- | --- | --- | --- | --- | --- | --- | --- | --- | --- | --- | --- | --- |
| Patient | *K_s_* | *K_a_* |  | *K_s_* | *K_a_* |  | *K_s_* | *K_a_* |  | *K_s_* | *K_a_* |  | *K_s_* | *K_a_* |
| A09 | 0.0695 | 0.0012 |  | 0.0643 | 0.0000 |  | 0.1181 | 0.0000 |  | 0.0695 | 0.0082 |  | 0.0591 | 0.0111 |
| A20 | 0.0064 | 0.0000 |  | 0.0023 | 0.0007 |  | 0.0033 | 0.0000 |  | 0.0128 | 0.0000 |  | 0.0128 | 0.0035 |
| A21 | 0.0068 | 0.0002 |  | 0.0072 | 0.0000 |  | 0.0000 | 0.0010 |  | 0.0073 | 0.0011 |  | 0.0039 | 0.0066 |
| A34 | 0.0542 | 0.0012 |  | 0.0502 | 0.0145 |  | 0.0243 | 0.0000 |  | 0.0569 | 0.0020 |  | 0.0624 | 0.0405 |
| A35 | 0.0136 | 0.0005 |  | 0.0469 | 0.0000 |  | 0.0434 | 0.0014 |  | 0.0411 | 0.0090 |  | 0.0601 | 0.0103 |
| C05 | 0.0566 | 0.0018 |  | 0.0951 | 0.0249 |  | 0.1430 | 0.0000 |  | 0.0443 | 0.0062 |  | 0.0807 | 0.0447 |
| C08 | 0.0019 | 0.0002 |  | 0.0010 | 0.0003 |  | 0.0050 | 0.0000 |  | 0.0055 | 0.0005 |  | 0.0000 | 0.0010 |
| C12 | 0.0369 | 0.0007 |  | 0.0416 | 0.0000 |  | 0.0556 | 0.0000 |  | 0.0564 | 0.0050 |  | 0.0640 | 0.0079 |
| C16 | 0.0733 | 0.0042 |  | 0.1252 | 0.0000 |  | 0.1140 | 0.0089 |  | 0.0698 | 0.0051 |  | 0.1344 | 0.0097 |
| C17 | 0.0000 | 0.0000 |  | 0.0000 | 0.0000 |  | 0.0000 | 0.0000 |  | 0.0000 | 0.0001 |  | 0.0000 | 0.0000 |
| C22T1 | 0.0182 | 0.0010 |  | 0.0030 | 0.0009 |  | 0.0123 | 0.0000 |  | 0.0228 | 0.0038 |  | 0.0344 | 0.0115 |
| C22T2 | 0.0298 | 0.0026 |  | 0.0138 | 0.0000 |  | 0.0242 | 0.0000 |  | 0.0387 | 0.0031 |  | 0.0394 | 0.0054 |
| C29 | 0.0161 | 0.0015 |  | 0.0012 | 0.0004 |  | 0.0223 | 0.0000 |  | 0.0168 | 0.0000 |  | 0.0036 | 0.0048 |
| C37 | 0.0481 | 0.0008 |  | 0.0458 | 0.0030 |  | 0.0638 | 0.0014 |  | 0.0278 | 0.0032 |  | 0.0340 | 0.0125 |
| G06 | 0.0762 | 0.0069 |  | 0.0347 | 0.0014 |  | 0.1012 | 0.0100 |  | 0.0894 | 0.0149 |  | 0.1132 | 0.0248 |
| G07 | 0.2016 | 0.0013 |  | 0.0639 | 0.0000 |  | 0.1239 | 0.0079 |  | 0.1391 | 0.0194 |  | 0.1280 | 0.0192 |
| G14 | 0.0544 | 0.0005 |  | 0.0081 | 0.0000 |  | 0.0102 | 0.0000 |  | 0.0128 | 0.0000 |  | 0.0277 | 0.0000 |
| G16 | 0.0646 | 0.0076 |  | 0.0986 | 0.0032 |  | 0.1106 | 0.0185 |  | 0.0522 | 0.0040 |  | 0.0426 | 0.0370 |
| G17 | 0.0275 | 0.0017 |  | 0.0172 | 0.0000 |  | 0.0000 | 0.0024 |  | 0.0144 | 0.0027 |  | 0.0000 | 0.0013 |
| G18 | 0.0368 | 0.0020 |  | 0.0338 | 0.0015 |  | 0.0242 | 0.0037 |  | 0.0308 | 0.0029 |  | 0.0281 | 0.0068 |
| G19 | 0.0289 | 0.0007 |  | 0.0120 | 0.0026 |  | 0.0193 | 0.0112 |  | 0.0232 | 0.0024 |  | 0.0747 | 0.0053 |
| G22 | 0.0516 | 0.0016 |  | 0.0224 | 0.0000 |  | 0.0528 | 0.0052 |  | 0.0409 | 0.0072 |  | 0.0498 | 0.0315 |
| G26 | 0.0000 | 0.0003 |  | 0.0000 | 0.0010 |  | 0.0026 | 0.0000 |  | 0.0007 | 0.0003 |  | 0.0025 | 0.0000 |
