# Supplementary material for: Genetic Variability of Hepatitis C Virus before and after Combined Therapy of Interferon plus Ribavirin
Source: PLoS One. 2008 Aug 26;3(8):e3058. doi: 10.1371/journal.pone.0003058 (PMC2518109; doi:10.1371/journal.pone.0003058)
Supplement: Table S8 — Relative change in the levels of synonymous and non-synonymous to synonymous substitutions in the six sub-regions of the E1-E2 region. (0.08 MB DOC) [file pone.0003058.s010.doc]

**Supplementary data**

**Table S8.** Relative change in the levels of synonymous (ΔKs=(Ks_T1_ – Ks_T0_)/Ks_T0_) and non-synonymous to synonymous (ΔKa/Ks) substitutions in the five sub-regions (NS5A_1, ISDR, PKR-BD, Rest, and V3) of the NS5A region. For patient C22 two estimates were obtained (T0_T1 and T0_T2). Values in bold type represent absolute instead of relative changes in the corresponding levels because the corresponding value at T0 was equal to 0 or not computable. (NC = non computable).

|  | NS5A_1 | |  | ISDR | |  | PKR-BD | |  | Rest | |  | V3 | |  |
| --- | --- | --- | --- | --- | --- | --- | --- | --- | --- | --- | --- | --- | --- | --- | --- |
| Patient | Δ*K_s_* | Δ*K_a_ /K_s_* |  | Δ*K_s_* | Δ*K_a_ /K_s_* |  | Δ*K_s_* | Δ*K_a_ /K_s_* |  | Δ*K_s_* | Δ*K_a_ /K_s_* |  | Δ*K_s_* | Δ*K_a_ /K_s_* |  |
| A09 | 0.4791 | **0.0168** |  | 0.5610 | -1.0000 |  | 1.3139 | **0** |  | 0.5462 | -0.0087 |  | 1.2822 | 0.3318 |  |
| A20 | -0.8379 | -1.0000 |  | -0.8043 | -0.2549 |  | 0.6061 | **0** |  | -0.3308 | -1.0000 |  | -0.4515 | -0.1940 |  |
| A21 | -0.3684 | -0.8991 |  | -0.0503 | **0** |  | -1.0000 | NC |  | 0.6425 | -0.5370 |  | -0.4237 | **1.7053** |  |
| A34 | 0.4527 | -0.0222 |  | 0.3179 | -0.1319 |  | 2.2873 | **0** |  | -0.0223 | 0.0721 |  | 3.9035 | -0.5095 |  |
| A35 | 0.0331 | **0.0387** |  | 5.3729 | **0** |  | 9.2481 | **0.0311** |  | 0.8271 | -0.1696 |  | 0.8298 | -0.3667 |  |
| C05 | 1.0929 | -0.5925 |  | 1.2459 | 2.8402 |  | 2.3082 | -1.0000 |  | 0.0417 | 0.5844 |  | 4.5256 | 6.9567 |  |
| C08 | -0.9227 | 1.5968 |  | -0.9744 | 1.9601 |  | -0.8997 | -1.0000 |  | -0.9357 | 0.1701 |  | -1.0000 | NC |  |
| C12 | -0.1282 | **0.0197** |  | -0.2944 | -1.0000 |  | 2.6595 | -1.0000 |  | 0.2978 | -0.5811 |  | 0.7254 | -0.8042 |  |
| C16 | 0.8219 | 3.3446 |  | 2.5568 | -1.0000 |  | 3.7570 | 0.3463 |  | 0.5330 | 0.0062 |  | 1.1249 | 0.5233 |  |
| C17 | **0** | NC |  | **0** | NC |  | **0** | NC |  | **0** | NC |  | -1.0000 | NC |  |
| C22T1 | -0.7735 | -0.1386 |  | -0.9550 | 18.5680 |  | -0.7670 | -1.0000 |  | -0.5519 | 0.0438 |  | 0.2775 | 0.3110 |  |
| C22T2 | -0.6284 | 0.3236 |  | -0.7938 | -1.0000 |  | -0.5431 | -1.0000 |  | -0.2381 | -0.4870 |  | 0.4622 | -0.4610 |  |
| C29 | -0.7974 | 1.0396 |  | -0.9113 | -0.8951 |  | -0.3213 | -1.0000 |  | -0.8286 | -1.0000 |  | -0.9299 | 0.3929 |  |
| C37 | 2.1741 | -0.7300 |  | 0.2488 | 0.5546 |  | 22.8696 | -0.9277 |  | -0.0249 | -0.6258 |  | 0.5053 | 1.2737 |  |
| G06 | -0.0065 | 0.1063 |  | -0.2698 | 4.5002 |  | 0.2374 | 0.0806 |  | 0.0025 | -0.0610 |  | 0.0219 | 0.0267 |  |
| G07 | 0.0218 | 0.5916 |  | 0.6915 | -1.0000 |  | 0.1032 | -0.3035 |  | 0.0166 | -0.0693 |  | -0.0386 | 1.7668 |  |
| G14 | 0.5269 | -0.8057 |  | -0.7800 | -1.0000 |  | -0.7924 | -1.0000 |  | -0.4987 | -1.0000 |  | 0.0376 | -1.0000 |  |
| G16 | **0.0646** | **0.1172** |  | **0.0986** | **0.0321** |  | **0.1106** | **0.1674** |  | **0.0522** | **0.0758** |  | **0.0426** | **0.8693** |  |
| G17 | -0.5703 | 19.7507 |  | -0.7513 | -1.0000 |  | -1.0000 | NC |  | -0.6879 | 1.5006 |  | -1.0000 | NC |  |
| G18 | -0.2771 | 0.4784 |  | -0.4033 | -0.5301 |  | 0.2503 | 0.7150 |  | -0.3809 | -0.2616 |  | -0.4847 | 9.3371 |  |
| G19 | -0.2272 | 2.6158 |  | -0.1470 | 6.2366 |  | 0.2182 | 12.9101 |  | -0.3430 | -0.0186 |  | 0.4021 | -0.8401 |  |
| G22 | 0.0707 | -0.4828 |  | -0.3520 | -1.0000 |  | 1.9974 | 0.8030 |  | 0.0396 | 0.9359 |  | 0.2459 | 0.7201 |  |
| G26 | -1.0000 | NC |  | **0.0012** | **-0.0938** |  | **0.0171** | **-0.0385** |  | **-0.0062** | **-0.0147** |  | 1.0680 | **0.1040** |  |
